# Melt reactions and timescales of melting in pelitic rocks—a case study from the Garhwal Himalaya

**DOI:** 10.1007/s00410-025-02247-z

**Published:** 2025-08-22

**Authors:** C. J. Oldman, C. J. Warren, N. B. W. Harris, B. E. Kunz, C. J. Spencer, T. W. Argles, N. M. W. Roberts, S. J. Hammond, G. Degli-Alessandrini

**Affiliations:** 1 School of Environment, Earth and Ecosystems, Faculty of Science, Technology, Engineering and Mathematics, The Open University, Walton Hall, Milton Keynes, MK6 6AA UK; 2 Department of Geological Sciences and Geological Engineering, Queen’s University, Kingston, ON KL7 3N6 Canada; 3 Geochronology and Tracers Facility, British Geological Survey, Keyworth, Nottingham, NG12 5GG UK

**Keywords:** Partial melting, Geochemistry, Petrography, Himalaya, Metamorphism

## Abstract

The nature, location, longevity and pressure-temperature conditions of different crustal melt reactions during orogenesis provide constraints on the structure, mechanical strength and exhumation of orogenic middle crust as well as element mobilisation and crustal differentiation. The Himalayan orogen offers a natural laboratory for studying crustal melting by exposing both migmatites and leucogranites in its structurally highest levels. We combine previous frameworks that link petrography or bulk geochemistry to melt reaction with in-situ trace-element analyses of large-ion lithophile elements in feldspar, mica, and garnet, U-Th-Pb isotopes in monazite and zircon and thermometry calculations in samples from the Badrinath region of the Garhwal Himalaya. Our samples naturally fall into three groups that we interpret as having formed by fluid-present melting of muscovite (Group 1, all migmatites; 650–750 °C), muscovite dehydration melting (Group 2, migmatites, leucosomes and leucogranites; 730–800 °C) and biotite dehydration melting (exemplified by a single leucogranite that contained zoned and inclusion-rich garnet and porpyroblastic K-feldspar). Geochronological data suggest that melting occurred over 20 Ma, with different samples experiencing different reactions and capturing different parts of the record at different times. Despite experiencing the same thermal history, individual outcrops typically only record one melting reaction instead of a progression through fluid-present melting followed by muscovite-dehydration melting. We interpreted this as being due to local compositional variations and availability of fluids. Our results show that petrographic observations and the mineral chemistry record are similar between (source) migmatites and (product) granites, but that fluid-present reactions are only documented in migmatites.

**Supplementary Information:**

The online version contains supplementary material available at 10.1007/s00410-025-02247-z.

## Introduction

Metamorphic reactions, particularly those that lead to the production of melt, are considered to play an important role in element mobilisation and their concentration or dilution, and in regulating crustal strength during mountain building episodes (e.g. Jamieson et al. 2011; Jamieson and Beaumont 2013; Rosenberg and Handy 2005). Linking different melting reactions in time and space allows the relative pressure-temperature evolution of the crust, the relative timing and volume of melt production and the mobilisation of trace elements to be tracked in detail (e.g. Bea 1996; Brown 2013; Kunz et al. 2022). However, the spatial disconnect between the source region of the melt and the final emplacement region of magma bodies creates a challenge for linking geochemical, petrographical and geochronological data in (source) migmatites and (product) granites to melt production, migration, mixing and crystallisation processes.

In general, metapelitic rocks can melt by fluid-present or fluid-absent (dehydration) melting reactions, most commonly involving muscovite and/or biotite - a detailed summary of these reactions is available in Weinberg and Hasalova (2015). The specific melting reaction that a rock will experience is controlled by a combination of pressure, temperature, fluid availability and mineral composition as well as the overall bulk rock composition (e.g. Bartoli 2021; Bartoli and Carvalho 2021; Brown 2007, 2010, 2013; Clemens 2012; Johnson et al. 2021; Schwindinger et al. 2019). Melting reactions may be identified petrographically (e.g. Dyck et al. 2020; Erdmann et al. 2012) and/or chemically (e.g. Gao et al. 2017; Harris et al. 1993; Patiño Douce and Harris 1998).

A common difficulty in the understanding of orogenic belts is linking the timescales of melting, transport, and crystallisation of melt from source to sink to the record of different melt reactions. As melt reactions are important for understanding both critical element mobility (e.g. Kunz et al. 2022) and tectonic evolution through their impact on deformation (e.g. Kohn et al. 2024), it is useful to constrain the progression of different melting reactions in different places through time. The Himalayan orogen preserves extensive evidence for crustal melting through the presence of genetically linked migmatites and granites (Weinberg 2016 and references therein). Migmatites and leucogranite dykes, sills and plutons are most abundant in the upper structural levels of the high-grade metamorphic core of the orogen, known as the Greater Himalayan Sequence or GHS (Searle 1999). The origin of the leucogranites is well documented and mainly linked via bulk-rock geochemical analyses to both fluid-present and fluid-absent muscovite melting reactions (e.g. Le Fort et al. 1987; Vidal et al. 1982; Weinberg 2016). A commonly held hypothesis proposes that fluid-present melting occurred earlier, during prograde metamorphism, whereas fluid-absent muscovite melting took place later, during decompression (Prince et al. 2001). The latter reaction is attributed to the formation of volumetrically significant leucogranite bodies in the Himalaya during the Miocene (Harris et al. 1995). Evidence for fluid-present melting is less common but has been recognized across the orogen. Evidence for biotite dehydration melting is sparse and is largely restricted to those regions where granulite-grade conditions have been identified (e.g. Imayama et al. ​​^2019^).

Previous work has demonstrated that minerals in migmatites and granites document the (sequence of) melt reactions which may have occurred in a particular sample through their petrographic expression (e.g. Dyck et al. 2020). For example, the presence of peritectic K-feldspar in the absence of peritectic garnet in a migmatite or leucogranite is indicative of muscovite dehydration melting, while coexisting peritectic K-feldspar and peritectic garnet (or cordierite, depending on pressure and bulk composition) are indicative of biotite dehydration melting. Peritectic grains may be distinguished from melt-crystallised grains of the same phase by examining their textures, e.g. K-feldspar textures attributed to peritectic growth include the overgrowth and replacement of plagioclase, poikiloblastic grains, and porphyroblastic grains (Dyck et al. 2020). Garnet may be identified as peritectic by the presence of very fine, randomly oriented inclusions in the cores of grains, primarily of rutile (Dorais and Spencer 2014; Dorais and Campbell 2022; Taylor and Stevens 2010).

Similarly, bulk rock element ratios of granites (and to some extent, migmatites) have been used to infer the melt reactions that formed them (e.g. Gao et al. 2017; Harris et al. 1993; Patiño Douce and Harris 1998). The large-ion-lithophile elements (LILEs) Rb, Sr, and Ba are predominantly hosted by the micas and feldspars involved in melt-producing reactions in metapelitic systems and are therefore particularly useful for reaction discrimination (Harris et al. 1995). The majority of migmatites (and some granites) are compositionally heterogeneous on a thin section or hand sample scale due to their lithological variability or transport and/or amalgamation history. Therefore, accurate determination of the equilibrated bulk volume is difficult to achieve. Additionally, identification of the minerals that contribute or withhold different elements to/from the melt can only be achieved by in-situ measurements. Here we combine the melt reaction framework afforded by petrographic insights and the chemical framework afforded by the bulk rock composition data applied to product and reactant minerals to determine whether in-situ analyses of reactant and product minerals in migmatites and granites can inform melting history.

Studying the timing and timescale of melting, transport and crystallisation on a local (sample scale) is as important as studying on a regional scale in order to track melt reactions from source to sink. Previous studies have suggested firstly that melting occurred incrementally and persistently across the Himalaya, between ca. 33 − 14 Ma (e.g. Wang et al. 2015; Ding et al. 2021; Cao et al. 2022) and secondly that the huge leucogranite plutons in the High Himalaya formed incrementally by sill injection over millions of years (Cottle et al. 2019; Harris et al. 2000; Lederer et al. 2013). The timescales of melting in the source rocks, the crystallisation of the anatectic magmas and the offset in timing between the two is still unclear at a useful level of detail and may provide insight into the rates of melting in other orogens where the older ages of the rocks, and therefore the increasing uncertainty on the analysed dates, preclude accurate resolution of anatectic processes.

What is currently missing is the link between melting reaction, geochemical evolution and chronology to constrain a more detailed temporal evolution of melting different parts of the orogen. Here we link melt reactions to major and trace element compositions of feldspars, micas, and garnet by using petrographic observations of migmatites and leucogranites exposed in the Badrinath region of the Garhwal Himalaya. This allows us to identify the chemical contributions of different minerals participating in the melting reactions. Furthermore, we link the source migmatites and product granites with U-Pb isotopic analyses of zircon and monazite to provide insights into the timing and timescales of the different melting reactions. The geochemical and geochronological data, alongside temperatures calculated using Ti-in-biotite thermometry, provide insights into the evolution and progression of metapelitic melting reactions in the crust and the petrogenesis of crustal-derived granites.

## Methods

Detailed instrument and analytical conditions are presented in Online Resource 1.

### Petrography & imaging

Optical microscopy was carried out on polished thin sections with typical thicknesses of 35–40 μm. Whole-section Backscattered Electron (BSE) and Energy Dispersive Spectroscopy (EDS) images and chemical maps were collected using an FEI Quanta 200 3D tungsten filament SEM fitted with an Oxford Instruments INCA EDS detector at the Open University. Cathodoluminescence (CL) images of zircon were acquired using a MIRA3 TESCAN fitted with a Cathodoluminescence Detector at the Guangzhou Institute of Geochemistry, Chinese Academy of Sciences. Semi-quantitative compositional maps of Y, Ce and Th concentrations in monazite grains were obtained using a field-emission Zeiss Supra 55 VP fitted with an Oxford Instrument X-Max 50 mm2 EDS detector at the Open University.

### Major & trace element analyses in major phases

The chemical compositions of major phases were collected by point and line analysis using a Zeiss Supra 55 VP field-emission SEM fitted with an Oxford Instruments X-Max 50 mm2 EDS detector at the Open University.

Concentrations of 54 major and trace elements were collected from micas, feldspars and garnet using a Photon Machines Analyte G2 193 nm excimer laser system equipped with a HelEX II laser ablation cell coupled with an Agilent 8800 Triple Quadrupole ICP-MS at the Open University. Spot analyses were run using a fluence of 3.63 J/cm^2^, a repetition rate of 10 Hz, and a spot size of 50 μm, with each analysis collecting 30 s of background, 30 s of sample ablation, and 40 s of post-ablation washout. Data reduction was carried out using Iolite v3.71 (Paton et al. 2011). Accuracy and precision of BCR-2G analyses were in good agreement with published values (Jenner and O’Neill 2012) and with relative standard deviations of 3–5% (Online Resource 5).

### Thermometry

Ti-in-biotite temperatures were calculated using the Wu and Chen (2015) calibration with a pressure estimate of 0.8 GPa (peak calculated pressure conditions in the Badrinath Formation; Spencer et al. 2012) and data from both the EDS and LA-ICP-MS datasets. The propagated uncertainty for the calibration is approximately ± 65 °C (Wu and Chen 2015).

### U-(Th)-Pb isotopes in zircon and monazite

Samples were crushed and separated at the University of Portsmouth, using a Wilfley table, Frantz magnetic separator and finally sodium polytungstate (heavy liquid). Zircon and monazite were picked from the heavy mineral fraction and mounted in epoxy resin.

U-Pb isotopes were measured in zircon rims using a Resonetics RESOltuion M-50 A-LR incorporating a Compex 102 excimer laser together with an Agilent 7700s quadrupole ICP-MS at Curtin University, Australia. Spot analyses were performed using a fluence of 1.7 J/cm^2^, a repetition rate of 7 Hz, and a spot size of 23 μm. Samples were bracketed by reference material analyses every 22 unknowns. Zircon 91500 (1062.4 ± 0.4 Wiedenbeck et al. 1995) was used as a primary standard. Plešovice (337.13 ± 0.37 Ma; Sláma et al. 2008), GJ-1 (601.92 ± 0.7 Ma; Jackson et al. 2004), and OG1 (3465.4 ± 0.6 Ma; Stern et al. 2009) were monitored as secondary standards. 91500 yielded a ^206^Pb/^238^U weighted average age of 1062.6 ± 2.4 Ma (MSWD = 0.63, *n* = 20), Plešovice yielded a ^206^Pb/^238^U weighted average age of 341.8 ± 0.5 Ma (MSWD = 2.1, *n* = 19), GJ-1 yielded a ^206^Pb/^238^U weighted average age of 616.3 ± 1.0 Ma (MSWD = 1.2, *n* = 20), and OG1 yielded a ^206^Pb/^238^U weighted average age 3491 ± 1.7 Ma (MSWD = 11, *n* = 20). Calculated average ages and uncertainties of the secondary standards overlap within 2.4% of the reference values. Full zircon U-(Th)-Pb analysis standard measurements are reported in Online Resource 6. Time-resolved mass spectra were reduced in Iolite (Paton et al. 2011). Zircon ^206^Pb/^238^U dates were corrected using the Stacey-Kramers two-stage isotope evolution model (Stacey and Kramers 1975).

U-Th-Pb isotopes in monazite were measured using an ESI/NWR193UC laser with a TV2 ablation cell coupled to a Nu Instruments Attom SC-ICP-MS at the Geochronology and Tracers Facility, British Geological Survey, UK. Material was ablated using a laser fluence of 3 J/cm^2^, a repetition rate of 10 Hz, and a spot size of ~ 11 μm. Monazite standard Bananeira (507.7 ± 1.3 Ma; Gonçalves et al. 2016) was used as the primary reference. Monazites 44069 (424.9 ± 0.4 Ma; Aleinikoff et al. 2006) and FC1 (55.7 ± 0.7 Horstwood et al. 2003) were used as the secondary standards. In session ages for 44069 yielded an average age of 430.7 ± 4.7 Ma (MSWD = 1.9, *n* = 32), and FC1 yielded an average age of 53.89 +/– 0.62 Ma (MSWD = 1.8, *n* = 37). Calculated average ages and uncertainties of the secondary standards overlap within 4.65% of the preferred values. Full monazite U-Th-Pb analysis standard measurements are reported in Online Resources 6. Data were reduced using the mean of the ratios calculated in the Attolab time-resolved analysis software. Reported monazite ^208^Pb/^232^Th dates were corrected using the Stacey-Kramers two-stage isotope evolution model (Stacey and Kramers 1975).

### Trace element analyses in accessory phases

Ti, Y, Zr, Nb, REE, Th, and U concentrations in zircon rims were measured on an Agilent 7700s quadrupole ICP-MS at Curtin University, Australia in zircon rims with ablation pits placed over the U-Pb ablation pits. Samples were ablated using a 33 μm spot size and the same laser conditions as for the U-Pb analysis. Accuracy and precision of GJ-1 zircon were accessed using published values (Piazolo et al. 2017). Our data were in good agreement (1–7%), apart from Ti, Nb and Lu, concentrations which are homogeneous lower (Nb) or higher (Ti, Lu) in our GJ-1 chip, see Online Resource 6.

Ca, Sr, Y, REE, Th, and U concentrations in monazite were measured using the same analytical set up as for U-Th-Pb isotopes at the Geochronology and Tracers Facility, British Geological Survey, UK.

## Field context and petrography

**Fig. 1:**
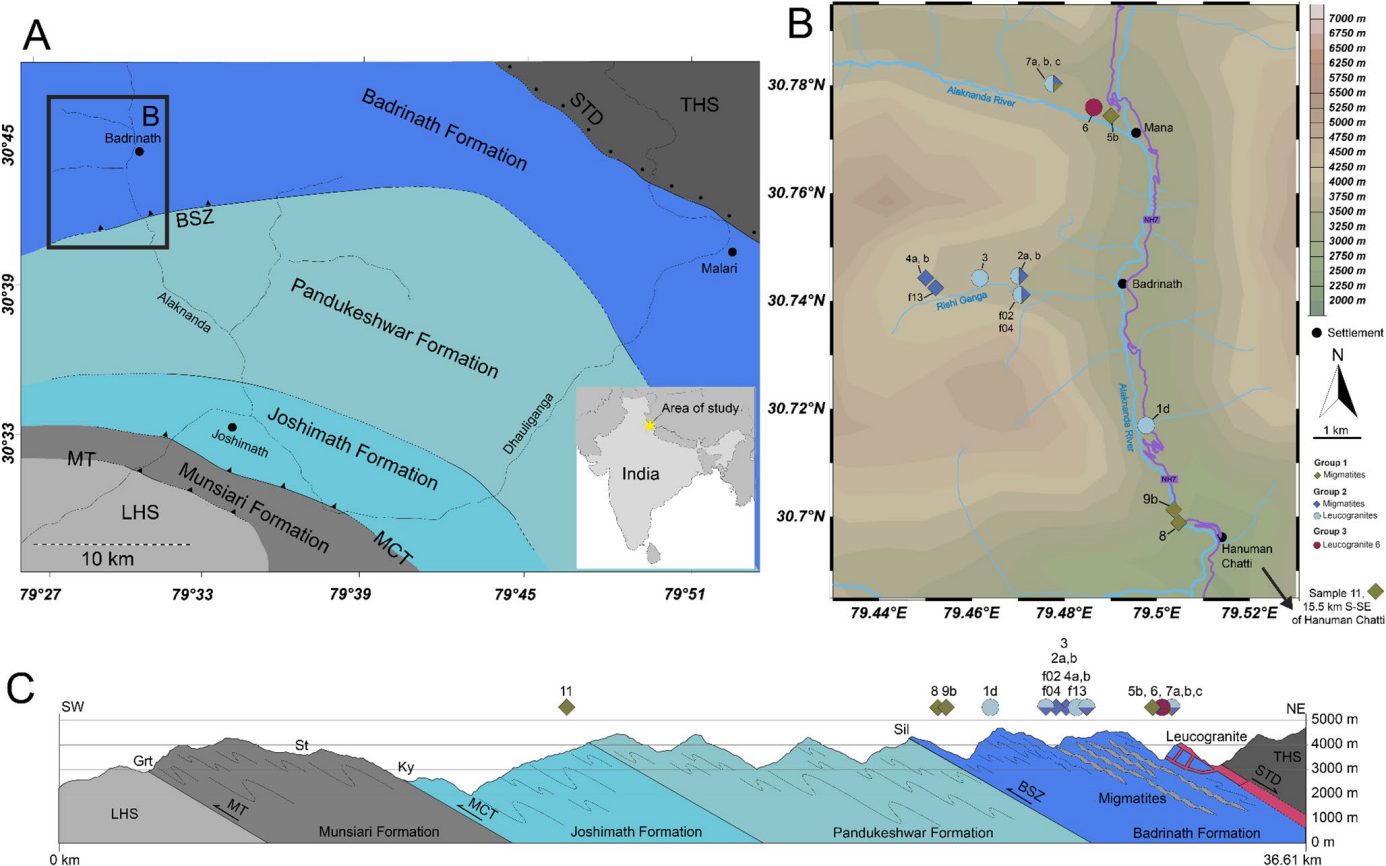
Map showing sample localities. **a** Simplified geological map of the Greater Himalayan Sequence (GHS) in the eastern Garhwal, showing the Alaknanda and Dhauliganga valleys, after Spencer et al. 2012 and references therein. **b** Map of sampling localities along the Alaknanda and Rishi Ganga valleys, around Badrinath in the Garhwal Himalaya. Coordinates are in WGS84. ** c** Simplified cross section for the Alaknanda/Dhauliganga region (after Spencer et al. 2012) with approximate samples locations. Diamonds are migmatite samples and circles shows leucogranites. Colours refer to the groups described in the text (Group 1 – green, Group 2 dark/light blue, Group 3 – pink). Locality 11 is 15.5 km south-southeast of Hanuman Chatti, near Joshimath.* THS* tethyan himalayan sequence, * STD* south tibetan detachment,* BSZ* badrinath shear zone,* MCT* main central thrust,* MT* munsiari thrust,* LHS* lesser himalayan sequence

The structurally highest portions of the Greater Himalayan Sequence in the Garhwal Himalaya (Uttarakhand, India) is divided in three units: the Joshimath (basal, composed of metapelitic schist and gneiss), Pandukeshwar (middle, composed of meta-arkose) and Badrinath (uppermost, composed of migmatitic gneiss) Formations which regionally dip towards the north (Fig. 1a). The samples described here were all collected from the Badrinath Formation, except sample 11 which comes from the Pandukeshwar Fm. The Badrinath formation is bounded at its roof by the normal-sense South Tibetan Detachment, which separates it from non-metamorphosed Tethyan Sedimentary Sequence. The Badrinath Formation gneisses generally contain quartz + biotite + plagioclase + garnet + sillimanite ± staurolite ± muscovite ± kyanite ± chlorite ± calcite ± graphite ± titanite ± rutile/ilmenite. The modal abundance of migmatites and leucogranites within the Badrinath Formation increases up-section towards the South Tibetan Detachment. For a detailed account of the local geology the reader is referred to Spencer et al. (2012) and references therein.

Studied samples collected from the Badrinath Formation include pelitic/semi-pelitic migmatites, and leucogranites that record various degrees of melting. In the field, the presence of leucogranitic material varies from in-situ, disconnected leucosome lenses (centimetre-scale) through interconnected leucosomes (metre-scale) to slightly larger granitic bodies < 1 km in extent. Full sample descriptions, field photos, locality information and mineralogy are provided in Online Resource 2.

Previous work has established a mineralogical and textural framework for distinguishing between phases formed through sub-solidus and supra-solidus metamorphic reactions and those formed during melt crystallisation (Dyck et al. 2020; Erdmann et al. 2012). These frameworks furthermore provided a basis for linking the petrographic observations to their underpinning melting reactions. Using this framework as a base, our petrographic observations suggest division of our samples into three groups.

**Fig. 2:**
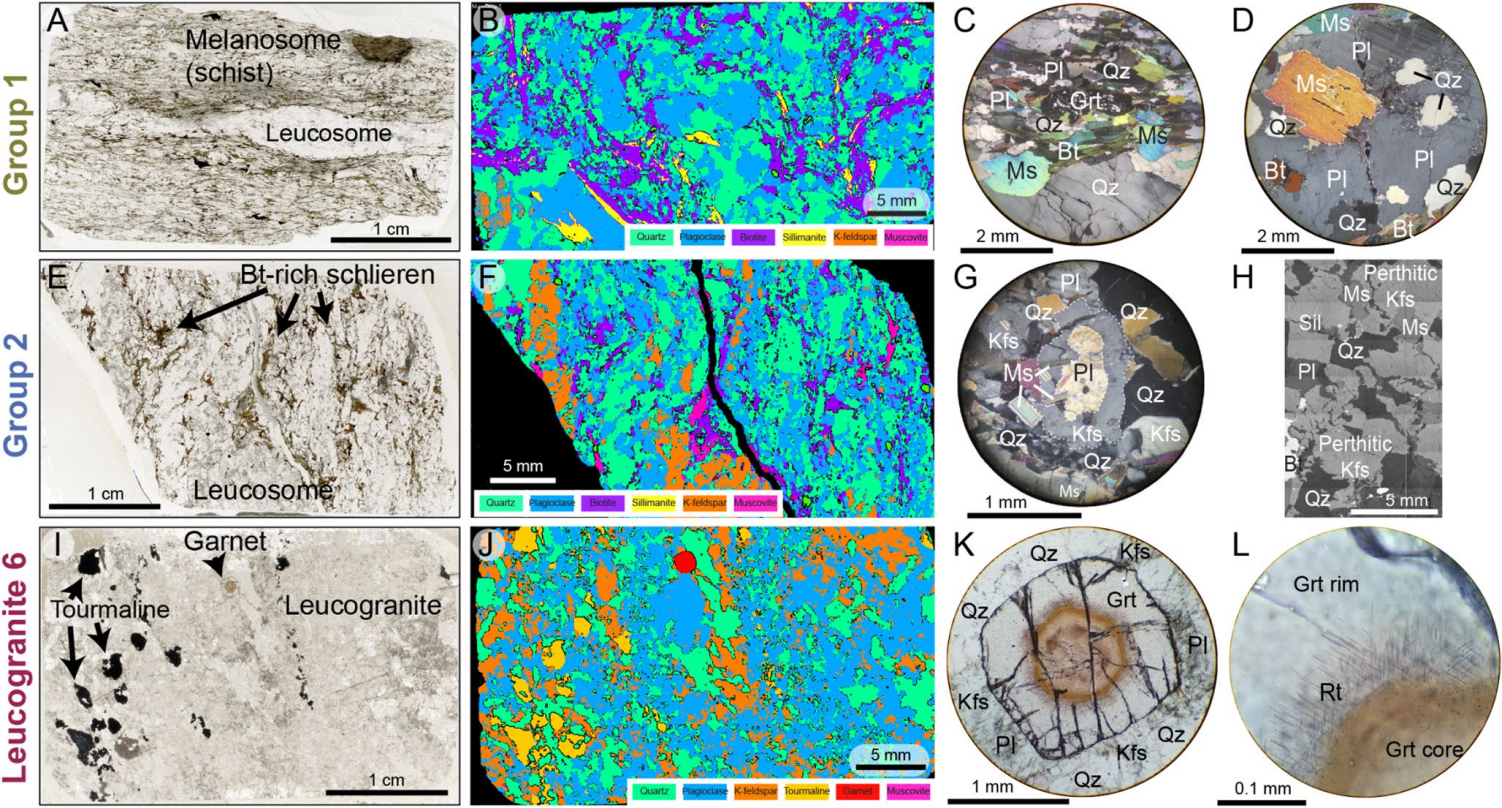
Composite images showing sample petrography for the three different groups; mineral abbreviations after Whitney and Evans (2010). **a** Thin section photomicrograph of Group 1 migmatite 8, displaying contrasting schistose melanosome domains and vein-like leucosome. **b** False colour SEM image of Group 1 migmatite 7c. The leucosomes consist of irregular areas of coarse Qz and Pl (centre and lower left). K-feldspar (Kfs) is rare and only found in the leucosome. **c** Photomicrograph of a Grt grain in Group 1 sample 9b. Garnet is in the phyllosilicate domain, elongated with the fabric and rich in Qz inclusions. **d** Coarse-grained Pl in the leucosome of Group 1 sample 11a, with inclusions of Qz + Ms + Bt. **e** Thin section photomicrograph of Group 2 migmatite 7a. **f** False colour SEM image of sample Group 2 migmatite 7a. The Kfs defines the larger patches of leucosome, commonly also rimmed by Mus-Sil intergrowths and Bt. **g** Photomicrograph of Group 2 sample 4a showing Pl with Kfs overgrowth/replacement in the melanosome. **h** BSE image of Group 2 sample 4a showing perthitic Kfs phenocrysts and Kfs overgrowing Pl. **i** Thin section image of Group 3 leucogranite sample 6, displaying zoned pleochroic Tur (left) and euhedral Grt grain (centre top). **j** False colour SEM image of Group 3 leucogranite sample 6, showing abundant and coarse-grained Kfs as well as euhedral Grt. **k** Euhedral Grt in sample 6 with a distinct core (orange) and rim (pink). **l** Crystallographically oriented rutile needles at Grt core-rim boundary in Group 3 sample 6

### Group 1 migmatites

Samples 5b, 7c, 8, 9b, and 11 contain muscovite, biotite, quartz, garnet, sillimanite and plagioclase with minor K-feldspar. They are distinguished by the presence of discrete leucosomes, either as veins or irregular, disconnected, deformed domains (Fig. 2a), and either lack, or have a low modal abundance (≤ 1%) of K-feldspar (Fig. 2b). K-feldspar is present only in leucosomes and/or as fine, isolated grains in the phyllosilicate-rich schistose parts of the rock (melanosome). Plagioclase is present in both leucosomes and melanosome (Fig. 2b). Garnet appears as elongated grains within the fabric and is rich in quartz inclusions (Fig. 2c). Muscovite is a major component in the melanosome of samples 8, 9b and 11, with only rare grains in the leucosome. Muscovite in 5b and 7c occurs as relict grains associated with biotite or associated with sillimanite around K-feldspar. Sillimanite varies from 0 to 5% modal abundance, with higher modal percentages associated with larger leucosome domains.

**Fig. 3:**
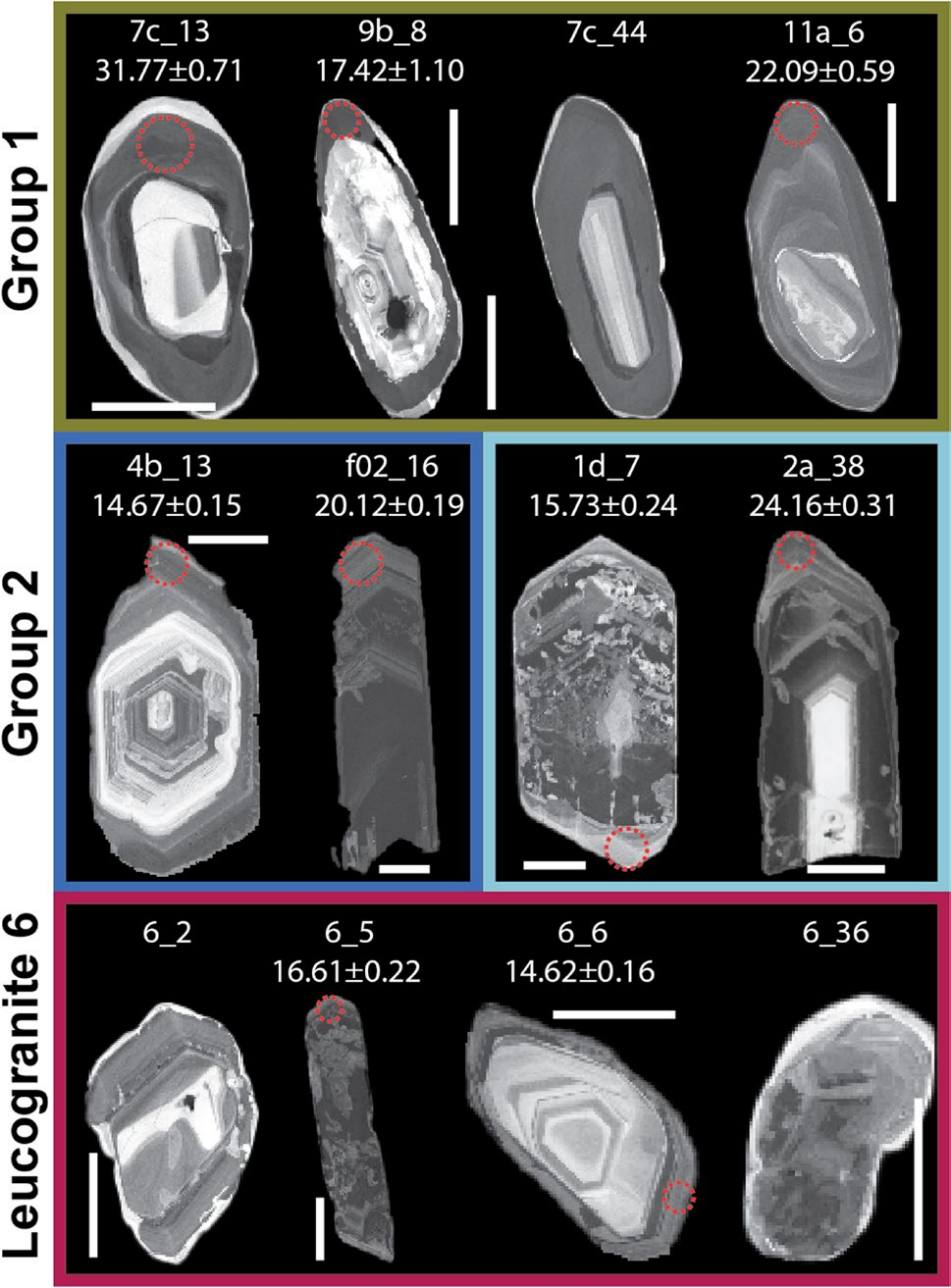
CL images showing examples of zircon grain morphology and internal zonation for samples with Group 1 high-contrast dark and bright rims; Group 2 oscillatory grey and black rims; Group 3 evidence for dissolution and only very narrow rims. Red circle indicates spot locations, the full documentation of spot locations can be found in Online Resource 3. Grain ID and ^206^Pb/^238^U dates given above image. Scale bar in all images 50 μm

Zircon in migmatites 5b, 7c, 9b, and 11a is generally present as rounded, subhedral grains between 100 and 150 μm long, typically more equant than elongate. They show a high-CL-contrast rim with an inner dark band and outer bright band and a sharp contact between the two (Fig. 3).

**Fig. 4:**
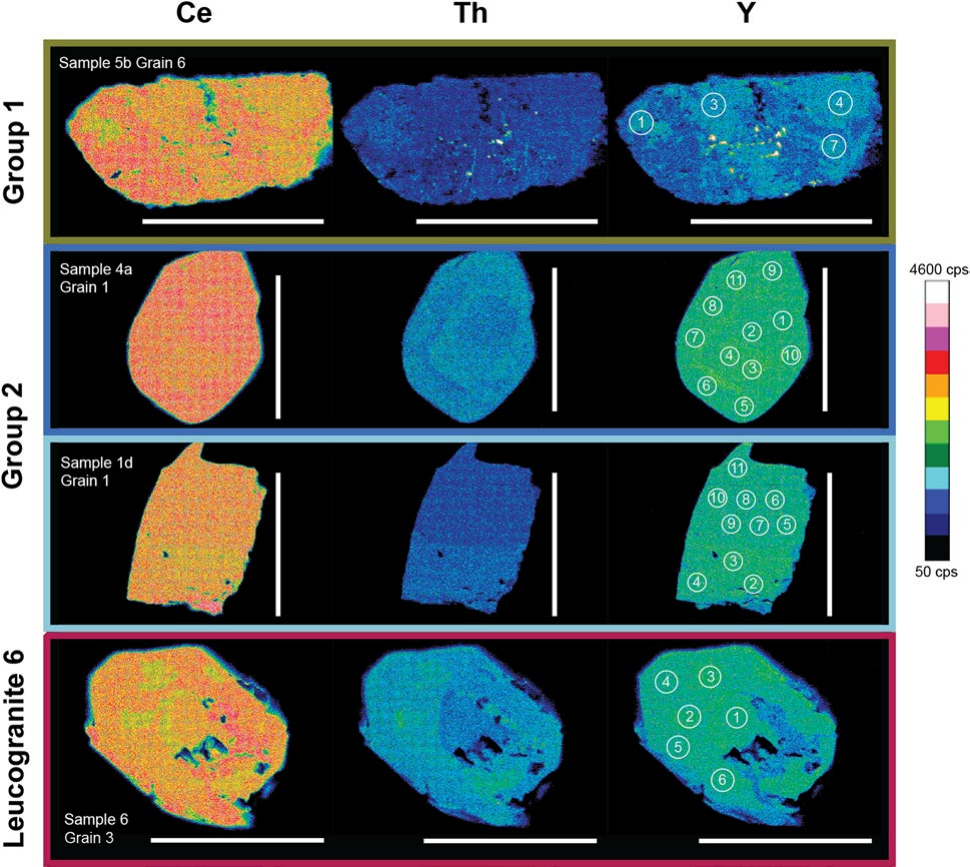
Representative selection of Ce, Th, and Y maps of monazite grains from the three sample groups. Scale bar in all images 50 μm. Irregular and patchy zone monazite in Group 1 migmatite sample 5b. Oscillatory zoned monazite in Group 2 migmatite sample 4a and no zoning in Group 2 leucogranites sample 1d. Irregular and complex zoning in Group 3 leucogranite sample 6. White circles with numbers correspond to U-Th-Pb geochronology spot locations. Full documentation of all monazites is given in Online Resource 3

Monazite separated from migmatite samples 5b, 7c, and 9b has irregular or patchwork zoning in Ce and Y concentrations (Fig. 4). Grains generally contain low and uniform Th concentrations, although sample 5b contains a few grains that show sector zoning in Th.

### Group 2 migmatites and leucogranites

Migmatite samples 2a, 4a, 4b, 7a, f02, and f13 contain muscovite, biotite, quartz, garnet, K-feldspar, sillimanite and plagioclase. In contrast with Group 1 samples, the leucosome domains have more diffuse boundaries (Fig. 2e). The K feldspar is porphyroblastic in the melanosome and phenocrystic in the leucosome, with grains > 2 mm across (Fig. 2f), perthitic albite lamellae and inclusions of anhedral quartz and embayed plagioclase. Samples 4b, 7a, f02, and f13 also contain lenticular clusters of K-feldspar grains in quartzofeldspathic domains. Plagioclase is either much less abundant than in Group 1 samples, or present as embayed inclusions within K-feldspar (Fig. 2g). Sample 4a displays topotactic replacement of plagioclase by K-feldspar (Fig. 2h). Sillimanite is present in clots associated with phyllosilicate domains (e.g. sample 7a) or as schlieren in leucosomes (e.g. sample 2a).

Leucogranite and segregated leucosome samples, 1d, 2b, 3, 7b, and f04 contain quartz, plagioclase and K-feldspar ± sillimanite, muscovite and rare garnet and biotite. They share distinctive microtextural features with the Group 2 migmatites: K-feldspar phenocrysts are perthitic, with inclusions of irregular quartz and embayed plagioclase (Figures in Online Resource 2), and are texturally similar to the porphyroblastic grains in many of the Group 2 migmatite samples (Fig. 2g, h). Fibrolitic sillimanite is present in samples 1d, 7b and f04. Garnet in sample 1d (the only garnet-bearing leucogranite in this group) is elongate and contains lobate quartz inclusions randomly distributed throughout the grains; fine inclusions (< 50 μm) of plagioclase and mica are present in garnet cores.

Zircons separated from migmatite samples 2a, 2b, 4a, 4b, 7a, f02, and f13, and leucogranite samples 1d, 3, 6, 7b, and f04 form euhedral bipyramidal 150–200 μm long crystals that are commonly fractured. Zircon grains in samples f02, f04, and f13 are notably more elongate than in other samples. Zircons have oscillatory dark rim responses in CL that become brighter towards the outer edge of the grain (Fig. 3).

Monazites separated from migmatite samples 2a, 2b, 4a, 4b, f02, and f13 and leucogranite samples 1d, 6, and f04 are either unzoned, or show simple or oscillatory zoning in Ce and Th concentrations. They typically have cores that contain higher Ce and lower Th concentrations than the rims (Fig. 4).

### Group 3 (single leucogranite)

One leucogranite sample (sample 6) presents enough distinct features to warrant its separation from the other samples. Sample 6 contains quartz, plagioclase and K-feldspar ± muscovite and rare garnet (Fig. 2i, j).

The garnet is distinctively different from garnets in the migmatites and group 2 leucogranites. Sample 6 garnets are subhedral to euhedral with strongly developed, optically visible core-rim zoning (Fig. 2k). The cores are rich in randomly-oriented < 10 μm inclusions of apatite, rutile, xenotime, and zircon, while the core-rim transition hosts crystallographically-oriented rutile needles up to 100 μm in length (Fig. 2k, l). Some pale pink garnet rims contain lobate quartz but are otherwise inclusion-poor. The porphyroblastic K-feldspar grains in this sample are petrographically identical to those in Group 2 samples.

Zircon is rare and grains are typically < 80 μm long (Fig. 3). Their morphology suggests evidence for core dissolution before crystallisation of narrow rims. Monazite grains are often complexly zoned with patch domains in Y, Ce and Th (Fig. 4). A few grains show no zoning, particularly in Y, but show a core and rim domain in Ce and Th variations.

**Table 1:** Summary of sample lithology and mineralogy within the three groups

Group	Lithology	Sample number	Mineralogy (details in Online Resource 2)
1	Migmatite	5b, 7c, 8, 9b, 11	Qz + Pl + Ms + Bt + Grt + Sil ± Kfs
2	Migmatite	2a, 4a, 4b, 7a, f02, f13	Qz + Pl + Kfs + Bt ± Sil ± Ms ± Grt
2	Leucogranite and segregated leucosomes in diatexites	1d, 2b, 3, 7b, f04	Qz + Pl + Kfs ± Sil ± Ms ± Grt ± Bt
3	Leucogranite	6	Qz + Pl + Kfs ± Ms ± Grt

## Geochemical and geochronological results

### Mineral compositions

Full major element compositions and standard data are presented in Online Resource 4. Full LA-ICP-MS trace element compositions and standard data are presented in Online Resource 5.

Of the 54 major, minor and trace elements analysed in the major minerals, we focus on Rb/Sr and Ba concentrations in K-feldspar, plagioclase, muscovite and biotite, Eu concentrations in the feldspars, and Sn concentrations in the micas. These elements were shown in previous bulk rock studies to be good indicators of melt reaction, as they are differentially hosted in either the reactant mica or the product feldspar: muscovite preferentially hosts Rb, Sr and Ba (until it starts to melt, at which point Ba partitions into the melt); biotite preferentially hosts Rb and Sn; plagioclase preferentially hosts Sr; and K-feldspar preferentially hosts Ba (e.g. Gao et al. 2017; Harris et al. 1993; Patiño Douce and Harris 1998).

### K-feldspar

**Fig. 5:**
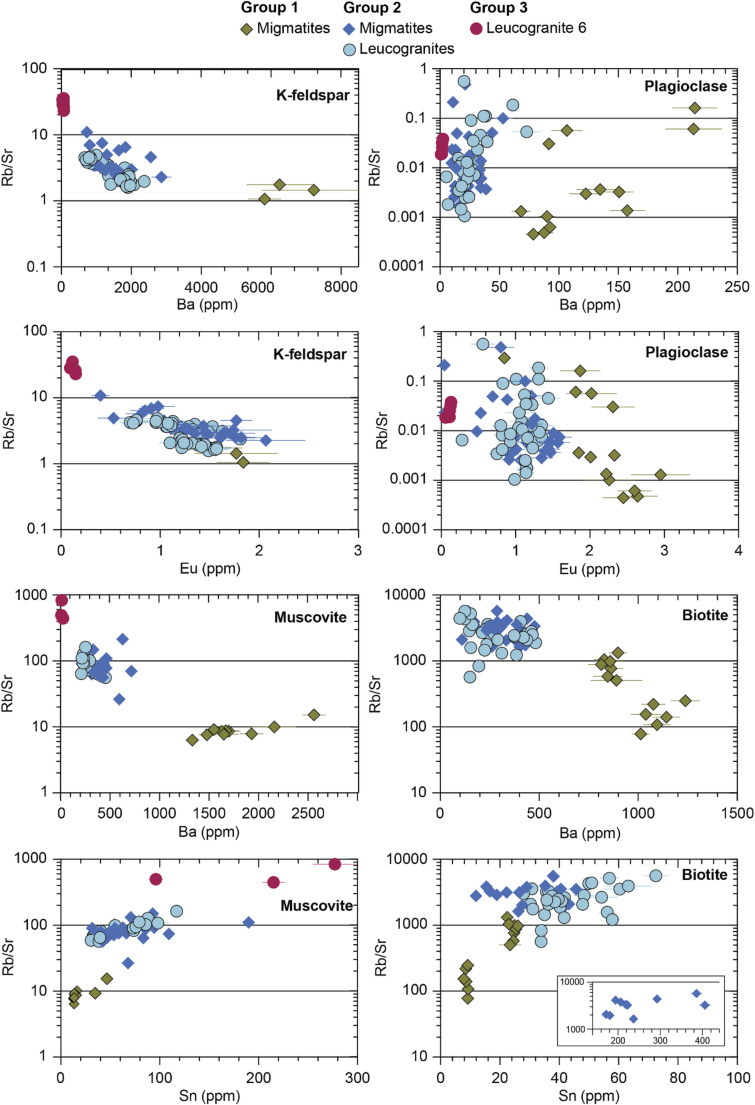
Trace element geochemistry plots of Rb/Sr vs. Ba for K-feldspar, plagioclase, muscovite and biotite and Rb/Sr vs. Sn for muscovite and biotite

Major element compositions in K-feldspar do not change between the petrographic groups, with all K-feldspar compositions ranging from Or_0.85−0.95_. In contrast, trace element concentrations cluster into the sample groups determined by petrography. K-feldspar in all lithologies contains 170–1050 ppm Rb, 16–350 ppm Sr, 15–7200 ppm Ba, 0.1–2 ppm Eu, and Rb/Sr between 1 and 35. Group 1 samples yield the lowest Rb/Sr and highest Ba and Eu concentrations. Leucogranite 6 (Group 3) yielded the highest Rb/Sr and lowest Ba and Eu concentrations (Fig. 5), with Group 2 samples yielding more of a spread in Ba and Eu concentrations and Rb/Sr.

### Plagioclase

Plagioclase shows more major and trace element compositional variation than K-feldspar across both the migmatites and leucogranites, but with no link between major element composition and petrographic group. Plagioclase in Group 1 and 2 migmatite samples 4a, 4b, 7a, 7c, 8, 9b and f02 and leucogranite samples 1d, 7b, and f04 cluster between An_0.3−0.4_; plagioclase in Group 2 migmatites 2a and f13, and leucogranite samples 2b (Group 2) and 6 (Group 3) is more sodic, clustering between An_0.2−0.3_. Plagioclase in Group 2 leucogranite sample 3 is the most calcic at ~ An_0.45_. Plagioclase in all other leucogranite samples is zoned, with oligoclase (An_0.1−0.3_) to andesine (An_0.3−0.5_) cores and more sodic (An_0 − 0.1_) rims.

Plagioclase in all lithologies contains 0.1–360 ppm Rb, 7-540 ppm Sr, 0.6–1690 ppm Ba, 0.04–3 ppm Eu, and Rb/Sr from 4 × 10^− 4^ – 1.4. Group 1 migmatites contain the highest Ba and Eu concentrations (Fig. 5), and leucogranite 6 the lowest, whereas Rb/Sr values show a similar spread across all three sample groups.

### Muscovite

Muscovite major element compositions in all samples lie within the typical range for muscovite in pelites (Forshaw and Pattison 2021). Muscovite in all lithologies contains 270–1320 ppm Rb, 0.8–50 ppm Sr, 9-8640 ppm Ba, 13–720 ppm Sn, and Rb/Sr from 6 to 842. Ba and Sn showed the strongest correlations with Rb/Sr and concentrations of these elements differ between the sample groups (Fig. 5). Muscovite in migmatite Group 1 samples contains the highest Ba concentrations and lowest Sn concentrations. Muscovite in leucogranite 6 records the highest Rb/Sr, lowest Ba and generally high Sn. Group 2 migmatite sample f13 also yielded two muscovite analyses that are particularly Sn-rich at 190 and 723 ppm.

### Biotite

No biotite was observed in leucogranite 6. Biotite in all other samples is of siderophyllite-annite composition (Deer et al. 2013) and contains 0.3–2560 ppm Rb, 0.2–74 ppm Sr, 0.1–1240 ppm Ba, 0.6–406 ppm Sn, and Rb/Sr from 78 to 5740 (Fig. 5). Biotite in Group 1 migmatites contains higher Ba concentrations and lower Rb/Sr values than those in Group 2 migmatite and leucogranites. Biotites in Group 2 migmatite f13 contain significantly higher Sn concentrations than other samples (172–406 ppm).

### Garnet

**Fig. 6:**
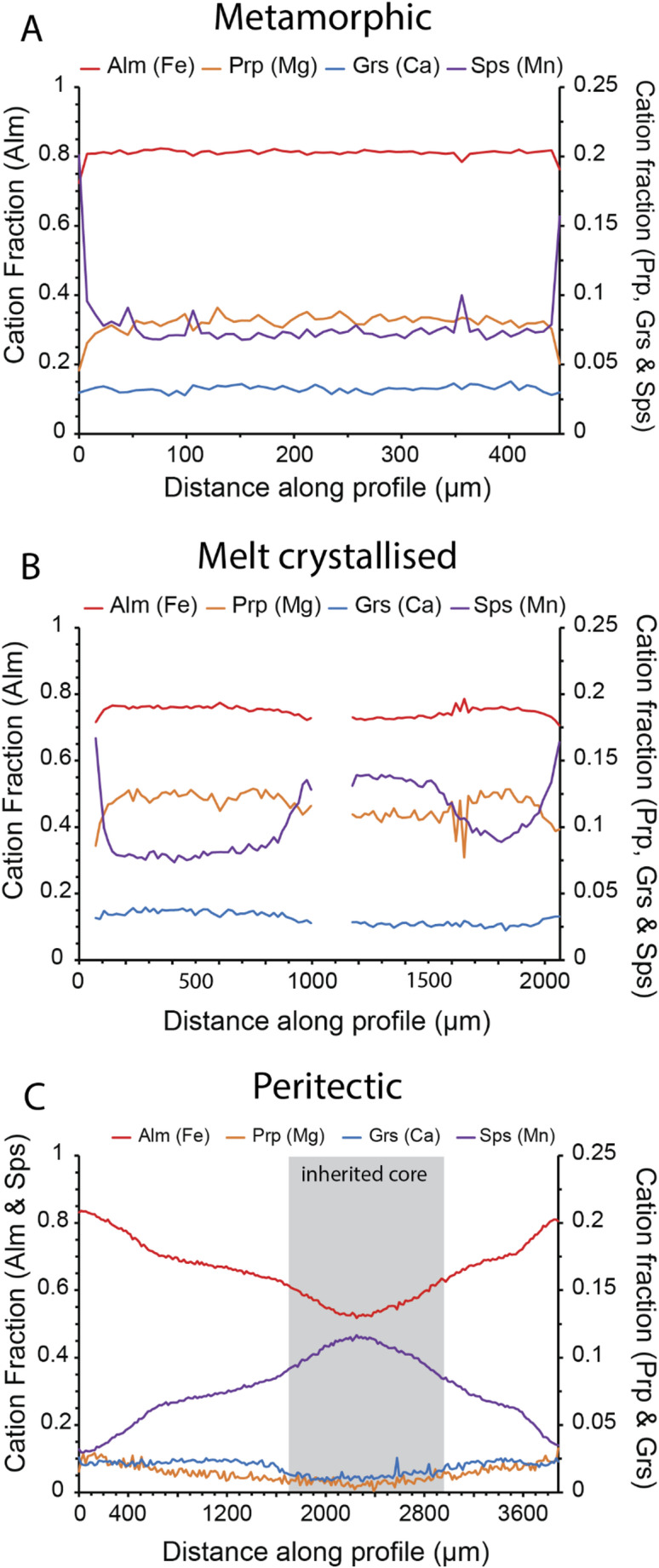
Garnet composition data.** a** Major element profile of garnet in Group 2 migmatite f02. **b** Major element profile of garnet in Group 2 leucogranite 1d. **c** Major element profile of garnet in Group 3 leucogranite 6

Garnets in the Group 1 migmatites were not analysed. Garnets in the Group 2 migmatites and leucogranites are Fe- and Mg-rich, and Ca- and Mn-poor. Migmatitic garnets record flat major element profiles except at the very outer rims (Fig. 6a). Leucogranite garnets record flat Fe, Mg and Ca profiles but commonly show cores enriched in Mn (Fig. 6b). The full garnet major element dataset is provided in Online Resource 4.

In contrast, garnets in leucogranite 6 are Mn-rich and Mg-poor and record more zoning in major elements, with core, mantle and rim zones most clearly demarcated by Mn concentrations (Fig. 6c). Garnets in sample 6 are also zoned in minor and trace elements (Online Resource 5). The core was too inclusion-rich to collect trace element concentrations of unequivocally pure garnet, and the rutile inclusions were too small to be analysed for thermometry.

### Ti-in-biotite concentrations and temperatures

**Fig. 7:**
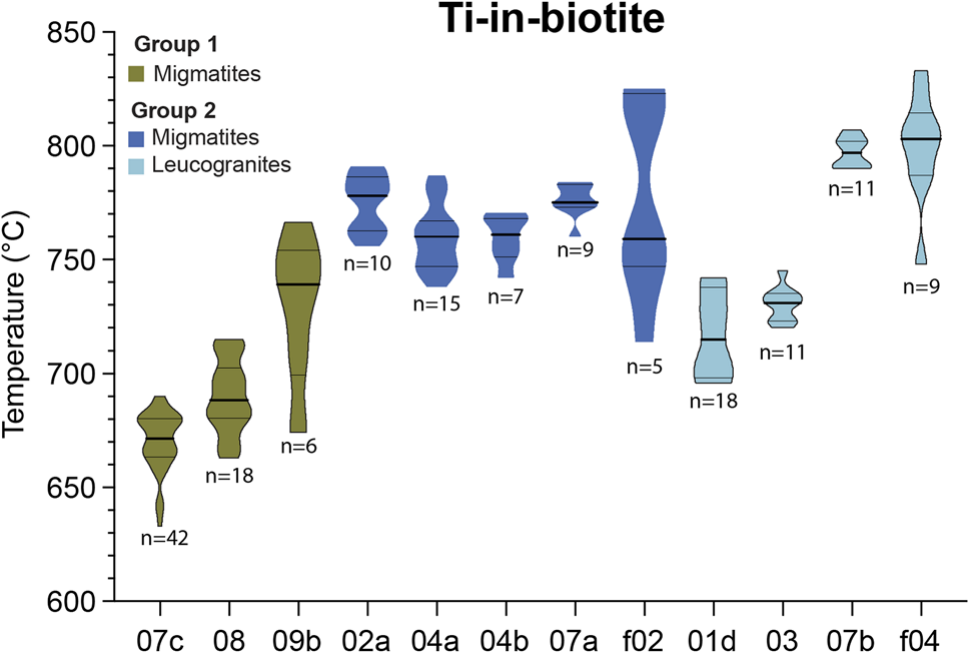
Violin plots of calculated Ti-in-Bt temperatures for Group 1 (green) and Group 2 migmatite (dark blue) and leucogranite (light blue) samples. All data are from EDS apart from samples f02 and f04, for which no EDS data were collected, therefore the LA-ICP-MS data are plotted. The lines mark the upper quartile, median and lower quartile respectively. The width of the “violins” corresponds to the number of analyses yielding that temperature value

All biotite contains Al/Si and Fe/Mg typical of siderophyllite-annite compositions (Deer et al. 2013) and most contain 0.12–0.24 Ti a.p.f.u. (Group 2 leucogranite sample 3 has elevated Ti concentrations of 0.26 Ti a.p.f.u). No biotite was present in Group 3 sample 6. The EDS and LA-ICP-MS Ti concentration datasets yield a maximum temperature difference of 20 °C. Figure 7 therefore shows the EDS data and only shows the LA-ICP-MS data for samples for which no EDS data were collected. Furthermore, Fig. 7 contain only the data for which Ti concentrations and X_Mg_ fall within the Wu and Chen (2015) calibration range. Post-peak Fe-Mg diffusion between biotite and other ferromagnesian minerals, especially garnet, may increase the Mg concentration in biotite (Kohn and Spear 2000); higher Mg concentrations will have the effect of lowering the calculated Ti-in-biotite temperatures. This effect is strongest where garnet and biotite are in contact, therefore temperatures of biotite grains in contact with garnet were not calculated. The full Ti-in-Bt (TiB) dataset is presented in Online Resource 4.

Calculated TiB temperatures across all samples range from 633 to 833 °C, with a maximum variation within any given sample of 110 °C (Fig. 7). Temperature calculations using pressures of 0.8 − 0.5 GPa to investigate potential effects on the record of decompression shift the calculated temperatures down by 20–50 °C. The majority of the sampled leucogranites are exposed as veins or intrusive sheets rather than plutons, and many have diffuse rather than sharp contacts with their host rocks, suggesting intrusion at depth. We therefore consider our temperature estimates calculated for 0.8 GPa as maximum temperatures, and acknowledge that the leucogranite samples in particular may have crystallised at lower pressures. Group 1 migmatites 7c and 8 yielded median temperatures < 700 °C, whereas Group 2 migmatites 2a, 4a, 4b, and 7a yielded median values of 750–780 °C. Group 1 migmatite sample 09b yielded an intermediate temperature, with median values of ~ 750 °C. Group 2 migmatite sample f02 yielded a notably higher median value of 810 °C and the largest intra-sample variation. Group 2 leucogranite samples 1d and 3 yielded the lowest median temperatures of 710–730 °C, while leucogranite samples 7b and f04 yielded higher median temperatures of ~ 800 °C (Fig. 7).

### Zircon U-(Th)-Pb analyses

Across 15 samples, 155 zircon analyses produce ^206^Pb/^238^U dates relevant to the timescales of the Himalayan orogen (Online Resource 6). Uncertainties on ^206^Pb/^238^U dates are typically ± 0.2–0.6 Ma (2SE).

**Fig. 8:**
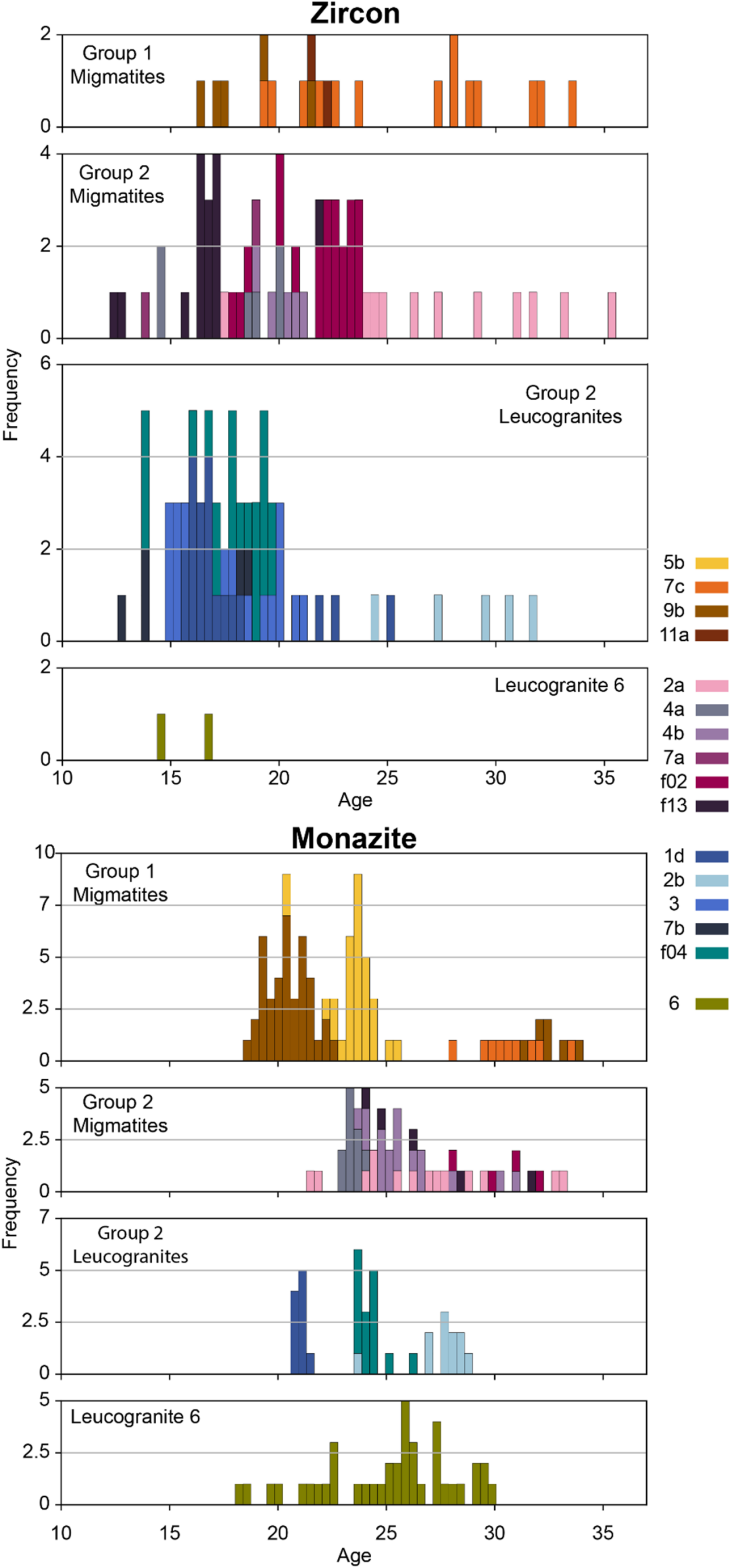
Histograms of zircon ^206^Pb/^238^U and monazite^208^Pb/^232^Th Stacey-Kramers corrected dates coloured by individual samples. Bin widths are ~ 0.5 Ma. Some samples show discrete populations while others record a more spread-out age distribution

22 zircon rims from Group 1 migmatite samples 7c, 9b, 11a yielded ^206^Pb/^238^U dates between ~ 33 Ma and ~ 16 Ma. Sample 7c yielded the widest spread in recorded dates (Fig. 8), with samples 11a and then 9b yielding more discrete date populations. 61 zircon rims from Group 2 migmatite samples 2a, 4a, 4b, 7a, f02, f13 record dates between ~ 35 Ma and ~ 12 Ma, with some samples yielding a broader spread in dates (e.g. 2a, f02) than others (4b, f13). 70 zircon rims from Group 2 leucogranite samples 1d, 2b, 3, 7b, f04 record a similar spread in dates between ~ 32 and ~ 13 Ma but with a greater cluster between 20 − 15 Ma. Sample 2b yielded a more scattered population of older dates (Fig. 8), whereas 1d, 3 and f04 yielded younger, more discrete, but overlapping populations. Leucogranite sample 6 yielded only a few grains with very narrow rims. Of these, only two analyses yielded dates with acceptable errors and common lead concentrations, providing dates of ~ 16 − 14 Ma.

### Zircon trace element analyses

**Fig. 9:**
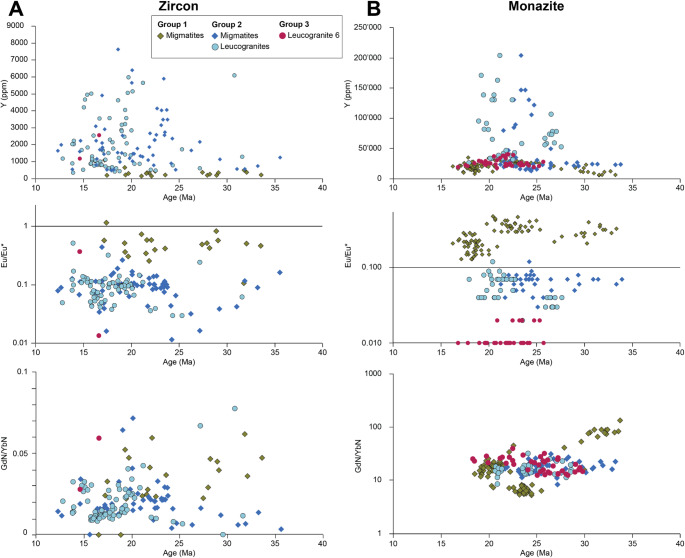
Zircon and monazite trace element vs. date colour coded by Group. **a **Although there is some general scatter for zircon in the data Group 1 shows lower Y concentrations and higher Chondrite normalised Gd/Yb ratios and Eu/Eu* compared to Group 2 and 3.** b** For the monazites Y shows scatter for some group 2 samples, Eu/Eu* is highest in group 1 and decreased for group 2 and is the lowest for leucogranite 6. Gd/Yb ratios are similar for most samples, only a few of the group 1 samples have higher and lower values

Concentrations of Ti are typically very low, with a median concentration of 1.86 ± 0.79 ppm across all samples. Y concentrations and Gd_N_/Yb_N_ are more variable, overall Y is higher in grains/zones with ^206^Pb/^238^U dates younger than ~ 25 Ma (Fig. 9). In general, concentrations of Y and HREEs are lower in Group 1 zircon than in Group 2. Eu/Eu* does not vary systematically with age (Fig. 9a). However, Group 1 zircons record variable Eu/Eu* anomalies, typically > 0.2 and < 1.0, while Group 2 zircons record more negative Eu/Eu* anomalies, typically < 0.2 (Fig. 9).

### Monazite U-(Th)-Pb analyses

213 U-(Th)-Pb analyses collected from 12 samples yielded ^208^Pb/^232^Th dates < 45 Ma (Online Resources 6). 5 analyses in Group 2 migmatite sample f13 yielded pre-Himalayan ^208^Pb/^232^Th dates between ca. 150–490 Ma. Uncertainties are typically ± 1.2–1.8 Ma (2σ). Individual age histograms for each sample are shown in Fig. 8.

86 analyses of monazites from Group 1 migmatite samples 7c, 9b, 11a yielded ^208^Pb/^232^Th dates between ~ 34 Ma and ~ 19 Ma. Each sample yielded different date ranges, with sample 7c yielding dates between 33 − 28 Ma, sample 5b between 26 − 20 Ma and sample 9b yielding two populations, between 34 − 31 Ma (one grain) and 23 − 18 Ma (9 grains, Fig. 8). 51 monazite analyses from Group 2 migmatite samples 2a, 4a, 4b, 7a, f02, f13 yielded dates between ~ 33 Ma and ~ 22 Ma, whereas 45 monazite analyses from Group 2 leucogranite samples 1d, 2b, 3, 7b, f04 yielded dates between ~ 26 and ~ 21 Ma. The monazite dates in the migmatites do not separate by sample (though sample 4a yields the youngest population, Fig. 8). However, leucogranite samples 1d, 2b and f04 each record very specific episodes of monazite crystallisation that do not substantially overlap, with sample 2b yielding dates of ca. 29 − 27 Ma, f04 of ca. 27 − 24 Ma and sample 1d of ca. 21 Ma. Leucogranite sample 6 yielded nine monazite grains, of which 40 analyses yielded dates between ~ 30 and ~ 18 Ma (Fig. 8).

### Monazite trace element analyses

Y concentrations are mostly < 50,000 ppm, though concentrations in some Group 2 samples are higher. There is no systematic variation in Y or Eu/Eu* with age (Fig. 9). Group 1 migmatites systematically record Eu/Eu* >0.1. Group 2 monazites (in both migmatites and leucogranites) record more negative Eu/Eu* anomalies, typically < 0.1 and > 0.04, and monazites in Leucogranite 6 record the lowest Eu/Eu* (Fig. 9b). Gd/Yb ratios show scattered values with no significant differences within or between groups except Gd/Yb in older group 1 monazites being higher compared to the rest of the data.

## Discussion

### Linking petrographic observations and mineral chemistry to melt reactions

Based on the frameworks outlined in previous work (e.g. Inger and Harris 1993; Dyck et al. 2020) we suggest the three groups identified by petrographic observations and confirmed through the in-situ geochemical data preserve records of different melt reactions. We have not been able to definitively determine any chemical differences between grains of different petrogenetic origin despite the main phases involved in melting (micas, feldspars and garnet) potentially having different petrogenetic origins (metamorphic, peritectic, melt-crystallised or a combination, cf. Phillips et al. 2023). This may be due to rapid chemical equilibration at high temperatures or due to the ablation volume of our analytical spots being greater than the volume of any potential trace element zoning.

#### Evidence for fluid-present melting (Group 1 samples)

Field and petrographic observations of Group 1 samples suggest that the melt formed in-situ and did not mobilise over a significant distance (likely < 1 m). Petrographic observations document a lack of peritectic K-feldspar and a modal abundance of melt-crystallised K-feldspar per volume of leucosome lower than 4:5 (cf. Dyck et al. 2020). The micas (now predominantly biotite), garnet and plagioclase in the melanosome formed during sub-solidus metamorphic reactions, whereas the K-feldspar plagioclase and rare muscovite in the leucosome most likely formed from the crystallisation of melt.

The low Rb/Sr in the Group 1 minerals suggests a low ratio of muscovite to plagioclase during melting (Harris et al. 1995), which in turn suggests that more Sr and Eu were released into the melt from plagioclase than Rb and Sn from muscovite (e.g. Gao et al. 2017; Patiño Douce and Harris 1998). The high concentration of Ba in Group 1 sample minerals can be explained by the breakdown of muscovite which is the main host for it in sub-solidus schists (Harris et al. 1995). Due to the small amount of K-feldspar (the preferred sink for Ba) in these samples, the Ba concentrations are high not only in K-feldspar but also in plagioclase and micas compared to those in the Group 2 samples. These data mirror previous bulk rock granite datasets that were interpreted as formed through fluid-present melting reactions (Gao et al. 2017; Harris et al. 1993; Inger and Harris 1993; Ji et al. 2023). We therefore interpret Group 1 samples as recording melting through predominant fluid-present muscovite melting reactions.

The textures and REE concentrations of the Group 1 zircon rims are furthermore also typical of grains crystallised or grown from a melt; for example oscillatory zoning, HREE-enrichment, positive Ce anomalies, and negative Eu anomalies (Hinton and Upton 1991; Murali et al. 1983; Rubatto 2002, 2017; Trail et al. 2012). The lower concentrations of Y and Lu in zircon in Group 1 samples is consistent with zircon growth in the presence of garnet (an Y and HREE scavenger; e.g. Rubatto and Hermann 2007). Zircon in Group 1 samples crystalised over a timescale of ca. 20 Ma, but with different samples recording zircon crystallisation at discrete intervals. This suggests that these samples melted in the presence of fluids at different times, with the zircon dates reflecting the timing of (local) zirconium saturation in the melt (which facilitated zircon crystallisation).

The monazite grains in Group 1 samples commonly show mottled and patch zoning patterns, dissimilar to those commonly reported from metamorphic growth in sub-solidus metapelites in the Himalaya (e.g. Foster et al. 2002; Rubatto et al. 2013; Warren et al. 2019) and more similar to those interpreted as having grown from, or been affected by, fluids (e.g. Schulz 2021). Monazite dates in Group 1 samples cluster by sample, with samples either yielding clustered single populations (e.g. samples 7c and 5b) or bimodal populations (e.g. 9b). Sample 9b was collected near the Badrinath Shear Zone, with supra-solidus conditions and monazite crystallisation at ~ 19 Ma reported by Benetti et al. (2021).

Together, the petrographic, chemical, thermal and chronological data suggest that Group 1 samples experienced fluid-present melting (and fluid-present melting only) in different parts of the Garhwal at different times. Metapelites will only experience fluid-present melting if there is sufficient availability of fluid at the appropriate P-T conditions. In Group 1 samples, there is no textural or chemical evidence that the migmatites experienced muscovite dehydration melting after fluid-present melting despite regional temperatures that facilitated muscovite dehydration in the surrounding area – probably because muscovite in the Group 1 rocks was completely consumed during fluid-present melting. Nor is there a thermal record of higher T captured by the biotite, suggesting that the biotite failed to re-equilibrate at higher temperatures after melting ceased. Hence once melting was complete (muscovite consumed) we suggest there was little additional chemical re-equilibration. The lack of textural or chemical evidence that Group 2 migmatites experienced fluid-present melting before muscovite dehydration melting (further explored below) additionally suggests that fluid infiltration was localised.

Group 1 migmatite samples 5b and 9b (collected from the northern and southern ends of the field area respectively, Fig. 1) record zircon and monazite ages between ca. 25 − 18 Ma that overlap and are younger than monazite dates in Group 2 migmatites and leucogranites and which overlap with Group 2 zircon dates (Fig. 8). This could either imply that fluid-present and dehydration melting reactions were taking place synchronously in samples of subtly different bulk composition or due to different sources of fluid, that temperatures increased asynchronously across the region, or that the accessory geochronometer phases record a crystallisation event not captured in the chemistry of other phases. However, samples 5b and 7c, collected within a few kilometers of each other, record very different monazite age spans for the same reaction. We therefore interpret the monazites to record the timing of intermittent fluid pulses driving melting in rocks of suitable bulk composition (potentially those with lower initial modal abundance of muscovite) at different times across the region. More work is required to constrain the source of the fluids, but fluids released from nearby melts that were crystallising at the same time during regional exhumation/cooling provide a potential source.

The Garhwal Himalaya do not appear to expose leucogranite bodies that were formed exclusively by fluid-present melting, likely due to the low volumes of melts produced by fluid-present melting or the mixing of those melt with other melts. However, leucogranites recording geochemical signatures of (or geochemical trends towards) fluid-present melting have been reported elsewhere in the Himalaya, particularly east of the Everest region and in SE Tibet (e.g. Gao et al. 2017, 2024).

#### Evidence for muscovite dehydration reactions (Group 2 samples)

In contrast to the Group 1 samples, Group 2 migmatites and leucogranites record diffuse boundaries between the melanosome and leucosome, irregular and embayed plagioclase, perthitic porphyroblastic K-feldspar and K-feldspar porphyroblasts with poikiloblastic cores. Petrographic observations of the K-feldspar porphyroblasts in particular show that (1) they preferentially occur along Pl-Pl and Pl-Qz boundaries, with preferred nucleation of K-feldspar on plagioclase in quartzofeldspathic domains; (2) their relationship with plagioclase is topotactic, with epitaxial K-feldspar nucleation (e.g. sample 4a); (3) they grew continuously at the expense of plagioclase in quartzofeldspathic domains; and (4) grains show clustering and annealing to form poikiloblasts with common inclusions of quartz and biotite (samples 4b, 7a, f02, and f13). This combined petrographic evidence for peritectic crystallisation of K-feldspar plus evidence for growth, increased mode and textural development is consistent with increasing melt production as described by Dyck et al. (2020), and suggests these samples record evidence for muscovite dehydration melting reactions.

Furthermore, muscovite and biotite record higher Rb/Sr, higher Sn and lower Ba concentrations compared to the same minerals in Group 1 samples. K-feldspar has higher Rb/Sr, and both K-feldspar and plagioclase have lower Ba and Eu concentrations. These signatures mirror bulk rock data from granites that were interpreted as forming from melts created by muscovite dehydration reactions (Harris et al. 1993; Inger and Harris 1993; Gao et al. 2017; Ji et al. 2023). We therefore interpret Group 2 samples to record melting through progressive muscovite dehydration reactions.

Biotite, plagioclase, K-feldspar and muscovite in Group 2 samples show a significant spread in trace element concentrations both within and between samples, and between migmatites and granites. This spread may indicate variability in protolith bulk composition, a mixture of petrogenetic mineral origins (e.g. metamorphic, peritectic or crystallised from melt) or growth zoning formed during the progression of the melting reaction (e.g. peritectic cores and melt-crystallised rims, or multiple melt-crystallised zones that capture chemical evolution of the melt). In particular, our dataset shows no clear links between K-feldspar evolution in a single sample and chemical trend, nor clear differentiation between the chemical composition of K-feldspar in the source migmatites (potentially more weighted towards peritectic formation) and the composition in the leucogranites (potentially more weighted towards magmatic formation). The overlap in petrographic and chemical signatures of minerals in the source migmatites and coherent granites suggests little additional chemical differentiation during melt mobilisation. Additionally, the chemical similarity between reactant and product phases in the migmatites suggests chemical equilibration over the timescales of melting and cooling.

**Fig. 10:**
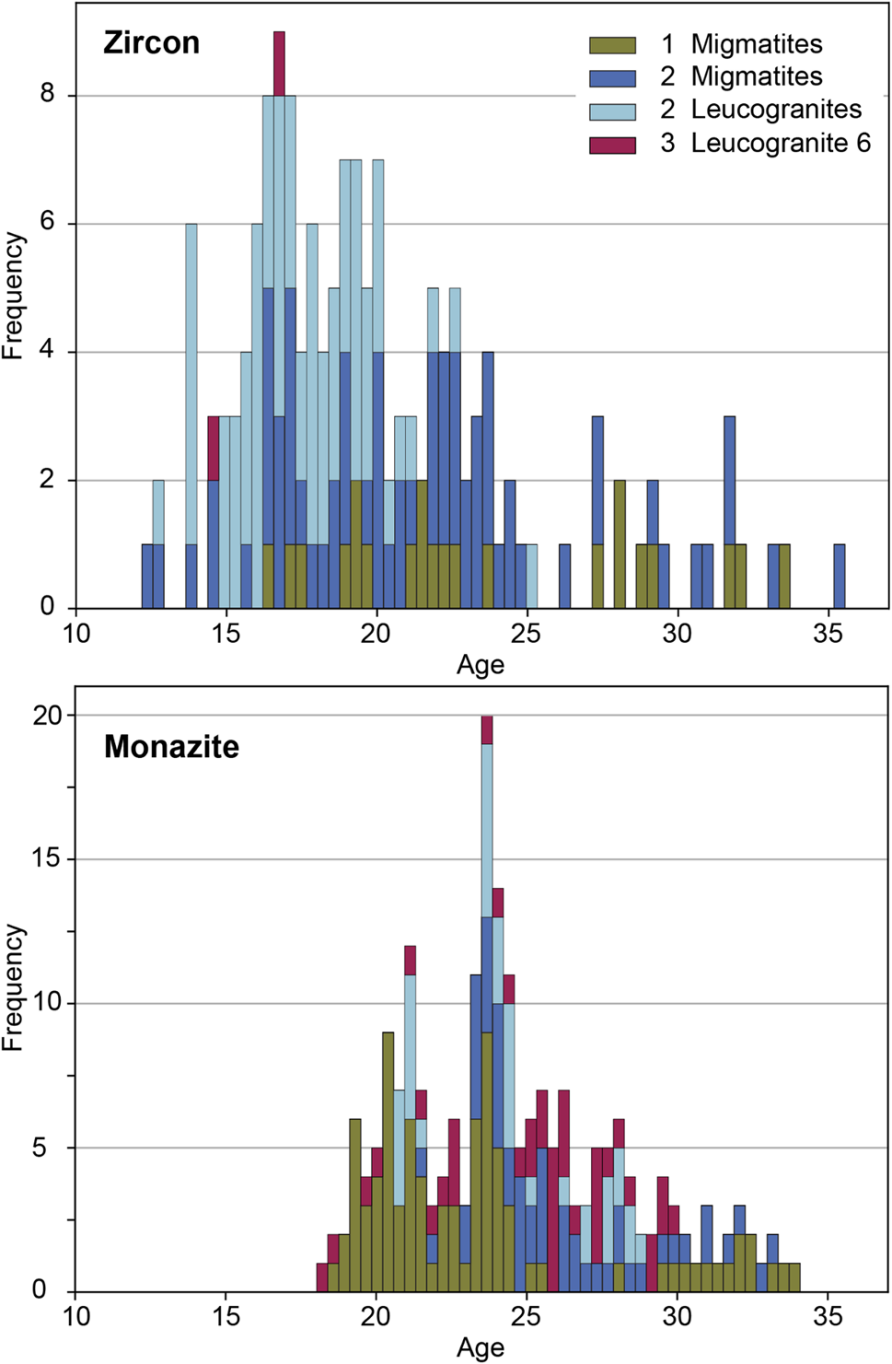
Stacked histograms of zircon ^206^Pb/^238^U and monazite^208^Pb/^232^Th Stacey-Kramers-corrected dates coloured by melt reaction groups. Bin widths are ~ 0.5 Ma, zircon n = 155, monazite n = 213

Zircon in Group 2 samples crystallised over a similarly long timescale to Group 1 samples (Figs. 8 and 10), but with more pronounced pulses of crystallisation over ca. 23 − 17 Ma (migmatites) and 20 − 15 Ma (leucogranites). The higher concentrations of Y and lower Gd/Yb ratios in Group 2 sample zircon is consistent with the release of these elements into the melt during breakdown of garnet (e.g. Rubatto and Hermann 2007; Yakymchuk 2023) - the increasing Y concentrations in zircon suggest decreasing garnet stability coincident with increasing anatectic melt volumes. In a Himalayan context, the breakdown of garnet during muscovite dehydration melting is consistent with the hypothesis that melting was triggered by isothermal decompression (e.g. Harris and Massey 1994). Furthermore, the more negative Eu anomaly in zircons in Group 2 samples is consistent with the increased sequestration of Eu in (peritectic) K-feldspar (e.g. Hinton and Upton 1991; Gardien et al. 1995; Murali et al. 1983; Patiño Douce and Harris 1998; Pickering and Johnston 1998; Rubatto 2002). Overall, the U-Pb zircon data suggest that the (source) migmatites melted and crystallised before the (sink) leucogranites (which makes inherent geological sense), but there is a considerable period of overlap and a distinct peak of zircon crystallisation in both lithologies at ca. 17 Ma. Each sample records a narrower and different age record (e.g. sample 2a yields older dates than sample f13 which in turn is slightly older than 4b and then f02; Fig. 8). The date ranges are linked to the continuum between “leucosome” and “leucogranite”, with sample 2a (earlier crystallisation) described in the field as a leucosome of a diatexite (Supplementary Material 3), whereas the other samples, described as leucogranites in the field, had intrusive relationships that suggest that the melt had travelled some distance from its source. Zircon dates from both individual samples and the collective record suggest that different samples melted, mobilised and crystallised at different times, either due to diachronous thermal histories or due to differences in bulk composition leading to the melting reactions initiating at different times.

Monazite grains in both the migmatites and leucogranites commonly show oscillatory zoning, suggestive of growth within a melt. Compared to the zircon record, monazite in Group 2 samples overall record older dates (Figs. 8 and 10). This suggests anatectic melt became saturated in LREE prior to saturation in Zr, with monazite dissolving more readily than zircon and therefore more rapidly saturating the melt (Yakymchuk and Brown 2014).

#### Biotite dehydration melting (Leucogranite sample 6)

In this study, only a single leucogranite (sample 6) was identified as presenting a significantly distinct petrography compared to the Group 2 leucogranites to warrant its separate classification. No migmatites with similar garnet texture and composition were identified in the region, which means the source region of this granite is likely not exposed.

The Mn-rich and inclusion-rich cores of garnets in this sample suggest formation via a peritectic reaction (Dorais and Campbell 2022; Dorais and Spencer 2014; Taylor and Stevens 2010), a theory supported by the petrographic similarity of porphyroblastic K-feldspar grains in this sample to those of peritectic origin in Group 2 samples. The garnet mantles with crystallographically-oriented rutile needles and rims containing lobate quartz inclusions suggest crystallisation from a melt rather than entrainment of metamorphic grains (Jung et al. 2022; Taylor and Stevens 2010). Compared to the minerals in Group 1 and 2 samples, all analysed minerals had higher Rb/Sr and very low Ba concentrations, which mirror the bulk-rock signatures of granites that have previously been interpreted as forming through biotite dehydration reactions (Gao et al. 2017; Harris et al. 1993; Inger and Harris 1993; Ji et al. 2023). The presence of both peritectic K-feldspar and garnet lend further support to the suggestion that leucogranite 6 formed through the biotite dehydration reaction (Breton and Thompson 1988a, b; Harris et al. 1995).

Sample 6 contains no biotite, thus precluding the calculation of Ti-in-biotite temperatures. However, garnets in this sample contain crystallographically oriented rutile needles surrounding the core. This feature is commonly associated with garnet crystallisation temperatures of > 800 °C (Axler and Ague 2015; Hwang et al. 2007; Proyer et al. 2013). At these temperatures Ti is more soluble in garnet; later cooling drives exsolution and crystallisation of rutile needles along the garnet (111) plane (Hwang et al. 2016). The high initial Ti concentrations in garnet in sample 6 therefore suggest higher peak temperature conditions than the other samples reported here, and further support our interpretation of melting through a biotite-dehydration reaction.

Zircon was rare in sample 6 and only yielded two (relatively young) dates. Monazite showed oscillatory but patchy zoning, and a timespan of crystallisation that is similar to samples in Groups 1 and 2. However the limited data from this sample preclude a firm interpretation.

### In-situ mineral chemistry vs. bulk rock chemistry

An important difference from previous geochemical studies (e.g.Harris et al. 1993; Inger and Harris 1993; Gao et al. 2017; Ji et al. 2023) is that our Rb, Sr, Ba and Sn geochemical dataset is derived from individual minerals rather than from bulk rock analyses. While these minerals make up the main budget for these trace elements in the bulk rock, the individual mineral proportions determine the whole rock composition. Therefore, it is noteworthy (although perhaps not surprising) that individual minerals in both migmatites and leucogranites seem to follow the same discrimination patterns as the bulk rock in Rb/Sr vs. Ba plots. A few studies have questioned the use of these geochemical discrimination vectors to distinguish between fluid present and fluid absent melting (e.g. Schwindinger et al. 2019; Bartoli 2021). Factors such as P-T conditions, fluid or bulk rock composition and fractional crystallisation can all control the geochemical signatures that minerals and rocks record.

However, contrary to previously published bulk rock granite studies, our samples represent both the suggested source and sink of the melt and our petrographic evidence for melting reactions allow cross referencing with the geochemical results. Furthermore, discrimination of melting reaction based on (bulk rock) geochemistry is more likely to be successful in the Himalaya than in most other orogens because the granite plutons appear to be generally sourced from a single melt reaction in a relatively homogeneous source region, rather than the product of multiple melting reactions in a more heterogeneous source. Our geochronological results support this hypothesis in suggesting that melting reactions occur episodically on a sample scale but continuously on a regional scale. In other orogenic regions, with potentially more complex (and/or higher temperature) melting histories (due to e.g. mixing, fractionation), trends in Rb/Sr vs. Ba are less clear. More detailed work pairing petrographic and mineral-scale trace element datasets in migmatites is needed to investigate the full potential of the mineral level in-situ approach as well as determine its limitations.

### Thermal record

Overall, titanium-in-biotite thermometry results yield temperatures that correlate with petrographic observations of fluid-present and muscovite dehydration melting in both the migmatites and leucogranites. The temperatures are also similar to previous T estimates for the upper GHS in Garhwal (e.g. 690–740 °C, Kawabata et al. 2021; ≥ 750 °C, Iaccarino et al. 2017; ≤757 ± 8 °C, Manickavasagam et al. 1999). Group 1 migmatite temperatures suggest equilibration at relatively low temperatures (< 700 °C, Fig. 7), consistent with phase diagram predictions of fluid-present melting reactions (e.g. Johannes and Holtz 1990). Group 2 migmatites typically record temperatures > 750 °C, consistent with that expected for the relative temperatures of fluid-present and muscovite dehydration reactions between 1 and 0.8 GPa (e.g. Clemens and Vielzeuf 1987; Le Breton and Thompson 1988a, b). We note that even if a lower pressure (e.g. 0.5 GPa) is used to calculate the temperatures of the Group 2 leucogranites (to reflect their likely crystallisation at higher crustal levels than their source migmatites), their calculated temperatures remain higher than those calculated for the Group 1 migmatites.

Our sample collection contains examples of different melt reactions and different thermal records preserved within a few meters of each other in the same outcrop. For example Group 1 migmatite sample 7c was collected from within a meter of Group 2 migmatite sample 7a, and a similar overall thermal evolution, and similar peak temperatures would therefore be expected. However, sample 7c records a lower average temperature than sample 7a (680 °C vs. 780 °C, Fig. 7). The recorded temperatures are consistent with the expected prograde thermal evolution for the melting reactions the samples record (fluid-present in sample 7c and muscovite dehydration in sample 7a) but imply either that biotite in 7c did not re-equilibrate after early fluid-present melting, or that later fluid-present melting was triggered in 7c, e.g. by fluids released during crystallisation of the melt in migmatite sample 7a or the leucogranite sample 7b. Sample 7c yields older zircon and monazite dates than samples 7a and 7b (ca. 30 Ma vs. ca. 20 − 14 Ma), suggesting that fluid-present melting preceded dehydration melting in this area (assuming the zircon crystallised during melting).

The titanium-in-biotite record in sample 7c therefore suggests that biotite did not chemically re-equilibrate at higher temperatures after cessation of the fluid-present melting which reacted out most of the muscovite. This may have been due to lower *a*_TiO₂_ compared to that in sample 7a. Previous studies suggest that*a*_TiO₂_ may be reduced by the presence of melt: modelling from granitoid and rhyolitic melt suggests *a*_TiO₂_ drops to < 0.5 relative to rutile saturation (Borisov and Aranovich 2020; Schiller and Finger 2019). As such, the Ti-in-biotite temperature may record the point at which the presence of interstitial melt reduces the effective transfer of Ti^2+^ and Mg^2+^ ions between biotite and the matrix. Later increases in temperature of such previously-melted (but later crystallised) rocks may therefore not be recorded by changes in titanium concentrations in biotite. We therefore suggest that after cessation of the fluid-present melting which reacted out most of the muscovite, biotite in sample 7c failed to re-equilibrate at higher temperatures – maybe due to lower *a*_TiO₂_ compared to the Group 2 samples.

### Geochronological evolution and geochronometer petrogenesis

All three melt-reaction sample groups contain zircon and monazite that record crystallisation from ca. 36 − 12 Ma, with the migmatites in general recording an earlier and broader spread of dates and the leucogranites a later and narrower spread (Fig. 10). Uncertainties on individual ages are sufficiently small that the spread of ages is likely to be real rather than an analytical artifact. Hence, we interpret the scatter in the geochronology data to reflect the timescales of incremental monazite and zircon growth during orogenesis. These time ranges suggest that metamorphism and melting in the Garhwal Himalaya took place over a similar period to the timescales of sub-and supra-solidus metamorphism documented elsewhere across the eastern Himalaya (e.g. Ding et al. 2021; Hopkinson et al. 2020; Rubatto et al. 2013). Importantly, the data do not support the standard contention that fluid-present melting systematically took place before fluid-absent melting in the same rock volume (Prince et al. 2001). The spread of ages, and thus the timing and extent of monazite and zircon crystallisation both within and between samples that present similar petrographic and geochemical indicators, is the consequence of several factors that pertain across the lengthscale of a few kilometres. These include (1) the different melting reactions occurring in (subtly) different bulk compositions at different times, (2) differing bulk and local concentrations of Zr, Ce and P, and (3) localised distribution of fluids from different sources at different times.

Zircon does not readily crystallise during sub-solidus metamorphic reactions and more readily crystallises from melt (Kelsey et al. 2008; Rubatto 2017 and references therein; Yakymchuk and Brown 2014). In contrast, monazite crystallises readily during sub-solidus reactions in metapelites, is considered to readily dissolve as anatectic melt is formed, and then re-crystallises during the crystallisation of the melt when P and Ce concentrations reach saturation levels (e.g. Engi 2017 and references therein; Kelsey et al. 2008; Yakymchuk and Brown 2014).

It is therefore important to determine the petrogenesis of both zircon and monazite in each sample to be able to interpret (1) when the geochronometer minerals crystallised and (2) the petrologic significance of the ^208^Pb/^232^Th date. Where observed in thin section, zircon was more commonly present in the leucosomes, whereas monazite was present in both melanosomes and leucosomes. While the lack of petrographic context limits detailed interpretational possibilities, the general trends, together with the petrographic and geochemical signatures, allow the melting reactions to be put into temporal context.

Therefore, we interpret the zircon dates to represent the timing of zircon crystallisation in the melt. Based on the compositional maps there is no clear link between date and Y, Th or Ce zoning pattern in monazite, however Gd/Yb ratios in monazite decrease after about 27 Ma. Eu/Eu* are linked to sample groups but not to date. In general, we also interpret the majority of the monazite dates to represent the timing of crystallisation in melt (cf. Wang et al. 2017). The increasing Y concentrations in zircon and Gd/Yb ratios in monazite after 27 Ma are consistent with growth during garnet breakdown, observed in many Himalayan transects (e.g. Imayama et al. 2012; Rubatto et al. 2013; Wang et al. 2015). However more detailed investigation and in situ analysis would be necessary to confirm these trends.

Sample 09b monazites yielded a bimodal age pattern, raising the possibility that this sample preserves a record of sub-solidus growth. However, the older 33 − 31 Ma ages were only recorded in one grain, precluding firm interpretations.

### Melting history in the Garhwal Himalaya

The prevalence through time of the three melt reactions identified in samples from this study can be examined by grouping zircon and monazite ages by melt reaction associated with each sample (Fig. 10). Under the assumption that (most) zircon and monazite dates reflect the timing of crystallisation in the presence of melt, fluid-present melts crystallised intermittently between 34 and 16 Ma and this was the dominant melt-producing reaction prior to ~ 25 Ma. After this, muscovite-dehydration melting became the dominant reaction type, coinciding with the overall increase in the volume of zircon and monazite. Biotite-dehydration melt signatures are only present in one leucogranite sample, which has a similar age profile to the Group 2 leucogranites. Drawing robust conclusions about this group is unwise without analysis of further samples.

Recent studies have shown that plutons in the Himalaya grew incrementally over long periods from multiple, distinct melt batches (Cottle et al. 2019; Harris et al. 2000; Lederer et al. 2013). Our migmatite and leucogranite age records support this model - some samples yield discrete age populations, suggesting short-lived geochronometer crystallisation, while others yield dates that spread over a significantly longer time period. Our geochemical and petrographic results from leucogranites and migmatites also demonstrate that at least two (maybe three) different melting reactions generated partial melts, as suggested by Cottle et al. (2019). Additionally, we can show both petrographically and geochronologically that the leucogranites were coeval with, and derived from, the regional migmatites in the GHS.

## Conclusions

Migmatites and leucogranites sampled near Badrinath, Garhwal Himalaya, India preserve petrographic and mineral chemistry indicators of different mica breakdown melting reactions that generated melts across protracted periods. Despite sharing similar thermal histories, the Garhwal samples fall predominately into two groups, recording either melting in the presence of free fluids or muscovite dehydration melting, but never both. A single leucogranite sample that fits into neither group shows evidence of biotite dehydration melting.

Our results reveal a complex interplay between bulk, mineral and fluid compositions and melting processes. Alongside textural evidence for different melt reactions, trace element abundances in constituent major minerals, especially Rb/Sr, Ba and Sn, of K-feldspar, plagioclase, muscovite and biotite, mirror trends previously reported in bulk rock signatures. However, our data cannot resolve whether this compositional variability is controlled by local bulk composition or the progression of different melt reactions. Geochronological data show that individual samples < 5 m away from each other record different melt reactions at the same time, as well as the same melt reactions at different times, depending on (1) subtle variations in fluid composition and availability and (2) bulk composition differences. The overall time-frame of melting and melt crystallisation lies between 35 − 13 Ma, with intermittent, localised melting before ~ 25 Ma and a significant pulse between 25 − 18 Ma. Sporadic fluid-present melting seems to have been triggered by local influxes of fluid, perhaps released from other partial melts crystallising nearby. Rocks that experienced fluid-present melting stopped reacting once muscovite had been consumed, meaning that muscovite dehydration melting only occurred in rocks untouched by fluids.

Our data show how petrography, geochemical composition, geothermometry and geochronology provide a powerful tool for identifying melt reactions and interrogating timing and timescales of reaction even in cases where the melt has left the source region (e.g. leucogranites), or where the progression of melting has (partially) overprinted petrographic evidence (e.g. diatexitic migmatites).

## Supplementary Information

Supplementary Material 1-3 Detailed method description & instrument metadata, Detailed sample description, Zircon and monazite spot analysis locations

Supplementary Material 2 Major element data minerals

Supplementary Material 3 Trace element data minerals

Supplementary Material 4 Zircon & Monazite datasets
