# Supplementary material for: Melt reactions and timescales of melting in pelitic rocks—a case study from the Garhwal Himalaya
Source: Contrib Mineral Petrol. 2025 Aug 22;180(9):62. doi: 10.1007/s00410-025-02247-z (PMC12370819; doi:10.1007/s00410-025-02247-z)

Online Resources 1

Melt reactions and timescales of melting in pelitic rocks – a case study from the Garhwal Himalaya

Contributions to Mineralogy and Petrology

Oldman, C. J.[1], Warren, C. J.[1], Harris, N.B.W.[1], Kunz, B.E.[1], Spencer, C. J.[2], Argles, T.W.[1], Roberts, N.M.W.[3], Hammond, S.J.[1], Degli Alessandrini, G.[1]

1. School of Environment, Earth and Ecosystems, Faculty of Science, Technology, Engineering and Mathematics, The Open University, Walton Hall, Milton Keynes, MK6 6AA, United Kingdom
2. Department of Geological Sciences and Geological Engineering, Queen's University, Kingston, Ontario KL7 3N6, Canada
3. Geochronology and Tracers Facility, British Geological Survey, Keyworth, Nottingham NG12 5GG, United Kingdom

Corresponding author: barbara.kunz@open.ac.uk

Detailed Analytical Methods

Major element compositions

Major element compositions of major phases were collected by point and line-analysis using a Zeiss Supra 55 VP field-emission SEM fitted with an Oxford Instruments X-Max 50 mm^2 EDS detector at the Open University. All analytical sessions were carried out between November 2021 and March 2022. The beam was set to a voltage of 20 keV and a current equivalent of 2.0 nA (60 µm aperture). Acquisitions were measured for 30 seconds of live-sensor time. EDS results are unnormalized and corrected for detector sensitivity loss using a natural almandine external standard, from the GEO MkII standard block made by P&H Developments ltd for EPMA analyses. This standard has a range of known major and minor

element oxide wt% values from 37.17 – 0.20 wt%, allowing for evaluation of precision and accuracy across a wide range of oxide concentrations. Confidence in the precision of values is better than 0.3 oxide wt% (2σ).

The EDS was calibrated using the cobalt in the Geo MkII standard block (P&H Developments: https://pandhdevelopments.com/geological.html) for ~200,000 counts (10-15 seconds). EDS acquisition settings were as follows:

- Number of Channels – 2048
- Process time – 4
- Acquisition Time (s) – 30.00

Major element maps

Whole-section EDS chemical maps were collected using a FEI Quanta 200 3D tungsten-filament SEM, fitted with an Oxford Instruments INCA EDS detector, at the Open University. The beam was set to a voltage of 20 keV and a current of 1.2 nA. EDS map sections were then stitched together using the Oxford Instruments INCA software 'Montage' function.

Trace element compositions

Trace element concentrations were collected by spot analysis using a Photon Machines Analyte G2 193 nm excimer laser system equipped with a HelEX II laser ablation cell coupled with an Agilent 8800 Triple Quadrupole ICP-MS at the Open University. The ICP-MS was tuned using NIST SRM 612 glass prior to each analytical session. Analyses were carried out between June 2021 and April 2022. Spot analyses were run using a fluence of 3.63 J/cm^2, a repetition rate of 10 Hz, and a spot size of 50 µm, with each analysis collecting 30 seconds of background, 30 seconds of sample ablation, and 40 seconds of post-ablation washout. Concentrations of 54 elements were collected, see detailed list in Table 1.

For feldspar analyses in samples 04b, 07c, and 09b a pre-ablation laser pulse was performed in order to improve laser-sample coupling, followed by a 10-second pause before collecting background measurements. Spots were analysed in batches of twenty unknowns, bracketed by two analyses of both 612 and BCR-2G standards. Analyses were typically collected on a one spot per grain basis, however, in samples with few grains of K-feldspar, or large porphyroblasts, multiple spots were collected within one grain.

Data reduction for spot analyses was processed using the Iolite v3.71 software package (Paton et al., 2011), with baseline signal subtraction and time-resolved spectra checked for inclusion signals and ablation issues. Only stable signal sections were used for concentration calculations. NIST SRM 612 was used as the primary standard and BCR-2G as the secondary standard, excluding Na, Mg, Al, P, K, Ca, Ti, Mn, and Fe, where BCR-2G was used as the primary standard due to higher concentrations of these elements. Si was used as the standardisation element with values were taken from EDS measurements (either directly from the analysis site, or from averages of similar grains for each mineral phase where direct measurement was not possible). Indium concentrations were corrected for ^{115}Sn isobaric interference with the following equation:

$In_{corrected}$ (ppm) = $In_{measured}$ (ppm) – (0.0034*Sn (ppm))

Accuracy and precision of BCR-2G analyses were compared to published values (Jenner and O'Neill, 2012) as well as in-house long-term averages and were found to be in good agreement, with relative standard deviations of 3-5 % for most elements.

Table 1: Metadata reporting for LA-ICP-MS

(adapted from http://www.plasmage.org/recommendations)

**Laboratory & Sample Preparation**	
Laboratory name	School of Environment, Earth and Ecosystem Sciences, Open University, UK
Sample type/mineral	biotite, muscovite, garnet, plagioclase, K-feldspar, tourmaline & sillimanite
Sample preparation	Thin section and grain mounts
**Laser ablation system**	
Make, Model & type	Photon Machines Analyte G2 193 nm excimer laser
Ablation cell & volume	HelEx II 2-volume cell
Laser wavelength (nm)	193 nm
Pulse width (ns)	4 ns
Fluence (J/cm^2)	3.63 J/cm^2
Repetition rate (Hz)	10 Hz
Ablation duration (s)	30 s
Spot diameter (mm)	50 µm
Sampling mode / pattern	static spots
Carrier gas	100% He in the cell, Ar make-up gas combined in a mixing bulb downstream.
Cell carrier gas flow (l/min)	0.9 l/min He
**ICP-MS Instrument**	
Make, Model & type	Agilent 8800 ICP-QQQ-MS
Sample introduction	Ablation aerosol in He & Ar gas mix
RF power (W)	1250 W
Ar carrier gas flow (l/min)	0.77-0.79 l/min

Detection system	Dual-mode discrete dynode electron multiplier
Masses measured; Integration time per peak/dwell times (ms)	^{7}Li 0.005; ^{9}Be 0.005; ^{23}Na 0.002; ^{24}Mg 0.005; ^{27}Al 0.002; ^{29}Si 0.002; ^{31}P 0.01; ^{39}K 0.005; ^{43}Ca 0.005; ^{45}Sc 0.005; ^{49}Ti 0.005; ^{51}V 0.005; ^{53}Cr 0.005; ^{55}Mn 0.005; ^{56}Fe 0.005; ^{59}Co 0.005; ^{60}Ni 0.005; ^{65}Cu 0.005; ^{66}Zn 0.005; ^{71}Ga 0.005; ^{74}Ge 0.005; ^{85}Rb 0.005; ^{88}Sr 0.005; ^{89}Y 0.005; ^{90}Zr 0.005; ^{93}Nb 0.01; ^{95}Mo 0.01; ^{111}Cd 0.01; ^{115}In 0.01; ^{118}Sn 0.01; ^{121}Sb 0.02; ^{133}Cs 0.02; ^{137}Ba 0.01; ^{139}La 0.01; ^{140}Ce 0.01; ^{141}Pr 0.01; ^{146}Nd 0.01; ^{147}Sm 0.01; ^{153}Eu 0.01; ^{157}Gd 0.01; ^{159}Tb 0.01; ^{163}Dy 0.01; ^{165}Ho 0.01; ^{166}Er 0.01; ^{169}Tm 0.01; ^{172}Yb 0.01; ^{175}Lu 0.01; ^{177}Hf 0.01; ^{181}Ta 0.01; ^{182}W 0.02; ^{205}Tl 0.02; ^{208}Pb 0.02; ^{209}Bi 0.02; ^{232}Th 0.01; ^{238}U 0.01
Total integration time (s)	0.6504
Gas blank	30 seconds
Washout	40 seconds
**Data Processing**	
Calibration strategy	SRM-NIST 612 (primary for trace elements), BCR-2G (secondary for traces, primary for majors) every ~20 analyses
Reference Material info	SRM-NIST 612 (Jenner and O'Neill, 2012)<br>BCR-2G (Jenner and O'Neill, 2012 & in house long-term averages)
Data processing package used	Iolite v3.71; DRS: X_Trace_Elements_IS; internal standard ^{29}Si
Uncertainty level & Quality control / Validation	BCR-2G within 3-5% of the preferred value depending on element concentration and homogeneity of secondary standard.<br>Major element concentrations obtained by LA-ICP-MS were compared to those measured by EMPA for consistency checks and data defines on a 1:1 line

U-(Th)-Pb isotopes in zircon and monazite

Samples were crushed then separated following standard procedures including passing across a Wilfley table, passing through a Frantz magnetic separator and finally passed through sodium polytungstate (heavy liquid) solution at the University of Portsmouth. Zircon and monazite were picked from the resulting heavy mineral fraction and mounted in epoxy resin.

U-Pb isotopes were measured in zircon rims using a Resonetics RESOltuion M-50A-LR incorporating a Compex 102 excimer laser together with an Agilent 7700s quadrupole ICP-MS at Curtin University, Australia. Spot analyses were performed using a fluence of 1.7 J/cm^2, a repetition rate of 7 Hz, and a spot size of 23 µm. Samples were bracketed by reference material analyses every 22 unknowns. Zircon 91500 (1062.4 ± 0.4 Ma; Wiedenbeck et al., 1995) was used as a primary standard. Plešovice (337.13 ± 0.37 Ma; Sláma et al., 2008), GJ-1 (601.92 ± 0.7 Ma; Jackson et al., 2004), and OG1 (3465.4 ± 0.6 Ma; Stern et al., 2009) were monitored as secondary standards. 91500 yielded a ^{206}Pb/^{238}U weighted average age of 1062.6 ± 2.4 Ma (MSWD = 0.63, n = 20), Plešovice yielded a ^{206}Pb/^{238}U weighted average age of 341.8 ± 0.5 Ma (MSWD = 2.1, n = 19), GJ-1 yielded a ^{206}Pb/^{238}U weighted average age of 616.3 ± 1.0 Ma (MSWD = 1.2, n = 20), and OG1 yielded a ^{206}Pb/^{238}U weighted average age 3491 ± 1.7 Ma (MSWD = 11, n = 20). Calculated average ages and uncertainties of the secondary standards overlap within 2.4 % of the reference values. Time-resolved mass spectra were reduced in Iolite (Paton et al., 2011). Zircon ^{206}Pb/^{238}U dates were corrected using the Stacey-Kramers two-stage isotope evolution model (Stacey and Kramers, 1975). Full zircon U-(Th)-Pb analysis standard measurements are included in Online Resource 6.

U-Th-Pb isotopes in monazite were measured using an ESI/NWR193UC laser with a TV2 ablation cell coupled to a Nu Instruments Attom SC-ICP-MS at the Geochronology and Tracers Facility, British Geological Survey, UK. Material was ablated using a laser fluence of 3 J/cm^2, a repetition rate of 10 Hz, and a spot size of ~11 µm. Monazite standard Bananeira (507.7 ± 1.3 Ma; Gonçalves et al., 2016) was used as the primary reference. Monazites 44069 (424.9 ± 0.4 Ma; Aleinikoff et al., 2006) and FC1 (55.7 ± 0.7 Ma; Horstwood et al., 2003) were used as the secondary standards.

In session ages for 44069 yielded an average age of 430.7 ± 4.7 Ma (MSWD = 1.9, n = 32), and FC1 yielded an average age of 53.89 ± 0.62 Ma (MSWD = 1.8, n= 37). Calculated average ages and uncertainties of the secondary standards overlap within 4.65 % of the preferred values. Data were reduced using the mean of the ratios calculated in the Attolab time-resolved analysis software. No downhole correction was applied and fractionation was assumed to be similar between standards and samples. To avoid inaccurate normalisation, the mean of the ablation period (excluding the initial 2-3 seconds to allow for Pb contamination on the surface) was used for both standards and samples. Reported monazite ^{208}Pb/^{232}Th dates were corrected using the Stacey-Kramers two-stage isotope evolution model (Stacey and Kramers, 1975). For full monazite U-Th-Pb analysis standard measurements, see Online Resources 6.

Trace element analyses in accessory phases

Zircon trace element compositions were measured in zircon rims over the U-Pb ablation pits on an Agilent 7700s quadrupole ICP-MS at Curtin University, Australia. Samples were ablated using a 33 µm spot size and the same laser conditions as for the U-Pb analysis and concentrations of Ti, Y, Zr, Nb, La, Ce, Pr, Nd, Sm, Eu, Gd, Dy, Yb, Lu, Hf, Th, and U were collected. Reference material analyses were performed every 22 unknowns. NIST SRM 610 glass was used as the primary standard and NIST SRM 612 as the secondary standard. Si was used as the internal standardisation element. Accuracy and precision of NIST SRM 612 analyses were accessed using published values (Gao et al., 2002) and were found to be in good agreement (12-20 %), see Online Resource 6.

Table 2: Metadata reporting for LA-ICP-MS monazite geochronology

**Laboratory & Sample Preparation**	
Laboratory name	Geochronology and Tracers Facility (GTF) British Geological Survey
Sample type/mineral	Monazite
Sample preparation	Conventional mineral separation, 1 inch resin mount, 0.25 mm polish to finish
Imaging	EDS maps
**Laser ablation system**	
Make, Model & type	ESI/New Wave Research, 193UC
Ablation cell & volume	TV2
Laser wavelength (nm)	193 nm
Pulse width (ns)	4 ns

Fluence ($J.cm^{-2}$)	3
Repetition rate (Hz)	10
Ablation duration (secs)	15
Ablation pit depth / ablation rate	ca. equivalent to 0.08mm/pulse
Spot diameter (mm) nominal/actual	11
Sampling mode / pattern	Static spot ablation
Carrier gas	100% He in the cell, Ar make-up gas combined using a Y-piece 50% along the sample transport line to the torch.
Cell carrier gas flow (l/min)	0.6 l/min
**ICP-MS Instrument**	
Make, Model & type	Nu Instruments, Attom, SC-ICP-MS
Sample introduction	Ablation aerosol

RF power (W)	1300W
Make-up gas flow (l/min)	Sourced from Nu Instruments DSN-100 desolvating nebulizer. Neb pressure 24psi (estimated at 0.7l/min) Ar.
Detection system	single Mascom Secondary Electron Multiplier
Masses measured	163, 172, 206, 207, 208, 232, 235, 238
Integration time per peak/dwell times (ms); quadrupole settling time between mass jumps	200 µs for 163 and 172; 400 µs for 206; 600 µs for 232; 800 µs for 207 and 235; 1200 µs for 208
Total integration time per output datapoint (secs)	0.2 secs<br>*(N.B. this should represent the time resolution of the data)*
'Sensitivity' as useful yield (%, element)	ca. 0.15% U<br>*((#ions detected/#atoms sampled)*100; Schaltegger et al. 2015)*
IC Dead time (ns)	11 ns
**Data Processing**	
Gas blank	60 second on-peak zero subtracted

Calibration strategy	Bananeira used as primary for Pb/U and Th/Pb ratios, NIST610 for Pb/Pb ratios.
Reference Material info	Bananeira (Gonçalves et al., 2016)<br><br>44069 (Aleinikoff et al., 2006)<br><br>FC1 (Horstwood et al., 2003)
Data processing package used / Correction for LIEF	Nu Instruments Nu Attolab TRA software and in-house spreadsheet for data normalization, uncertainty propagation and age calculation. LIEF correction assumes reference material and samples behave identically.
Mass discrimination	sample standard bracketing
Common-Pb correction, composition and uncertainty	^{207}Pb based common-Pb correction using assumed Stacey and Kramers (1975) values and assuming concordance.
Uncertainty level & propagation	Ages are quoted at 2s absolute, propagation is by quadratic addition. Reproducibility and age uncertainty of reference material and common-Pb composition uncertainty are propagated where appropriate.
Quality control / Validation	44069 Ref Age: 424.9 +/- 0.4 Ma; Measured in the session: 430.7 +/- 4.7 Ma (MSWD = 1.9)

	FC1 Ref Age 55.7 +/- 0.7 Ma; Measured in the session 53.89 +/- 0.62 Ma (MSWD = 1.8)

References

Aleinikoff JN, Schenck WS, Plank MO, Srogi L, Fanning CM, Kamo SL, Bosbyshell H (2006) Deciphering igneous and metamorphic events in high-grade rocks of the Wilmington Complex, Delaware: Morphology, cathodoluminescence and backscattered electron zoning, and SHRIMP U-Pb geochronology of zircon and monazite. Geological Society of America Bulletin 118:39-64. https://doi.org/10.1130/B25659.1

Gao S, Liu X, Yuan H, Hattendorf B, Günther D, Chen L, Hu S (2002) Determination of forty two major and trace elements in USGS and NIST SRM glasses by laser ablation-inductively coupled plasma-mass spectrometry. Geostandards Newsletter, 26:181-196. https://doi.org/10.1111/j.1751-908X.2002.tb00886.x

Gonçalves GO, Lana C, Scholz R, Buick IS, Gerdes A, Kamo SL, Corfu F, Marinho MM, Chaves AO, Valeriano C, Nalini Jr HA (2016) An assessment of monazite from the Itambé pegmatite district for use as U–Pb isotope reference material for microanalysis and implications for the origin of the "Moacyr" monazite. Chemical Geology 424:30-50. https://doi.org/10.1016/j.chemgeo.2015.12.0.

Horstwood MS, Foster GL, Parrish RR, Noble SR, Nowell GM (2003) Common-Pb corrected in situ U–Pb accessory mineral geochronology by LA-MC-ICP-MS. Journal of Analytical Atomic Spectrometry 18:837-846. https://doi.org/10.1039/B304365G.

Jackson SE, Pearson NJ, Griffin WL, Belousova EA (2004) The application of laser ablation-inductively coupled plasma-mass spectrometry to in situ U–Pb zircon geochronology. Chemical Geology 211:47–69. https://doi.org/10.1016/j.chemgeo.2004.06.017.

Jenner FE, O'Neill HSC (2012) Major and trace analysis of basaltic glasses by laser-ablation ICP-MS. Geochemistry Geophysics Geosystems 13:Q03003. https://doi.org/10.1029/2011GC004009.

Paton C, Hellstrom J, Paul B, Woodhead J, Hergt J (2011) Iolite: Freeware for the visualisation and processing of mass spectrometric data. Journal of Analytical Atomic Spectrometry 26:2508-2518. https://doi.org/10.1039/C1JA10172B.

Schaltegger U, Schmitt AK, Horstwood MSA (2015) U–Th–Pb zircon geochronology by ID-TIMS, SIMS, and laser ablation ICP-MS: Recipes, interpretations, and opportunities. Chemical Geology 402: 89-110. https://doi.org/10.1016/j.chemgeo.2015.02.028

Sláma J, Košler J, Condon DJ, Crowley JL, Gerdes A, Hanchar JM, Horstwood MSA, Morris GA, Nasdala L, Norberg N, Schaltegger U, Schoene B, Tubrett MN, Whitehouse MJ (2008) Plešovice zircon — A new natural reference material for U–Pb and Hf isotopic microanalysis. Chemical Geology 249:1–35. https://doi.org/10.1016/j.chemgeo.2007.11.005.

Stacey JS, Kramers JD (1975) Approximation of terrestrial lead isotope evolution by a two-stage model. Earth and Planetary Science Letters 26:207–221. https://doi.org/10.1016/0012-821X(75)90088-6.

Stern RA, Bodorkos S, Kamo SL, Hickman AH, Corfu F (2009) Measurement of SIMS Instrumental Mass Fractionation of Pb Isotopes During Zircon Dating. Geostandards and Geoanalytical Research 33:145–168. https://doi.org/10.1111/j.1751-908X.2009.00023.x.

Wiedenbeck M, Allé P, Corfu F, Griffin Wl, Meier M, Oberli F, Quadt AV, Roddick J, Spiegel W (1995) Three Natural Zircon Standards for U-Th-Pb, Lu-Hf, Trace Element and Ree Analyses. Geostandards Newsletter 19:1–23. https://doi.org/10.1111/j.1751-908X.1995.tb00147.x.

Online Resources 2

Sample information

2.1: Sample locality data

2.2. Mineralogical summary data

2.3: Field relations and sample descriptions

2.1: Sample locality data

Table S1 – Samples collected from the Garhwal Himalaya, India. The letter 'f' in sample ID indicates collection as a float sample. Coordinates are given in decimal degrees.

Sample ID	Location	Group	Latitude	Longitude	Rock type	Comments
**BAD01d**	Badrinath - Hanuman	2	30.71693	79.49751	Leucogranite	Medium-grained in-source Grt-leucogranite. Grt & Bt form thin layering in line with host migmatite schlieren and schollen/rafts.
**BAD02a**	Rishi Ganga	2	30.74428	79.47037	Migmatite	Gneissose Tur-metatexite with disseminated 0.5 cm scale leucosomes. Leucosomes up to 20 x 10 cm.
**BAD02b**	Rishi Ganga	2	30.74428	79.47037	Migmatite leucosome	Leucosome pod within metatexite. Bt-rich schlieren sub-parallel with host, more prevalent towards the leucosome boundaries.
**BAD03**	Rishi Ganga	2	30.74440	79.46177	Leucogranite	Medium-grained Bt-leucogranite. The boundary between the leucogranite and host-migmatite is sharp.
**BAD04a**	Rishi Ganga	2	30.74426	79.45016	Migmatite	Schlieren diatexite with rafts. Sil-Bt selvedges are common along schollen/raft-leucosome transitions.

**BAD04b**	Rishi Ganga	2	30.74426	79.45016	Migmatite	Schollen diatexite with schlieren. Sil-Bt selvedges are common along schollen/raft-leucosome transitions.
**BAD05b**	Mana - Alaknanda	1	30.77444	79.48951	Migmatite	Medium-grained melanocratic migmatite. Thin (1-2 cm) leucosome layers. Considered to be melt-extracted residuum.
**BAD06**	Mana - Alaknanda	3	30.77517	79.48624	Leucogranite	Medium-grained Grt-Tur-leucogranite. Tur forms sub-parallel horizons in association with Qz.
**BAD07a**	Mana - Alaknanda	2	30.78055	79.47762	Migmatite	Schlieren diatexite. Sil-Bt selvedges line leucosome boundaries. Associated with 1-5 cm wide vein-structured leucocratic
**BAD07b**	Mana - Alaknanda	2	30.78055	79.47762	Leucogranite	Medium-grained Tur-leucogranitic dyke, 2.5-3.0 m wide. Associated dykelet network cross-cutting host migmatite, up to
**BAD07c**	Mana - Alaknanda	1	30.78055	79.47762	Migmatite	Deformed melanocratic diatexite. Irregular leucosomes and Sil-Bt-rich selvedges.
**BAD08**	Hanuman Chatti	1	30.69918	79.50424	Migmatite/ Schist	Fine-grained schistose migmatite with folded vein-like leucosomes 1-4 mm wide in hand specimen (up to 5 cm wide at
**BAD09b**	Hanuman Chatti	1	30.70085	79.50388	Migmatite/ Schist	Medium-grained schistose migmatite with vein-like leucosomes 1-8 mm wide.
**BAD11a**	Joshimath	1	30.56328	79.56776	Migmatite	Deformed Bt-Grt-Tur metatexite. Folded, irregular leucosomes 3-8 mm wide.
**BADf02**	Rishi Ganga	2	30.74142	79.47026	Migmatite	Schlieren diatexite with coarse-grained Tur-rich leucosome. Sil-Ms clots along the boundary between leucogranite and
**BADf04**	Rishi Ganga	2	30.74142	79.47026	Leucogranite	Tur-Bt-leucogranite, coarse-grained Qz + Fsp, with medium-grained micaceous areas.
**BADf13**	Rishi Ganga	2	30.74233	79.45197	Leucogranite/ Migmatite	Schollen diatexite paired with very coarse Tur-leucogranite. Boundary lined with Tur & Qz.

2.2. Mineralogical summary data

Table S2 – Mineralogical summary of key samples. Key: x = present, - = absent, ~ = former presence (of sil) inferred from muscovite-sericite clots. Where present, chlorite is interpreted as retrograde. Mineral abbreviations from Whitney and Evans 2011.

Sample	Rock type	Qz	Pl	K-fsp	Bt	Ms	Gt	Sil	(Chl)	Tm	Ilm	Rt	Zir	Ap	Mnz
BAD01d	Leucogranite	x	x	x	x	x	x	~	x	-	-	x	x	x	x
BAD02a	Migmatite	x	x	x	x	x	x	x	x	x	-	x	x	x	x
BAD02b	Migmatite/ Leucosome	x	x	x	x	x	x	-	x	-	-	x	x	x	x
BAD03	Leucogranite	x	x	x	x	-	-	-	x	-	-	x	x	x	-
BAD04a	Migmatite	x	x	x	x	x	-	x	x	x	-	x	x	x	x
BAD04b	Migmatite	x	x	x	x	x	-	x	x	x	-	x	x	x	x
BAD05b	Migmatite	x	x	x	x	x	-	x	x	x	x	-	x	x	x
BAD06	Leucogranite	x	x	x	-	x	x	-		x	-	-	x	-	x
BAD07a	Migmatite	x	x	x	x	x	-	x	x	-	-	x	x	x	-
BAD07b	Leucogranite	x	x	x	x	x	-	x	x	x	-	x	x	x	x
BAD07c	Migmatite	x	x	x	x	x	x	x	x	-	-	x	x	x	x
BAD08	Migmatite	x	x	x	x	x	x	~	x	x	x	-	x	x	x
BAD09b	Migmatite	x	x	-	x	x	x	-	x	-	x	-	x	-	x
BAD11a	Migmatite	x	x	-	x	x	x	~		x	x	-	x	x	x
BADf02	Migmatite	x	x	x	x	x	x	~		x	-	x	x	x	x
BADf03	Orthogneiss	x	x	x	x	x	x	x	-	-	x	-	x	x	x
BADf04	Leucogranite	x	x	x	x	x	-	x	x	x	-	x	x	x	x
BADf13	Migmatite	x	x	x	x	x	-	~	x	x	x	-	x	x	x

2. 3: Field relations and sample descriptions

All samples with an "*f*" precursor were collected from float; all other samples were collected *in-situ*.

Locality 01

Sampling locality 01 is approximately 4 km south of Badrinath along NH7, where the cliff and highway extend west into the valley (30.71693°N, 79.49751°E). This locality lies wholly within the Badrinath Formation. Samples were retrieved *in situ* from the cliff face along the eastern side of the Alaknanda valley, adjacent to the NH7 highway. The outcrop along this section comprises leucosome-rich migmatites with schollen and schlieren, as well as metre-scale leucogranite bodies (Figure S1.2a). Boundaries between the melt and schollen are either gradational or lined with dark selvedges. Schlieren form closed folds within the melt (Figure S1.2b).

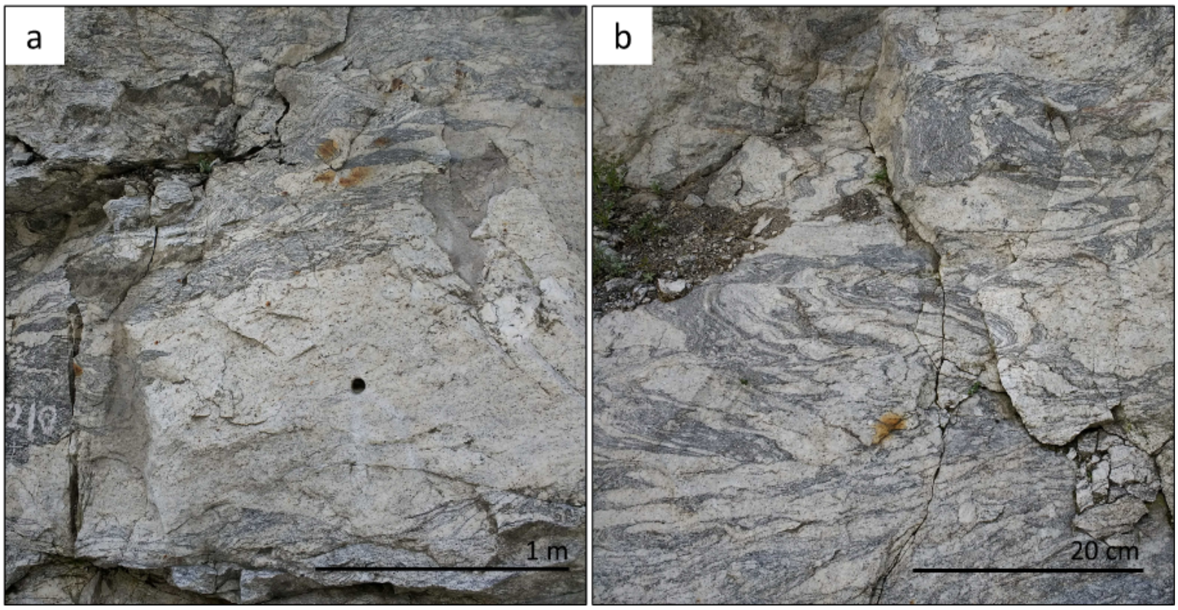

Figure S1.2 – Locality 01 (30.71693°N, 79.49751°E). a) Sampled leucogranite body within migmatite complex. b) Detail of melanocratic schlieren in migmatite surrounding the leucogranite.

Sample 01d is a medium-grained leucogranite collected *in-situ* from a 3 m wide granite body within a large migmatite complex of schollen and schlieren migmatites. It contains K-feldspar + quartz + plagioclase + garnet + muscovite + biotite, with apatite, zircon, and monazite present as accessory phases. Biotite is partially chloritized, with ~20% of grains affected. A weak planar fabric is present, with minerals generally elongated and aligned (Figure S1.3). Quartz grains show undulose extinction throughout the sample.
K-feldspar grains range between 0.8-3.5 mm across, the larger of which have perthitic albite exsolution lamellae, with irregular inclusions of quartz and plagioclase (Figure

S1.4a). Plagioclase grains are 0.8-3.6 mm across and show partial sericitization. Myrmekitic plagioclase is common along K-feldspar-plagioclase grain boundaries.

Biotite grains have complex irregular boundaries with adjacent feldspars and quartz. Some biotite grains have sagenitic rutile growth, with the most affected grains also being extensively chloritized (Figure S1.4b). Muscovite is either present as grains 0.8-2.0 mm in length or as fine-grained sericite schlieren up to 6 mm long and 350 μm wide (Figure S1.4c). These sericite clots are associated with K-feldspar and fine-grained biotite. Garnets are highly elongated, one grain reaching 3.7 by 1.0 mm, with fractures perpendicular to their long axes that have partially annealed or show biotite/chlorite alteration (Figure S1.4d). Small inclusions of <50 μm long, run along the central lengths of the garnets. Also present are inclusions of irregular lobate quartz.

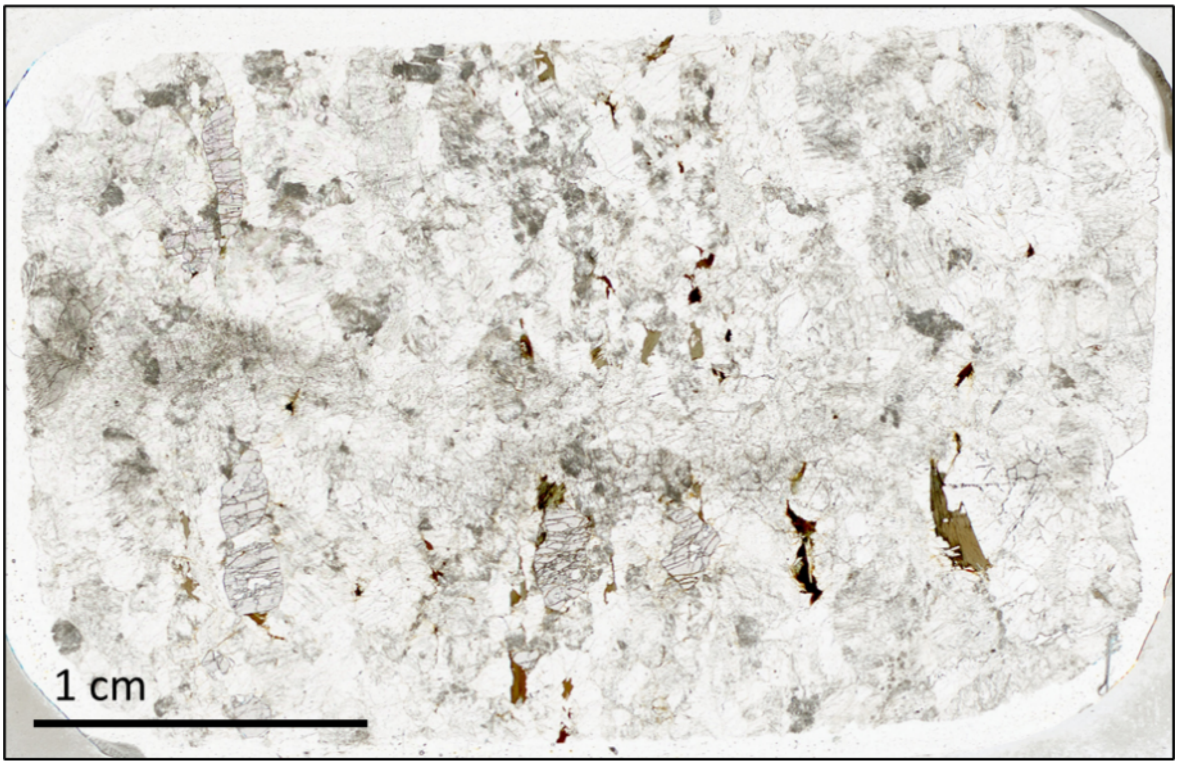

Figure S1.3 – Thin section image of leucogranite sample 01d, displaying fabric of aligned biotite schlieren and elongated garnet.

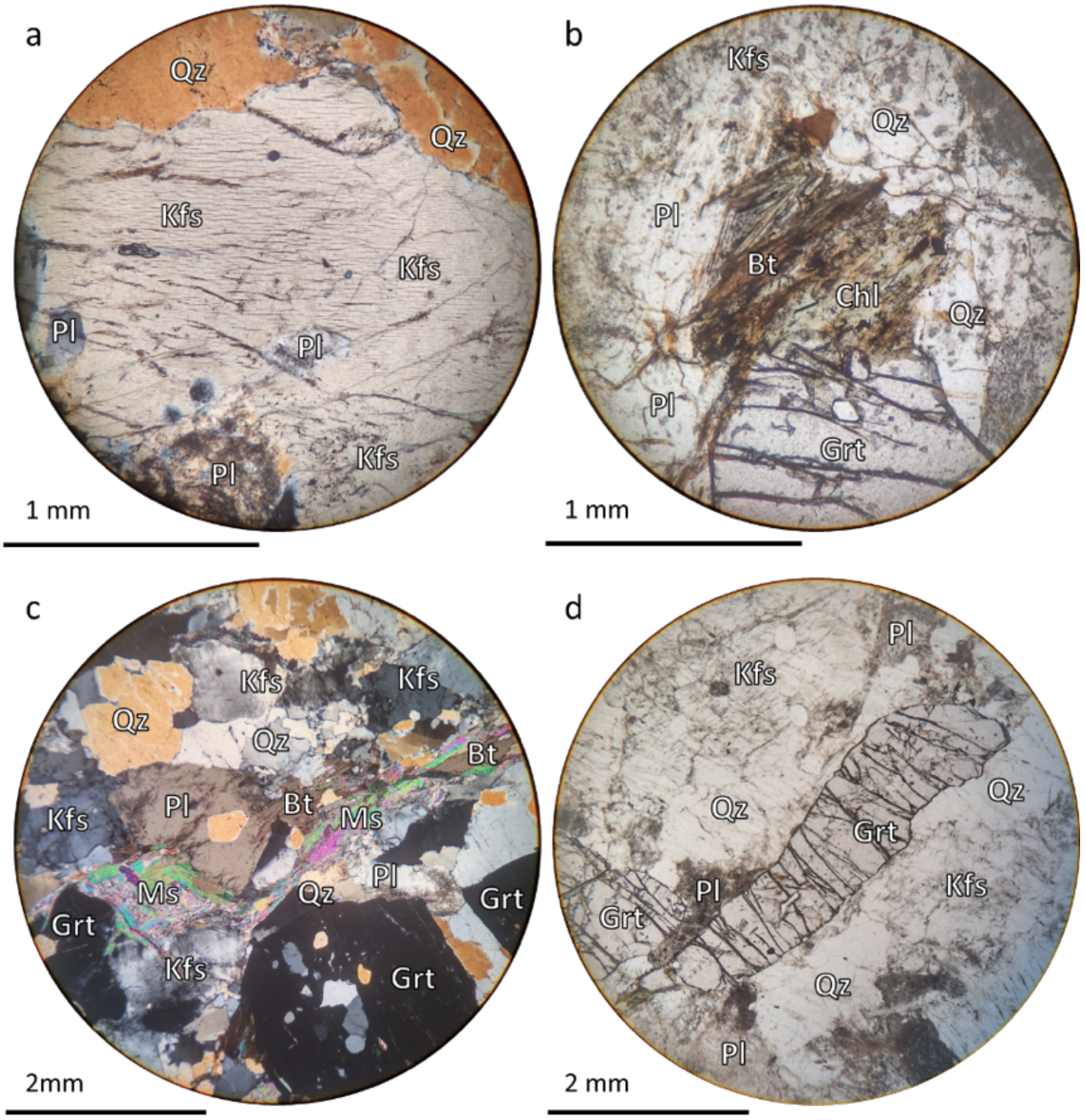

Figure S1.4 – Photomicrographs of various mineral textures in sample 01d. a) Coarse K-feldspar grain with perthitic albite exsolution lamellae. b) Partially chloritized biotite grain with sagenitic rutile growth. c) Muscovite-sericite schliere and clot. d) Highly elongated garnet grain with length-perpendicular fractures.

Locality 02

Locality 02 is 1.8 km west of Badrinath (30.74428°N, 79.47037°E). The sampled exposure consists of gneissose metatexite with 0.5-1 cm lenticular leucosomes throughout, as well as leucosome pods up to 20 cm across (Figure S1.5). The edges of these pods are lined with biotite schlieren.

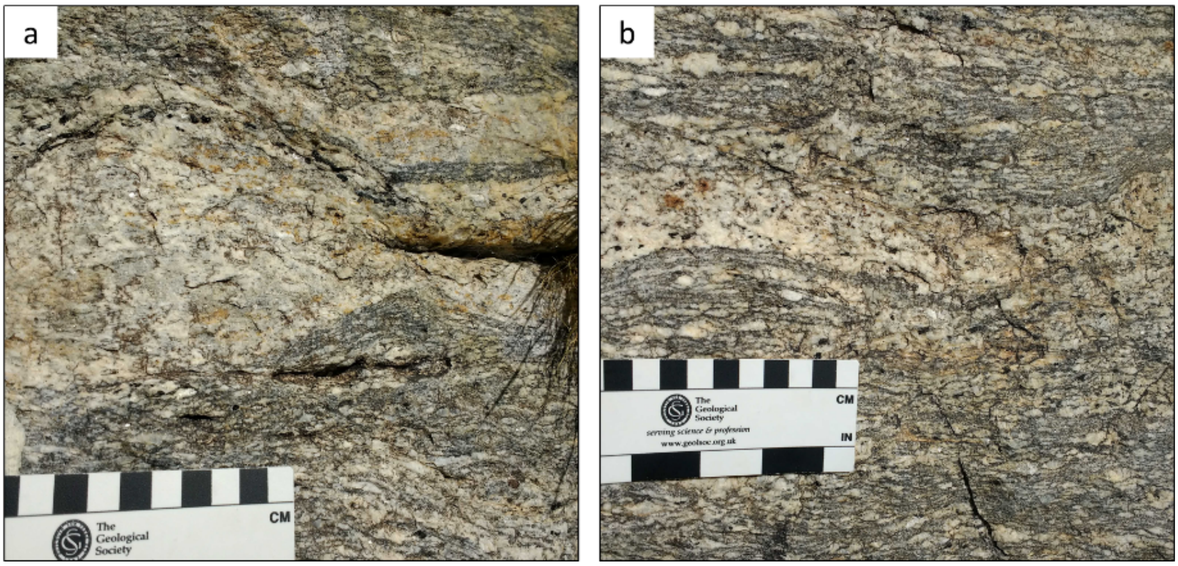

Figure S1.5 – Locality 02 (30.74428°N, 79.47037°E). a) Leucosome pod with tourmaline schliere, within migmatitic gneiss. b) Lenticular leucosome within migmatitic gneiss.

Sample 02a is a fine-grained metatexite with a gneissose fabric and medium-grained leucosomes (Figure S1.6). It consists of quartz + K-feldspar + plagioclase + biotite + sillimanite + muscovite + tourmaline, with apatite, zircon, and monazite present as accessory phases. Biotite grains within or adjacent to leucosomes are extensively chloritized. Quartz grains show undulose extinction throughout.

In the leucosomes, K-feldspar occurs as perthitic subhedral grains, 2-4 mm across, with rare grains > 5 mm. In the groundmass, K-feldspar forms sparse fine-grained aggregates around quartz, plagioclase, and biotite. Plagioclase shows albite twinning and partial sericitization, while grains adjacent to K-feldspar in leucosomes form myrmekites (Figure S1.7).

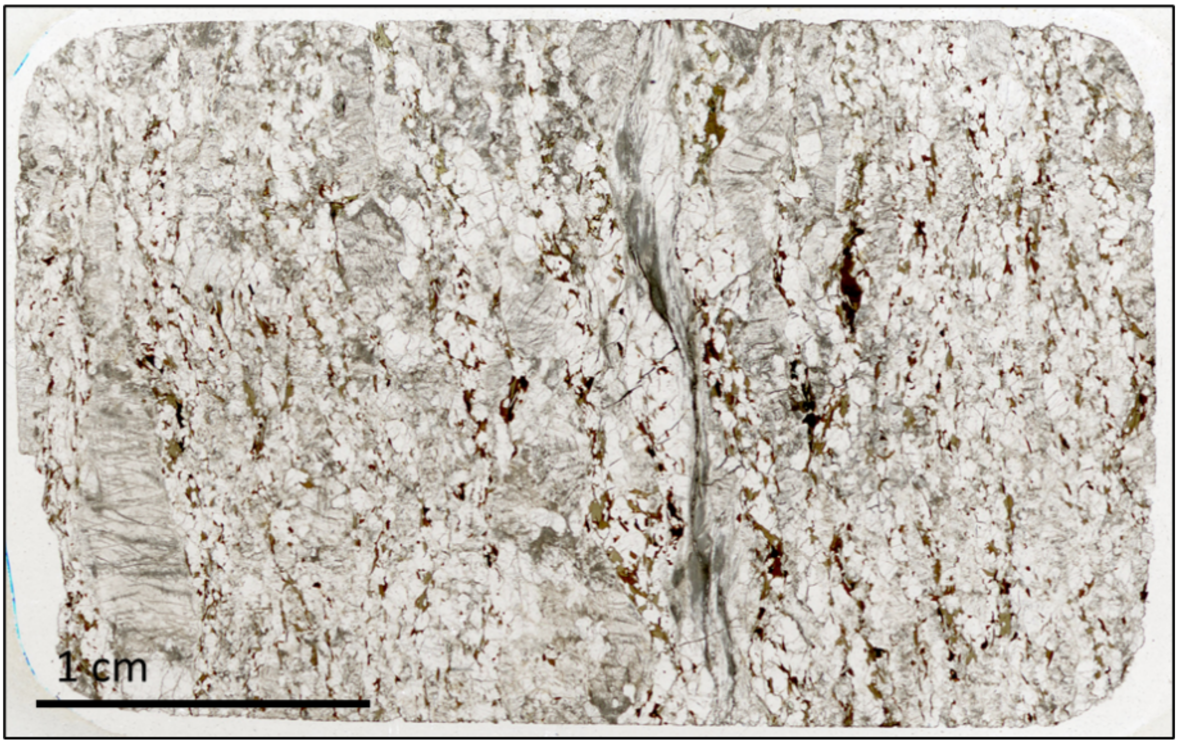

Figure S1.6 – Thin section image of migmatite sample 02a, displaying elongate fibrolite clot (centre, top to bottom) and rare K-feldspar porphyroblasts >5mm (bottom left).

Fine-grained biotite and muscovite form disaggregated phyllosilicate bands. Biotite also occurs sporadically throughout quartzofeldspathic domains, and as sagenitic chlorite. Sillimanite forms fibrolite clots up to 18 mm long and 2 mm wide, with fine-grained K-felspar, quartz and sericite intergrowths (Figure S1.7). Garnet is rare, with subhedral grains 0.8 mm in diameter associated with biotite and chlorite. Tourmaline is also rare, forming 0.75-1.25 mm grains adjacent to leucosomes, with strong olive-green to colourless pleochroism.

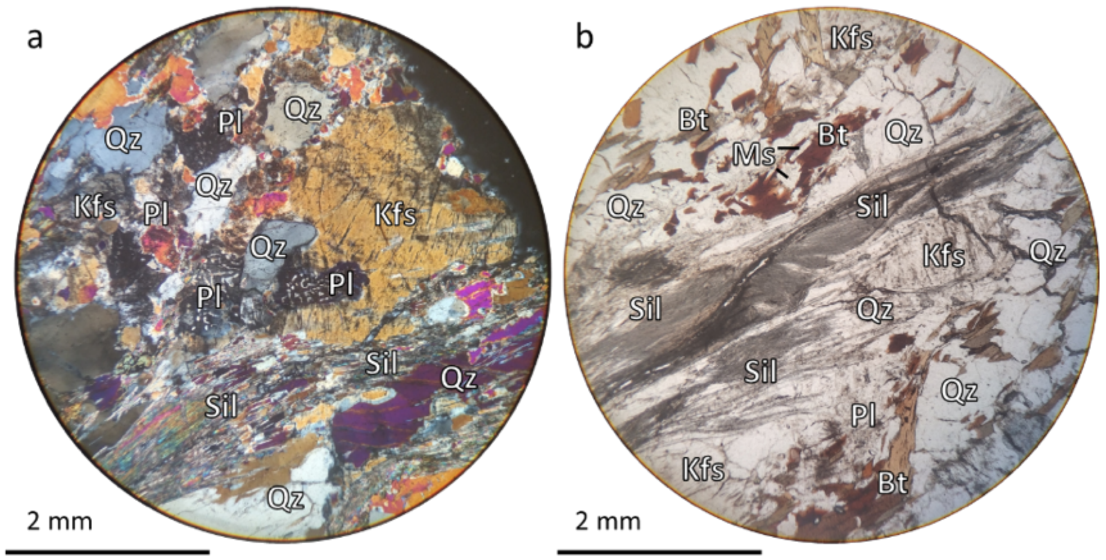

Figure S1.7 – Photomicrographs of feldspar and sillimanite textures in sample 02a. a) Medium-grain myrmekitic plagioclase and coarse-grains K-feldspar (XPL). b) Fibrolitic sillimanite with K-feldspar and quartz (PPL).

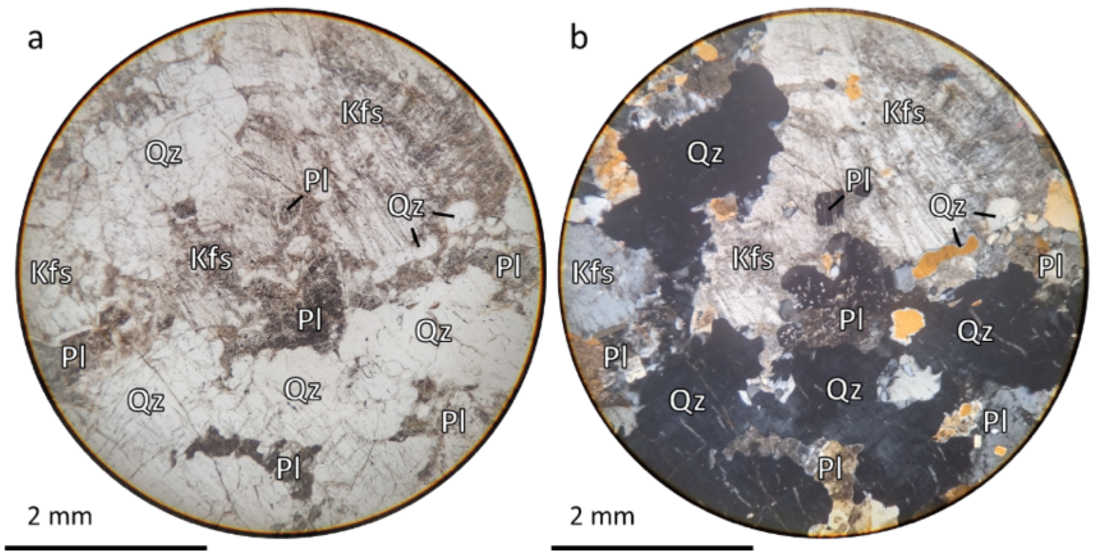

Figure S1.8 – Photomicrographs of feldspar textures in sample 02b. Medium-grained myrmekitic plagioclase adjacent to coarse perthitic K-feldspar. Plagioclase is strongly sericitized, while K-feldspar is preferentially sericitized along albite exsolution lamellae. Imaged in a) PPL, and b) XPL.

Sample 02b is an *in-situ* medium-grained migmatite leucosome sampled from one of the larger leucosome pods of locality 02. It is comprised of quartz + plagioclase + K-feldspar + chlorite/biotite + garnet + muscovite, with apatite, zircon, and monazite present as accessory phases. The leucosome is faintly foliated with bands of K-feldspar and phyllosilicate schlieren. Biotite is extensively chloritized, with few unaltered grains remaining. Quartz grains show undulose extinction throughout the sample.

K-feldspar forms perthitic grains with albite exsolution lamellae, typically 1-2 mm, and is partially sericitized. Some grains are poikiloblastic, with irregular quartz and plagioclase inclusions. Crosshatch twinning is present in smaller grains. Plagioclase forms 1.0-1.6 mm grains which are strongly sericitized. Myrmekitic plagioclase is common along boundaries with K-feldspar (Figure S1.8).

Chloritized biotite forms thin schlieren with small relict grains of muscovite. Chlorite is often sagenitic with fine rutile needles. Garnets are subhedral, 0.4-1.8 mm in diameter, with fractures lined with biotite and chlorite.

Locality 03

Sampling locality 03 is 0.8 km west of locality 02, 2.6 km west of Badrinath (30.74440°N, 79.46177°E). The exposure is a 3.4 x 1.5 m lenticular leucogranite body with a sharp upper contact with the host migmatite (Figure S1.9). The lower contact is obscured by sediment and scree.

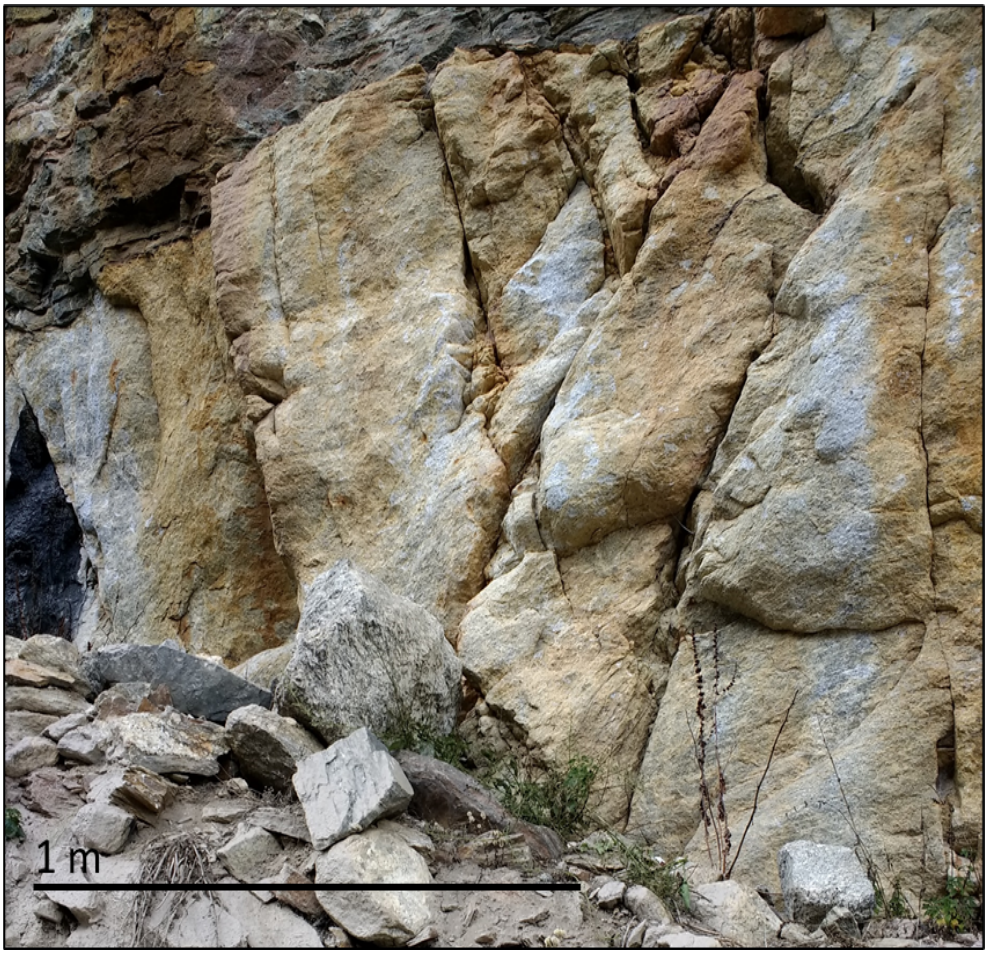

Figure S1.9 – Locality 03 (30.74440°N, 79.46177°E). Sampled lenticular leucogranite body, >3 m long, with sharp intrusive contact with host migmatite visible along its top edge.

Sample 03 is an *in-situ* medium-grained leucogranite taken from the leucogranite body from locality 03. It contains K-feldspar + plagioclase + quartz + biotite/chlorite + apatite,

with zircon and allanite present as accessory phases. Biotite is partially chloritized, with ~40% of grains affected. Quartz grains show undulose extinction throughout the sample.

K-feldspar forms perthitic grains, typically 1.6-4.6 mm across (up to 7 mm), with albite exsolution blebs and lamellae. Some grains are poikiloblastic, with irregular plagioclase, quartz, and chlorite inclusions. Crosshatch twinning is present in smaller grains. Partial sericitization is prevalent around exsolution textures. Plagioclase forms 1.3-4.5 mm grains which are strongly sericitized. Boundaries with K-feldspar are irregular, with myrmekitic plagioclase and quartz.

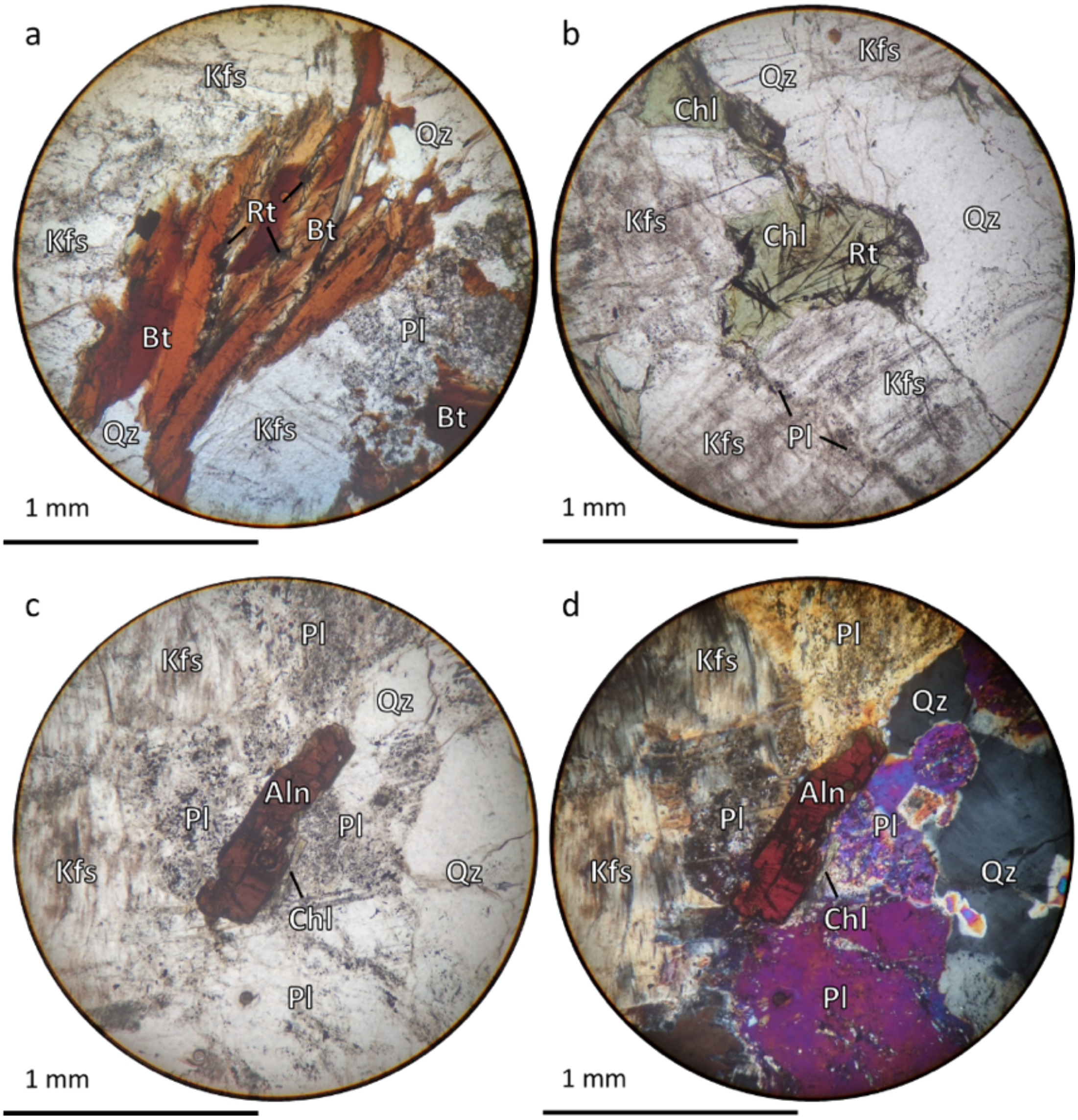

Figure S1.10 – Photomicrographs of various mineral textures in sample 03. a) Biotite laths with sagenitic rutile (PPL). b) Chlorite with sagenitic rutile (PPL). c) Rare allanite grain, 0.8 mm long (PPL). d) Rare allanite grain (XPL).

Biotite and chlorite form fine-grained laths, 0.3-0.8 mm long, with irregular grain boundaries. Sagenitic rutile is common in both minerals (Figure S1.10 a, b). Apatite

makes up approximately 1% of the rock by volume, forming euhedral hexagonal and rounded grains 0.25-1.3 mm in diameter. Biotite, chlorite, and apatite often form clusters. Accessory allanite is rare, with grains up to 0.8 mm in length (Figure S1.10 c, d).

Locality 04

Sampling locality 04 is 1.2 km west of locality 03, 3.8 km west of Badrinath (30.74426°N, 79.45016°E). The outcrop consists of transitional migmatites with varying proportions of leucosome to mesosome/schollen. Boundaries between rafts and melt are diffuse with schlieren and melanocratic selvedges.

Sample 04a is a medium-grained *in-situ* schlieren diatexite, consisting of K-feldspar + plagioclase + quartz + muscovite + biotite + sillimanite + tourmaline, with apatite, rutile, zircon, and monazite present as accessory phases. Phyllosilicate bands define schlieren, along with smaller grain-size quartz, K-feldspar, and albite than in the leucosome. Biotite is variably chloritized, with grains within the leucosomes showing greater alteration. Quartz grains show undulose extinction throughout the sample.

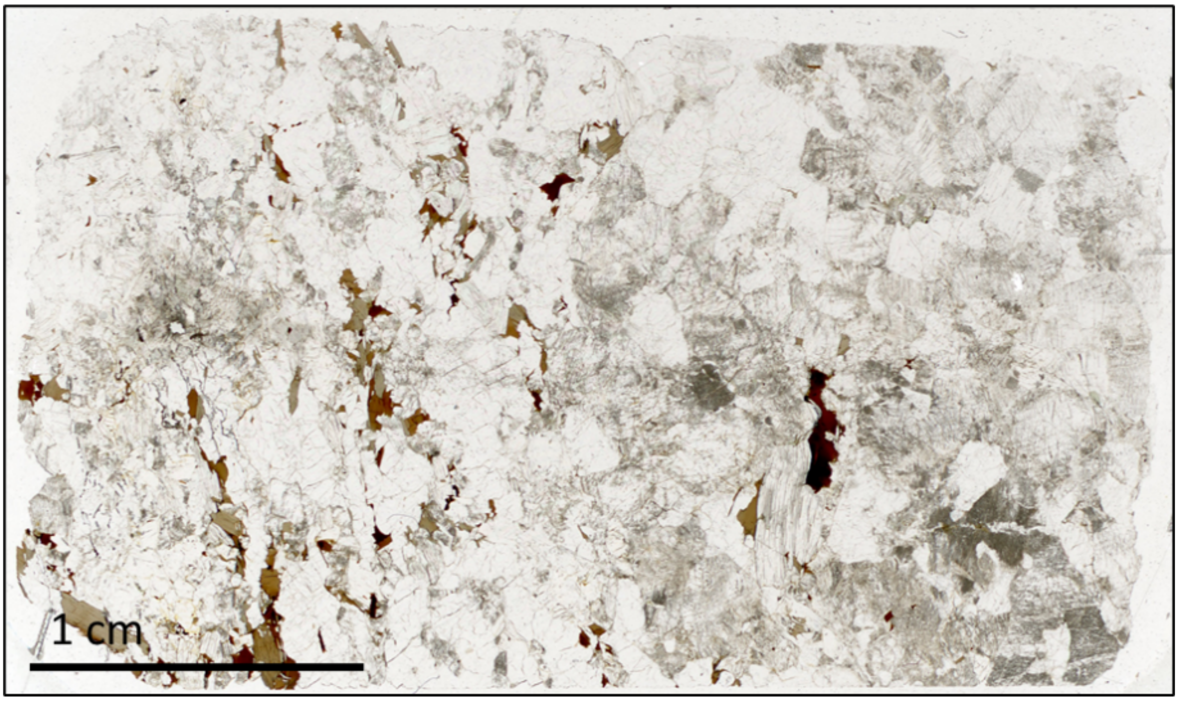

Figure S1.11 – Thin section image of migmatite sample 04a, displaying biotite schlieren and divide between fine-medium-grained mesosome (left) and coarse-grained leucosome (right).

K-feldspar in the leucosome is perthitic with albite lamellae and partially sericitized. Grains are commonly porphyroblastic, between 1.9-3.7 mm, with fine inclusions of plagioclase, quartz, and muscovite (Figure S1.12e). In schlieren/mesosome domains, K-feldspar form loose clusters, with grains 0.5-1.0 mm across. Complex sericite boundaries and quartz films occur along contacts with muscovite (Figure S1.12a). Plagioclase forms 1.6-4.4 mm highly sericitized grains in the leucosome. It is rare in the mesosome, with grains of 0.3-0.6 mm with K-feldspar overgrowths (Figure S1.12b).

Muscovite forms 1.3-4.3 euhedral laths in the leucosome, associated with K-feldspar and biotite. In the mesosome, grains are smaller, 0.5-1.0 mm, subhedral, and are sericitized along boundaries with K-feldspar. Biotite grains are mostly in the mesosome/schlieren, some up to 1.8 mm across. Grain boundaries are commonly irregular, especially for chloritized grains in the leucosome. Both biotite and chlorite can be sagenitic, with acicular rutile also occurring in quartz (Figure 2.21c-d). Sillimanite forms fibrous clots up to 2 mm long, with fine muscovite/sericite and K-feldspar inclusions. Tourmaline is rare and strongly pleochroic, with blue-green cores and brown-green rims. Grains in the leucosome are up to 1.7 mm across.

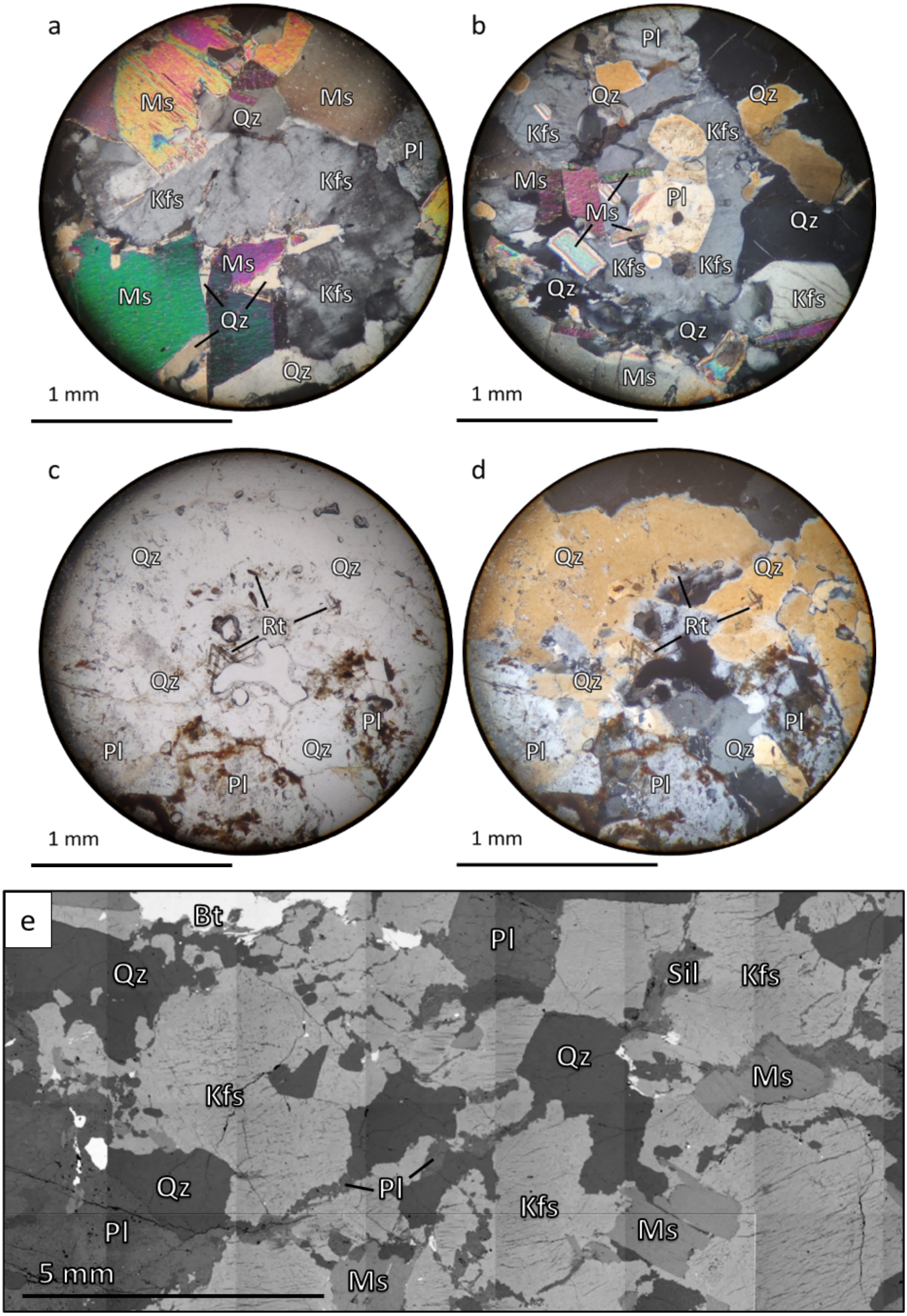

Figure S1.12 – Photomicrographs and BSE image of various mineral textures in sample 04a. a) Quartz films and sericite along muscovite boundaries with K-feldspar (XPL). b) Plagioclase with K-feldspar overgrowth/replacement in the mesosome (XPL). c) Acicular rutile inclusions in quartz (PPL). d) Acicular rutile inclusions in quartz (XPL). e) BSE image of perthitic K-feldspar porphyroblasts in the leucosome.

Sample 04b is a medium-grained *in-situ* schollen diatexite composed of K-feldspar + quartz + plagioclase + biotite + muscovite + sillimanite + tourmaline, with apatite, rutile, zircon, and monazite present as accessory phases. Schollen mesosomes and leucosomes form discrete domains with transition zones and schlieren (Figure A1.13). Biotite is variably chloritized, with grains within the leucosomes showing greater alteration. Quartz grains show undulose extinction throughout the sample.
In the leucosome, K-feldspar forms partially sericitized medium-coarse grains up to 5.7 mm across. These porphyroblastic grains have perthitic albite lamellae and complex

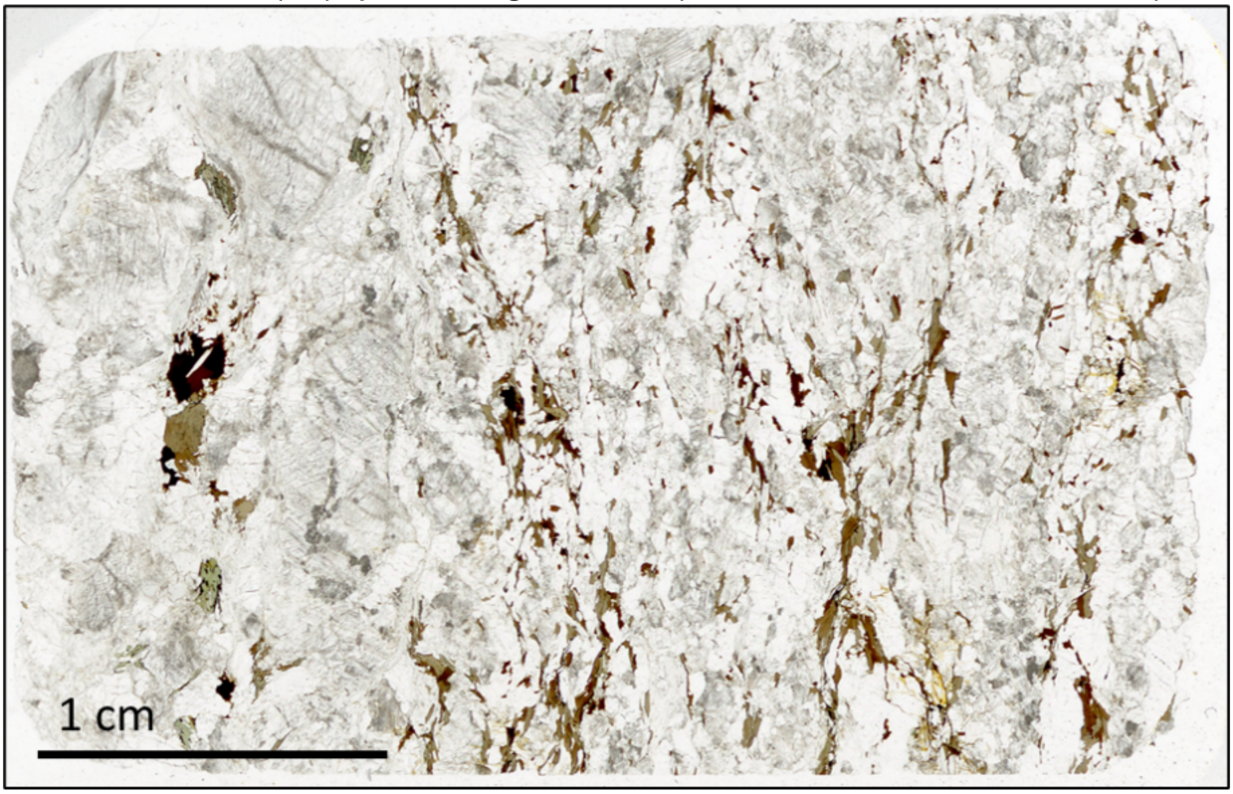

Figure A1.13 – Thin section image of migmatite sample 04b, displaying leucosome (left) and mesosome schollen (right). Biotite marks the primary fabric of the mesosome and leucosome schliere (where disaggregated).

contacts with sericite/muscovite and sillimanite (Figure A1.14a-b). Grains commonly have inclusions of plagioclase, quartz, and sericitized muscovite. Plagioclase grains in the leucosome are partially sericitized, 1.2-1.9 mm across, and form sporadic myrmekites along boundaries with K-feldspar. In schollen mesosome domains, K-feldspar grains, typically 0.7-1.3 mm, form loose clusters with quartz, muscovite, and minor biotite, up to 12.3 mm long and 3.5 mm wide (Figure A1.14c-e). These grains show crosshatch twinning, perthitic albite, or neither of these features. Plagioclase is absent from the K-feldspar-quartz-muscovite clusters but occurs outside of them as fine-medium grains, 0.8-1.2 mm across, with partial sericitization. K-feldspar and plagioclase grains in the transitional mesosome are a mixture of the textures and grain sizes present in the leucosome and schollen domain, with the notable difference that the K-feldspar-quartz-muscovite clusters appear to be disaggregated.

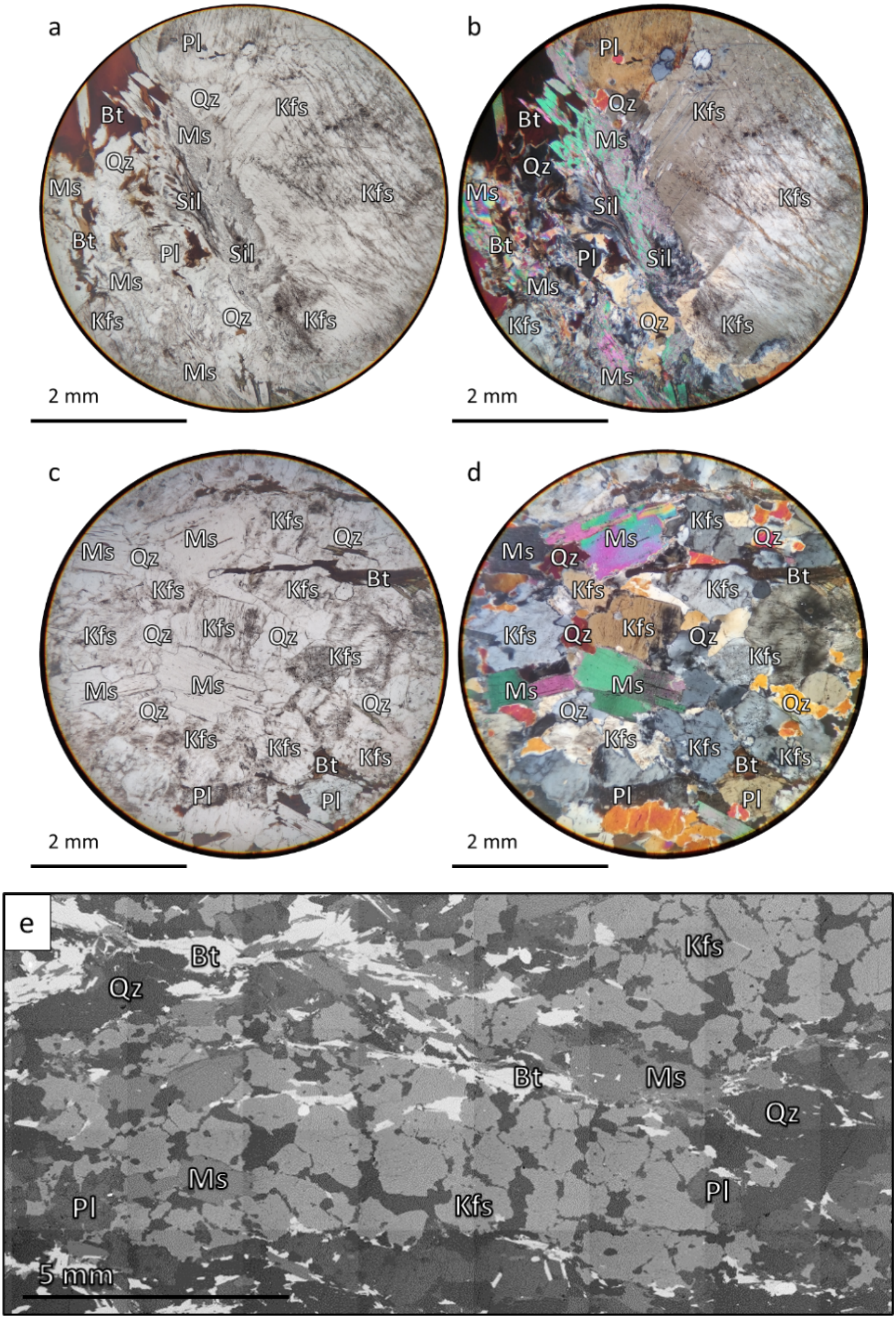

Figure S1.14 – Photomicrographs and BSE image of various mineral textures in sample 04b. a) Perthitic K-feldspar porphyroblast and sillimanite-sericite clot (PPL). b) Perthitic K-feldspar porphyroblast and sillimanite-sericite clot (XPL). c) Cluster of K-feldspar grains with muscovite and quartz (PPL). d) Cluster of K-feldspar grains with muscovite and quartz (XPL). e) BSE image of K-feldspar clusters (light grey) in the mesosome.

Biotite forms fine-medium grains, 0.5-1.6 mm, with irregular boundaries in schollen and schlieren. Sagenitic textures are common in both biotite and chlorite. Muscovite forms medium laths typically 1.0-1.4 mm long in the schollen and transitional mesosome, with thin sericite rims and quartz films along contacts with K-feldspar. In the leucosome, muscovite is medium-coarse, with grains up to 5.6 mm long. Intergrowths of sericite and sillimanite form fibrolitic clots that border perthitic K-feldspar. Tourmaline is rare and pleochroic, with small blue-green cores and brown-green rims. Grains in the leucosome are 0.5-1.4 mm.

Locality 05

Sampling locality 05 is 0.5 km west of Mana (30.77444°N, 79.48951°E). The exposure is a ~10 m wide alcove in the cliff face with surface mineralisation, beneath which is a strongly layered migmatite with alternating leucosome and mesosome bands 1-3 cm wide (Figure S1.15).

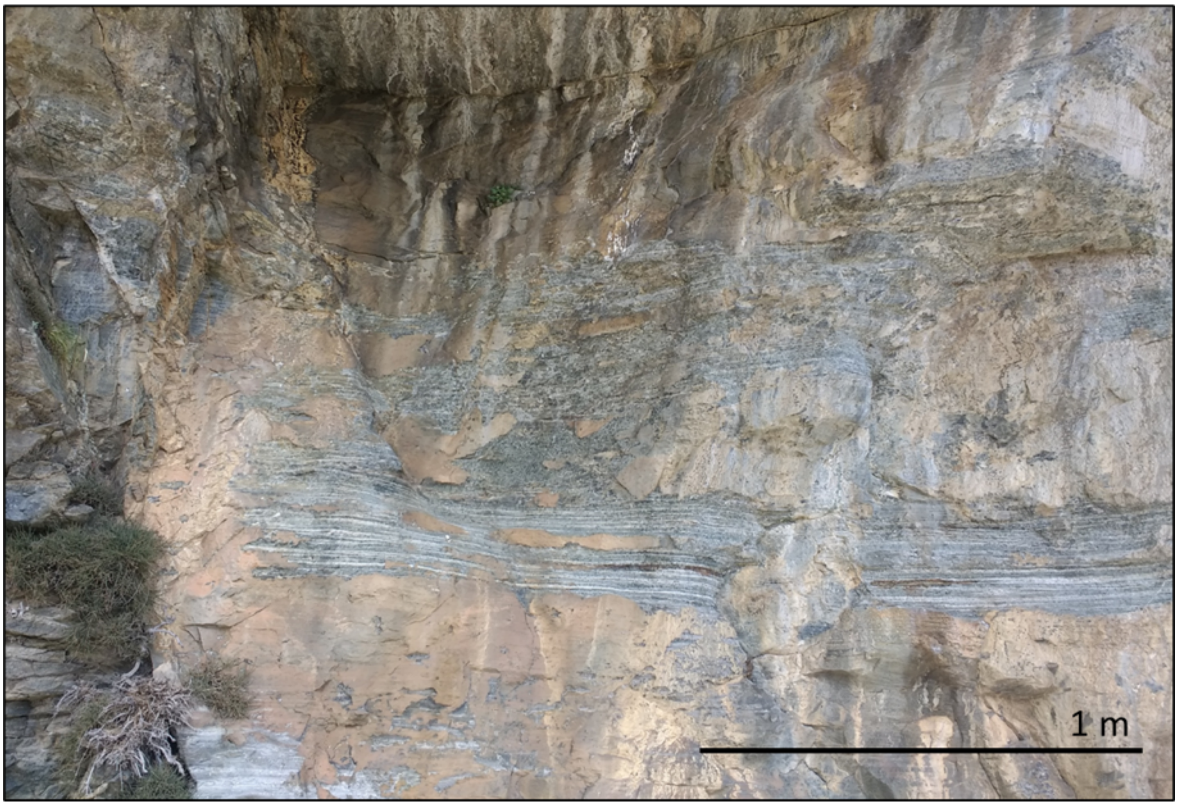

Figure S1.15 – Locality 05 (30.77444°N, 79.48951°E). Sampled strongly layered migmatite with alternative leucosomes and melanosomes. Surface mineralisation obscures most of the underlying migmatite.

Sample 05b is an *in-situ* medium-grained melanocratic migmatite sampled from the layered migmatites at locality 05. This sample comprises quartz + plagioclase + biotite + muscovite + K-feldspar + tourmaline + sillimanite, with apatite, ilmenite, zircon, and monazite present as accessory phases. K-feldspar, muscovite, tourmaline, and sillimanite are concentrated in the 1 cm wide leucosome domain, with less biotite. Biotite grains within the leucosome are strongly chloritized. Quartz grains show undulose extinction throughout the sample.

K-feldspar grains are 0.5-1.2 mm across and only present in leucosomes, with extensive muscovite alteration (Figure S1.16a-b). The remaining grains show either continuous extinction or crosshatch twinning, as well as irregular boundaries with plagioclase and partial sericitization. In the leucosome, plagioclase forms grains 0.5-1.2 mm across which are strongly sericitized with complex boundaries with K-feldspar and is also present as inclusions in muscovite-altered K-feldspar. Outside of the leucosome, plagioclase is typically coarser, 0.6-1.5 mm, with albite twinning and sericite alteration.
Biotite forms fine laths, 0.2-0.8 mm long, with the small grains concentrated in and around the leucosome. Grains in the meso-melanosome are regular and form thin phyllosilicate domains. Muscovite in the leucosome forms either grains 0.7-1.1 mm

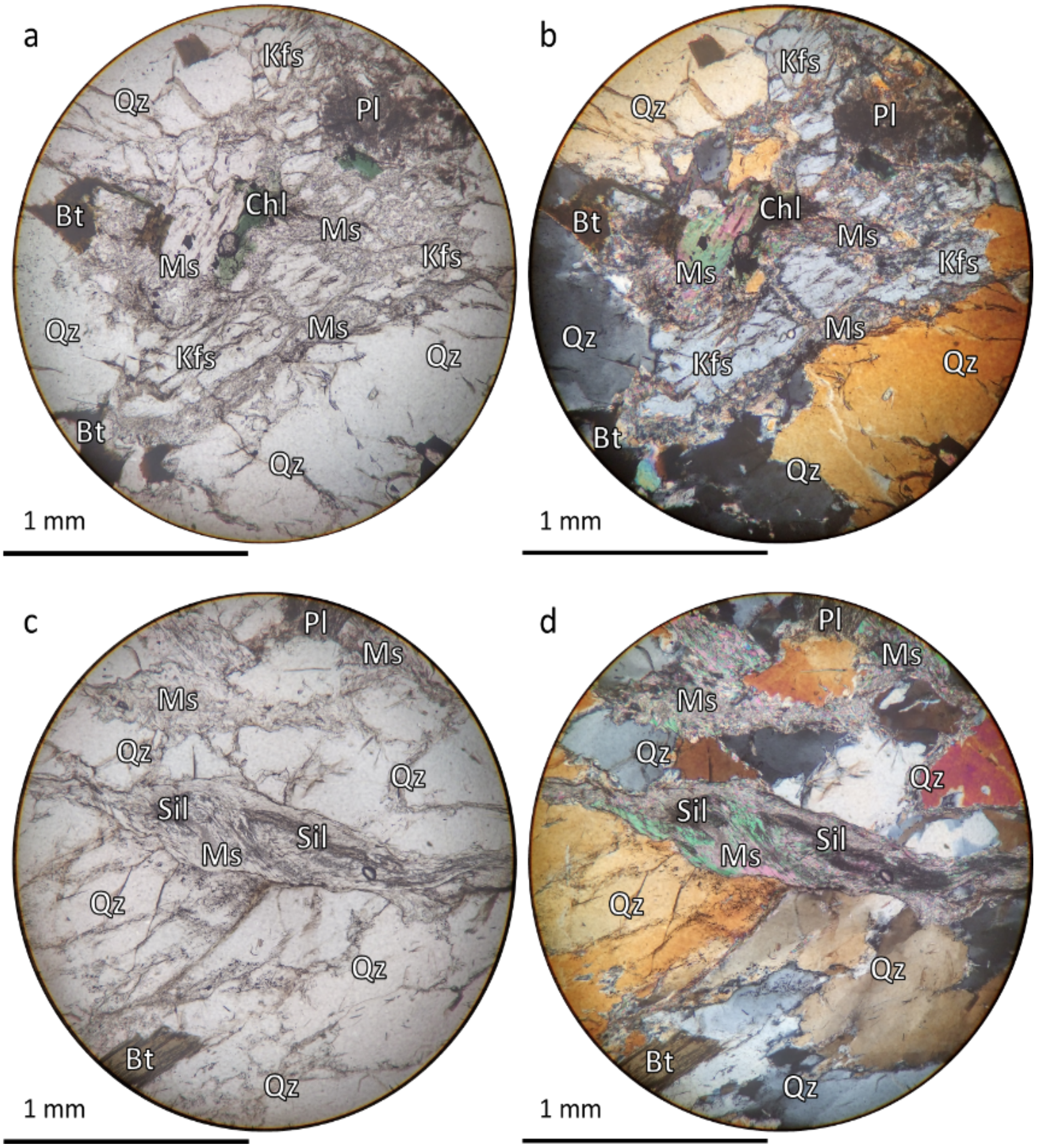

Figure S1.16 – Photomicrographs of various mineral textures in sample 05b. a) K-feldspar grain with extensive sericite alteration and replacement by muscovite (PPL). b) K-feldspar grain with extensive sericite alteration and replacement by muscovite (XPL). c) Sillimanite clot with sericite alteration (PPL). d) Sillimanite clot with sericite alteration (XPL).

across, or sericite clots with sillimanite, where it is up to 6 mm long and 300 μm wide

(Figure S1.16c-d). Small muscovite grains are also present in biotite-rich areas in the meso-melanosome. Tourmaline is only present in the leucosome, with widely variable prismatic grain sizes, 0.5-2.8 mm in cross-section, strong pleochroism and blue-green colouration.

Locality 06

Sampling locality 06 is 0.8 km west of Mana (30.77517°N, 79.48624°E). Samples were taken from 1-2 m diameter scree boulders directly downhill from a leucocratic scarp in the cliff to the north. The boulders are garnet-tourmaline leucogranite with sub-parallel tourmaline-rich horizons up to 0.5 cm wide and garnets with core-rim zoning, 1-8 mm in diameter (Figure S1.17).

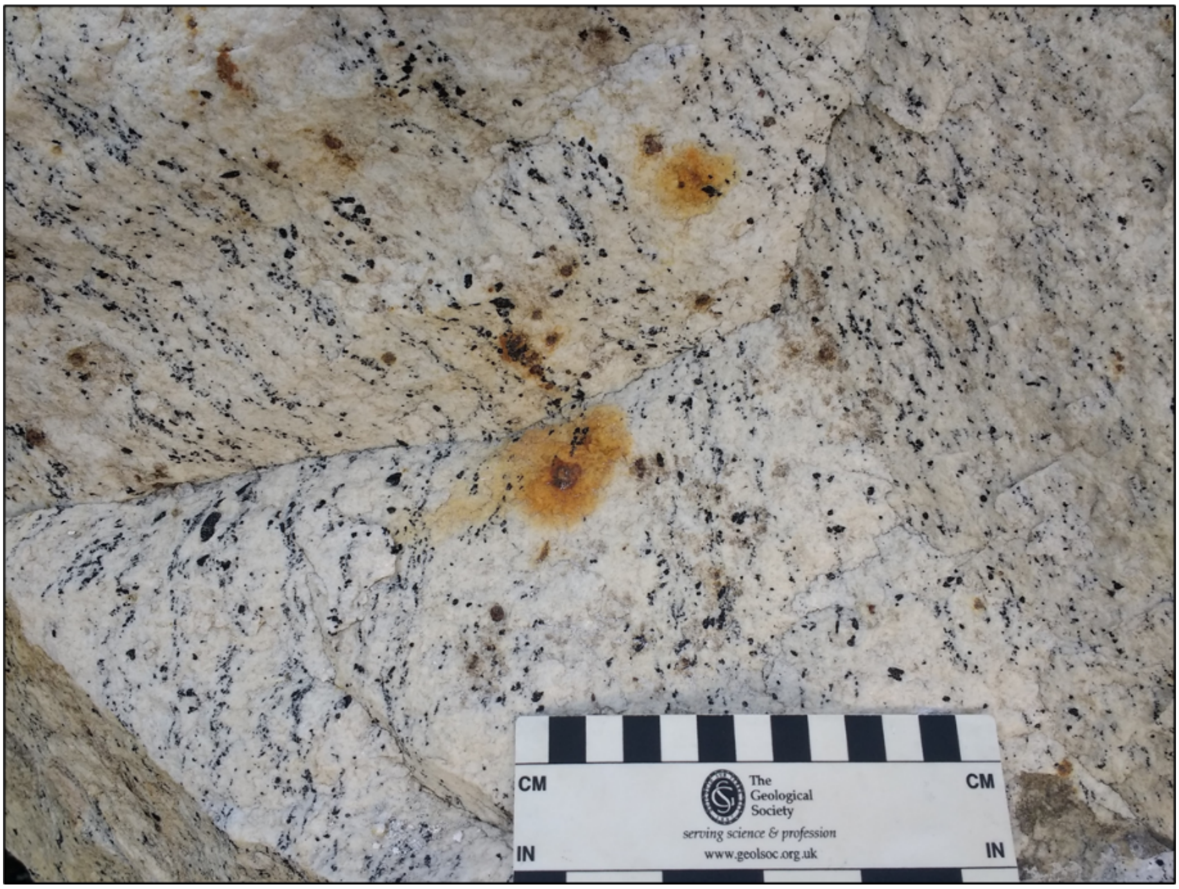

Figure S1.17 – Locality 06 (30.77517°N, 79.48624°E). Sampled boulder of garnet-tourmaline leucogranite, with sub-parallel tourmaline horizons and surface alteration halos around larger garnet grains.

Sample 06 is a medium-grained leucogranite taken from a boulder at locality 06, with garnet abundances and tourmaline-rich layers that are typical for the locality. The granite consists of plagioclase + quartz + K-feldspar + tourmaline + garnet, with minor muscovite (Figure S1.18). Zircon and monazite are present as accessory phases.
K-feldspar forms grains 1.2-2.4 mm across, rarely up to 6 mm, with perthite and crosshatch twinning both present. The larger grains are euhedral with quartz, plagioclase, and muscovite/sericite inclusions and full perthitic albite exsolution lamellae (Figure S1.19a-b). Plagioclase forms grains 0.9-2.3 mm across with albite twinning common and

as well as inclusion-poor grains up to 4.7 mm across with albite and simple twins. Myrmekitic plagioclase is common along boundaries with K-feldspar.
Muscovite is present as a few small grains, 0.2-0.4 mm long, or as sericitized microporous vein perthite associated with particularly large K-feldspar grains.

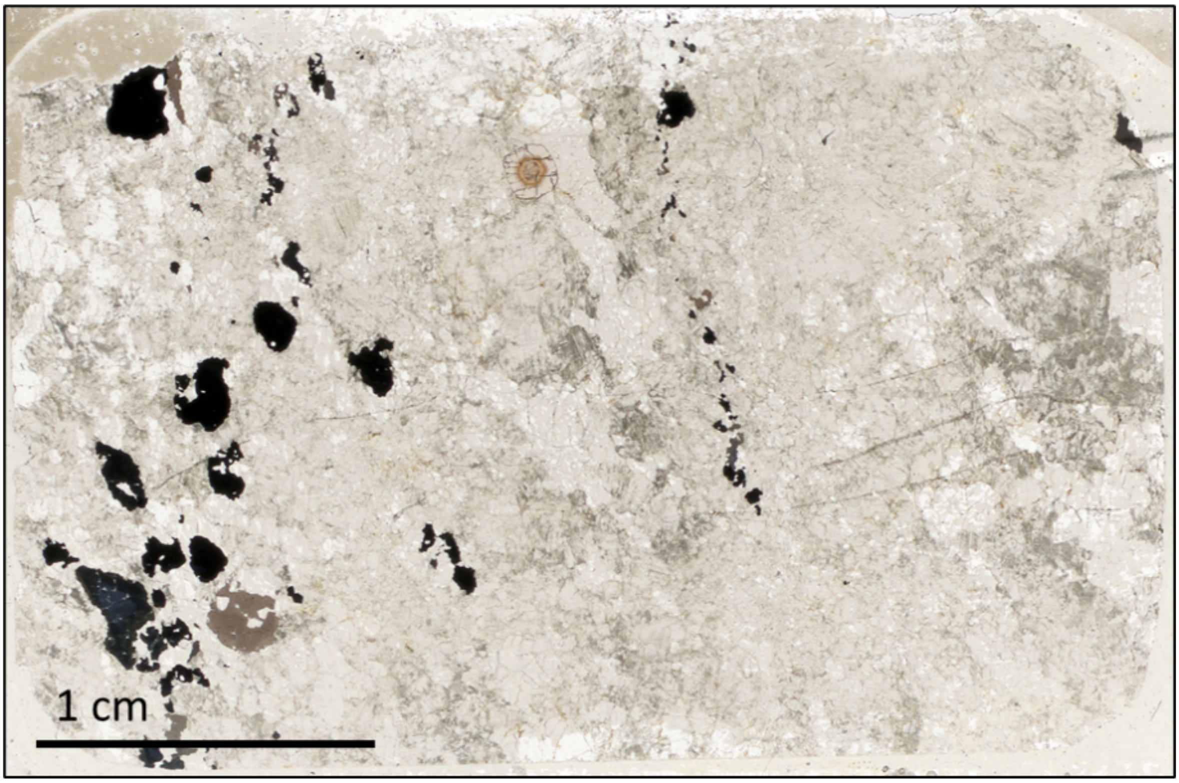

Figure S1.18 – Thin section image of leucogranite sample 06, displaying zoned pleochroic tourmaline (left) and euhedral garnet grain (centre-top).

Tourmaline forms prismatic grains typically 0.4-1 mm in diameter in section and up to 3 mm in diameter and 10 mm long in hand specimen. These grains are strongly pleochroic with blue cores, dark blue rims, and a grey border (Figure S1.19c-d).

Garnets form sub- to euhedral grains, 2-8 mm in diameter, but typically 3-4 mm. There is distinct core-rim zoning, with orange-coloured cores and pale pink rims. Along core-rim boundaries is a zone of crystallographically oriented rutile needles, starting just inside the core region and extending to <100 μm into the rim (Figure S1.19e-f). The rims are typically inclusion poor, but some grains show irregular lobate quartz. BSE imaging of these garnets, isolated in resin mounts, reveals the cores to be rich in fine, randomly-oriented inculsions typically <10 μm in diameter (Figure S1.19g).

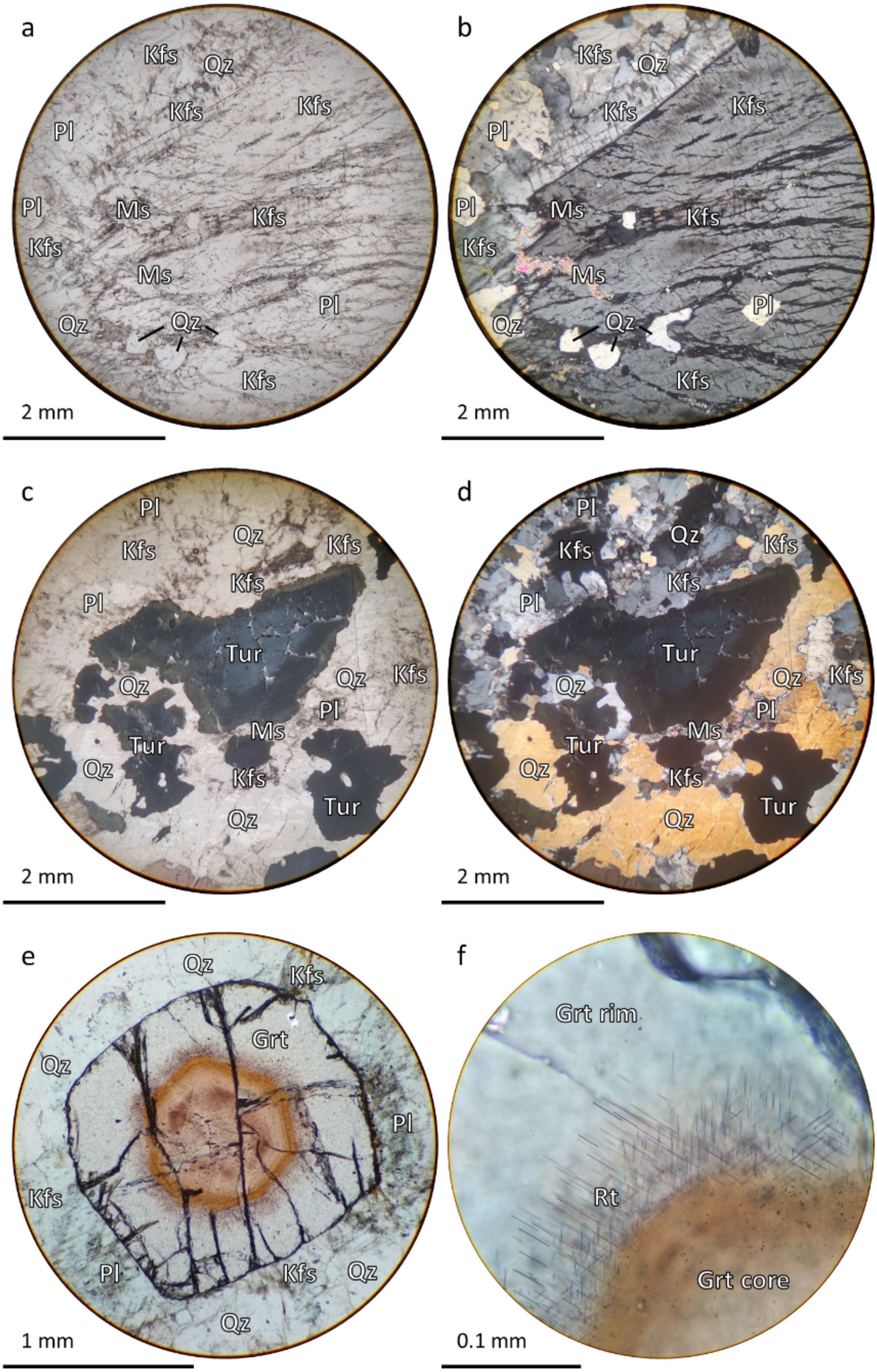

Figure S1.19– Photomicrographs and BSE image of various mineral textures in sample 06. a) Coarse-grained euhedral K-feldspar with perthitic albite (PPL). b) Coarse-grained euhedral K-feldspar with perthitic albite (XPL). c) Coarse-grained tourmaline with core-rim zoning (PPL). d) Coarse-grained tourmaline with core-rim zoning (XPL). e) Euhedral garnet with distinct core (orange) and rim (PPL). f) Crystallographically oriented rutile needles at garnet core-rim boundary (PPL).

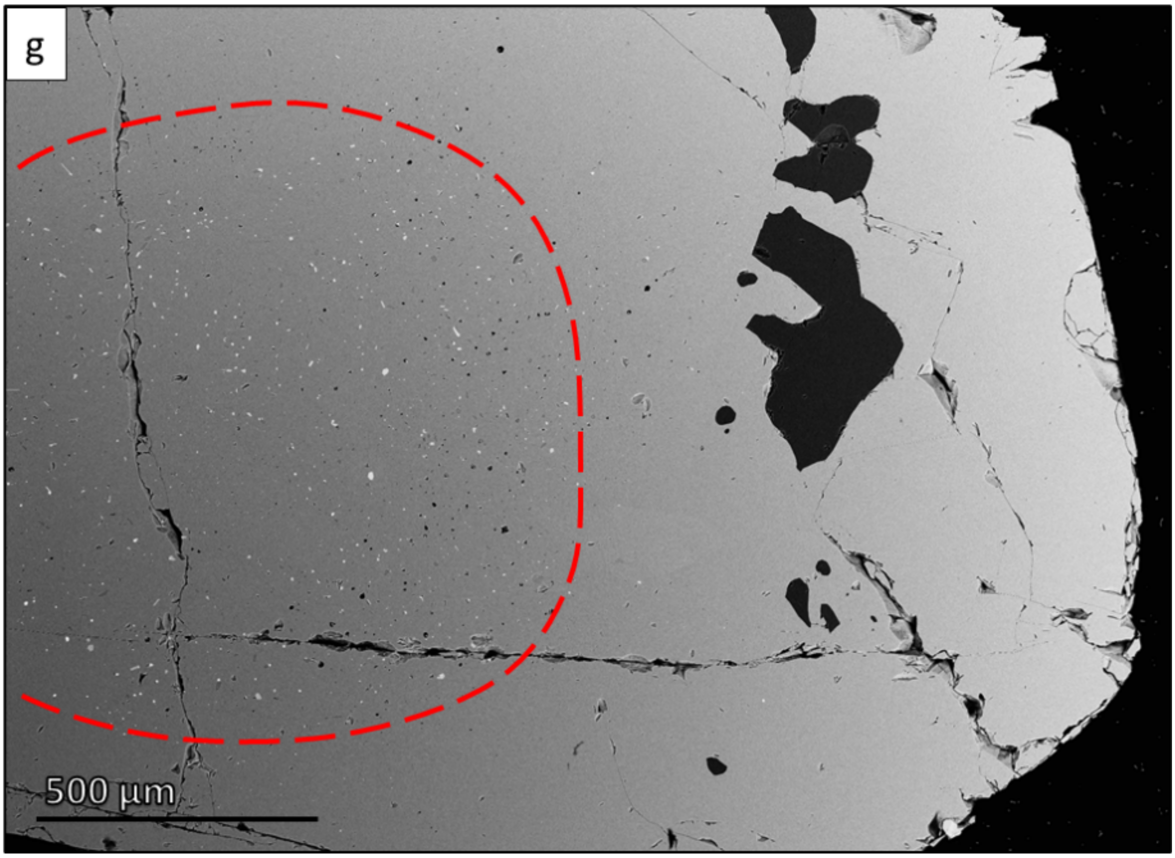

Figure S1.19 cont. – g) BSE image of a separated garnet grain in resin, from sample 06, with a core rich in randomly oriented micro-inclusions and irregular oblate quartz in the rim.

Locality 07

Sampling locality 07 is 1.8 km west-northwest of Mana (30.78055°N, 79.47762°E). The locality is centred on a 2.5-3.0 m wide leucocratic dyke exposed at the base of the cliff on the north side of the Alaknanda valley. This exposure features diatexites crosscut by a leucogranite dyke and associated disseminated dykelets that vary in size down to the centimetre scale, where they become indistinguishable from migmatite leucosomes (Figure S1.20). Similar crosscutting leucocratic features are present higher up the face of the cliff.

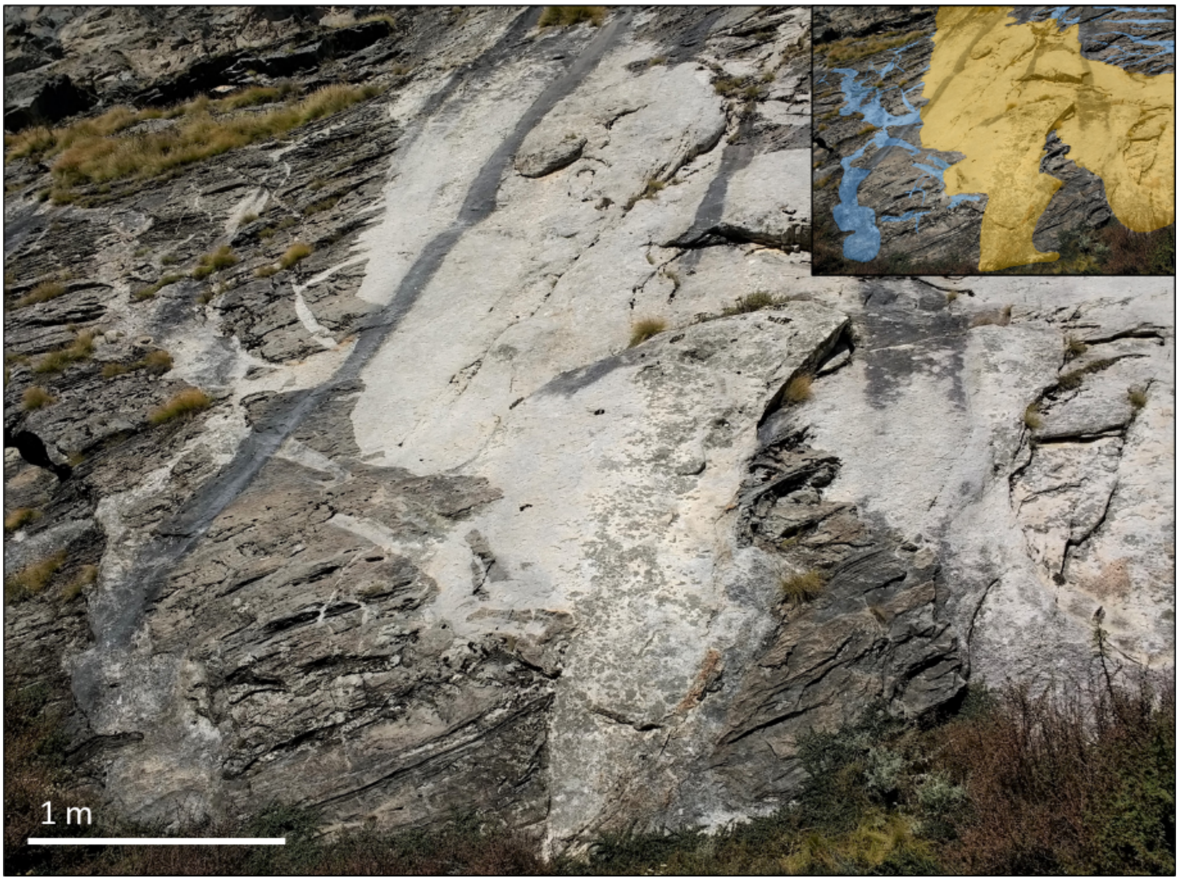

Figure S1.20 – Locality 07 (30.78055°N, 79.47762°E). Sampled outcrop with central leucocratic dyke (inset – yellow) and disseminated dykelets (inset – blue), cross-cutting the host migmatite.

Sample 07a is a medium-grained *in-situ* schlieren diatexite sampled from the crosscut migmatite, proximal to centimetre-scale dykelets, consisting of quartz + plagioclase + K-feldspar + biotite + muscovite + sillimanite. Apatite, zircon, and rutile are present as accessory phases. Phyllosilicate bands with sillimanite define schlieren that breaks up the feldspar-dominated leucosome (Figure S1.21). Biotite is variably chloritized, with grains within the leucosomes showing greater alteration. Quartz grains show undulose extinction throughout the sample.

K-feldspar grains are typically 1.1-1.4 mm across with crosshatch twinning or perthite albite lamellae. Coarser grains, up to 2.5 mm in length, having microporous vein perthite. These grains form clusters up to 10 mm wide and longer than the width of a thin section with quartz, plagioclase, and muscovite (Figure S1.22a-b & e). Grains are only weakly

sericitized, concentrated along perthitic and twinning structures. Plagioclase forms 0.6-1.2 mm grains that are strongly sericitized with albite twinning common. Irregular blebs of plagioclase are present as inclusions in K-feldspar in the clusters, with myrmekitic grains around the edges of the clusters. Plagioclase is more abundant away from these features.

Biotite forms 0.4-1.0 mm laths in phyllosilicate-rich schlieren and biotite-sillimanite selvedges along the edges of the K-feldspar clusters (Figure S1.22c-d). Chlorite is commonly sagenitic, while biotite is only rarely so. Muscovite as either 0.3-0.6 mm laths interlocking with biotite in schlieren, or as sericite clots with sillimanite, up to 9 mm long and 1.5 mm thick, or as pockmarked grains associated with K-feldspar alteration, up to 0.8 x 1.2 mm. Sillimanite is present as fibrolite in the previously mentioned clots, surrounded by sericite, within biotite-rich selvedges.

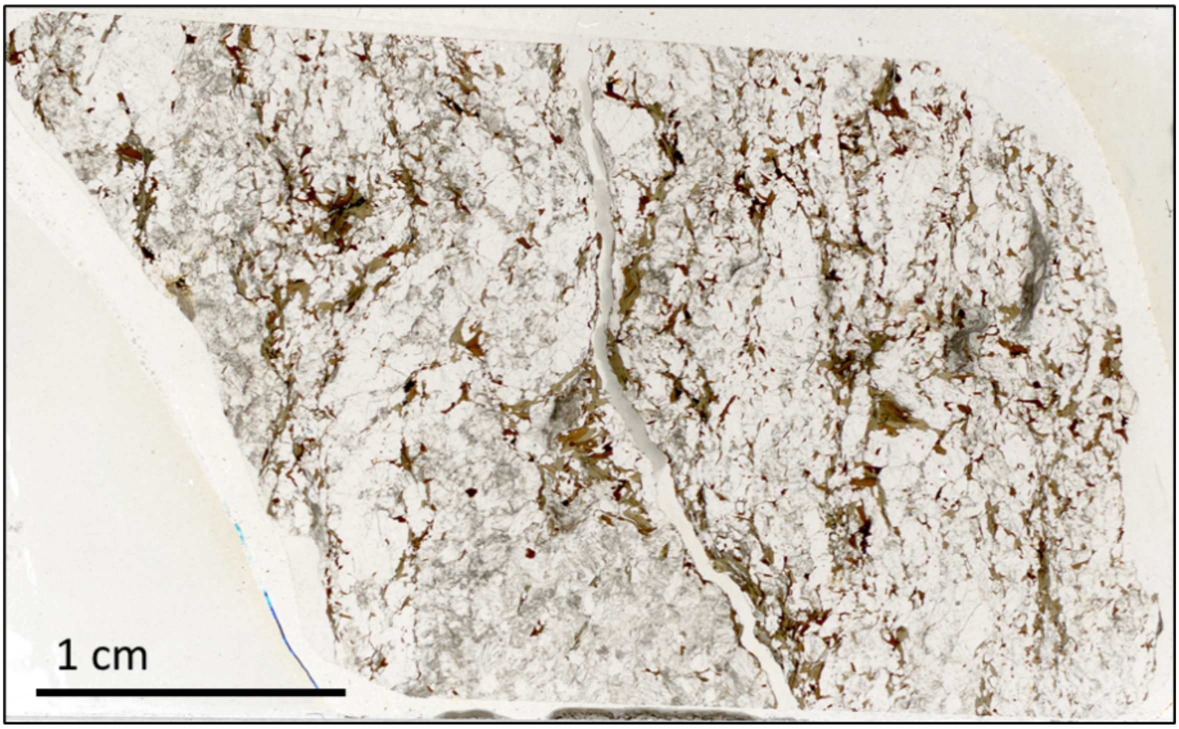

Figure S1.21– Thin section image of migmatite sample 07a, displaying undulating sillimanite and phyllosilicate-rich layers between leucosomes.

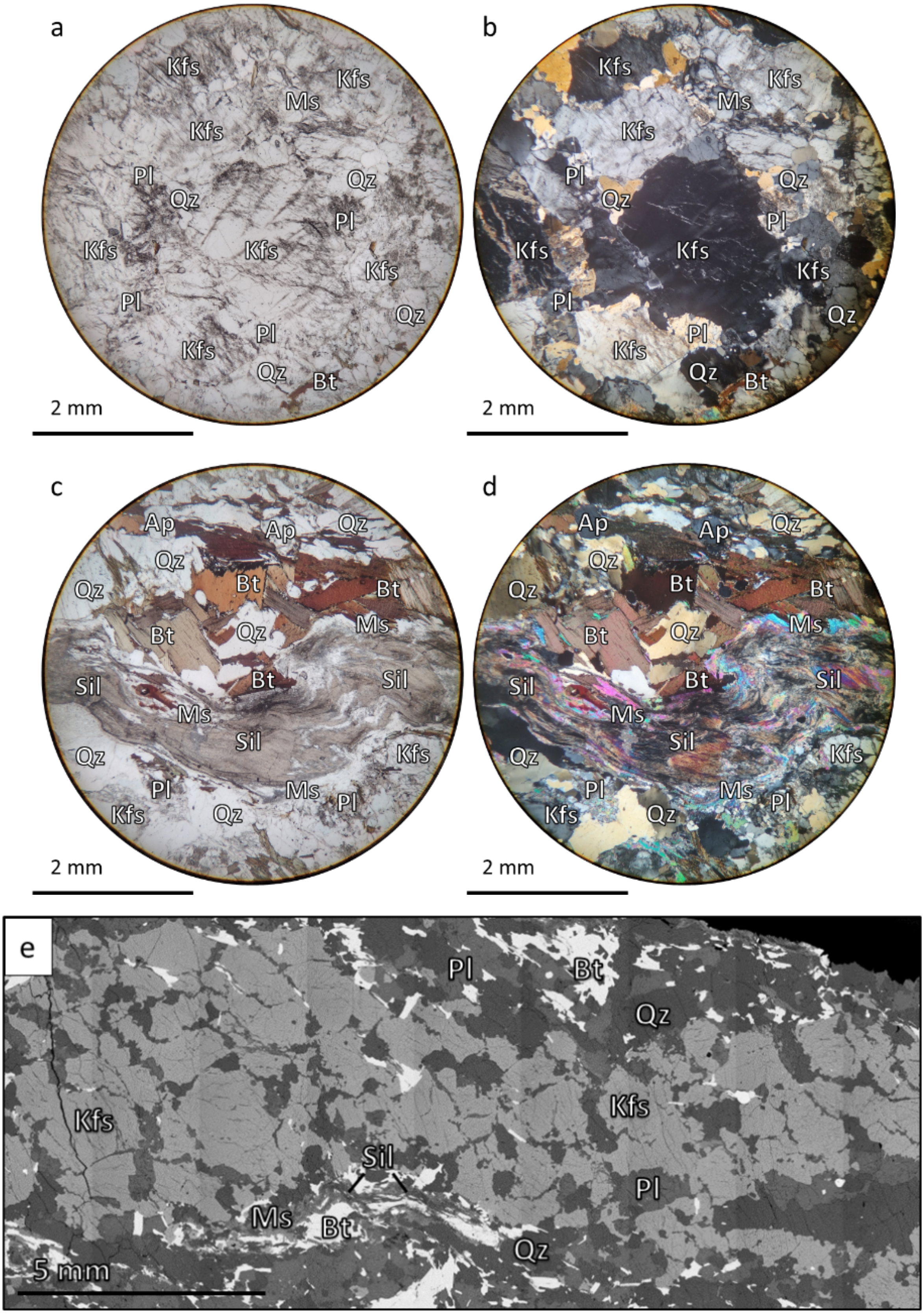

Figure S1.22 – Photomicrographs and BSE image of feldspar and sillimanite textures in sample 07a. a) Clusters of perthitic K-feldspar grains and irregular Pl + Qz + Ms (PPL). b) Clusters of perthitic K-feldspar grains and irregular Pl + Qz + Ms (XPL). c) Fibrolitic sillimanite and biotite selvedge along the edge of a K-feldspar cluster (PPL). d) Fibrolitic sillimanite and biotite selvedge along the edge of a K-feldspar cluster (XPL). e) BSE image of a lenticular K-feldspar cluster, >2 cm across, with sillimanite-biotite selvedge along its lower boundary.

Sample 07b is an *in-situ* medium-grained leucogranite taken from the leucogranite dyke. It contains K-feldspar + quartz + plagioclase + biotite + muscovite + tourmaline, with minor sillimanite. Apatite, zircon, monazite, and rutile are present as accessory phases. Biotite is partially chloritized, with ~20% of grains affected. Quartz grains show undulose extinction throughout the sample.

K-feldspar forms subhedral rectangular laths, 0.9-3.1 mm, with perthitic albite exsolution lamellae and crosshatch twinning common. Some grains form microporous vein perthite. Larger grains have inclusions of plagioclase, quartz, and muscovite (Figure S1.23-b). Partial sericitization is prevalent along albite lamellae and contacts with muscovite and chlorite. Plagioclase grains are 0.9-2.5 mm across, strongly sericitized, and form complex boundaries with K-feldspar with myrmekitic plagioclase grains up to 530 µm across.

Biotite grains are irregular laths, typically 0.7 mm long, dispersed throughout the rock, often interlocking with muscovite. Chloritized grains are commonly sagenitic. Muscovite is present as (i) grains interlocking with biotite, typically 0.6 mm long, (ii) as sericite clots with sillimanite, up to 6.7 mm long and 0.3 mm wide, or (iii) as pockmarked grains, 0.9-1.8 mm across, associated with K-feldspar alteration (Figure S1.23c-d). Tourmaline forms pleochroic grains 0.4-1.5 mm across, that are brown-green in colour with rare blue-green cores. Sillimanite is present as both elongate fibrolite schlieren (Figure S1.23e) and prismatic crystals in the cores of sericite clots. The largest sillimanite grain is 400 x 35 µm.

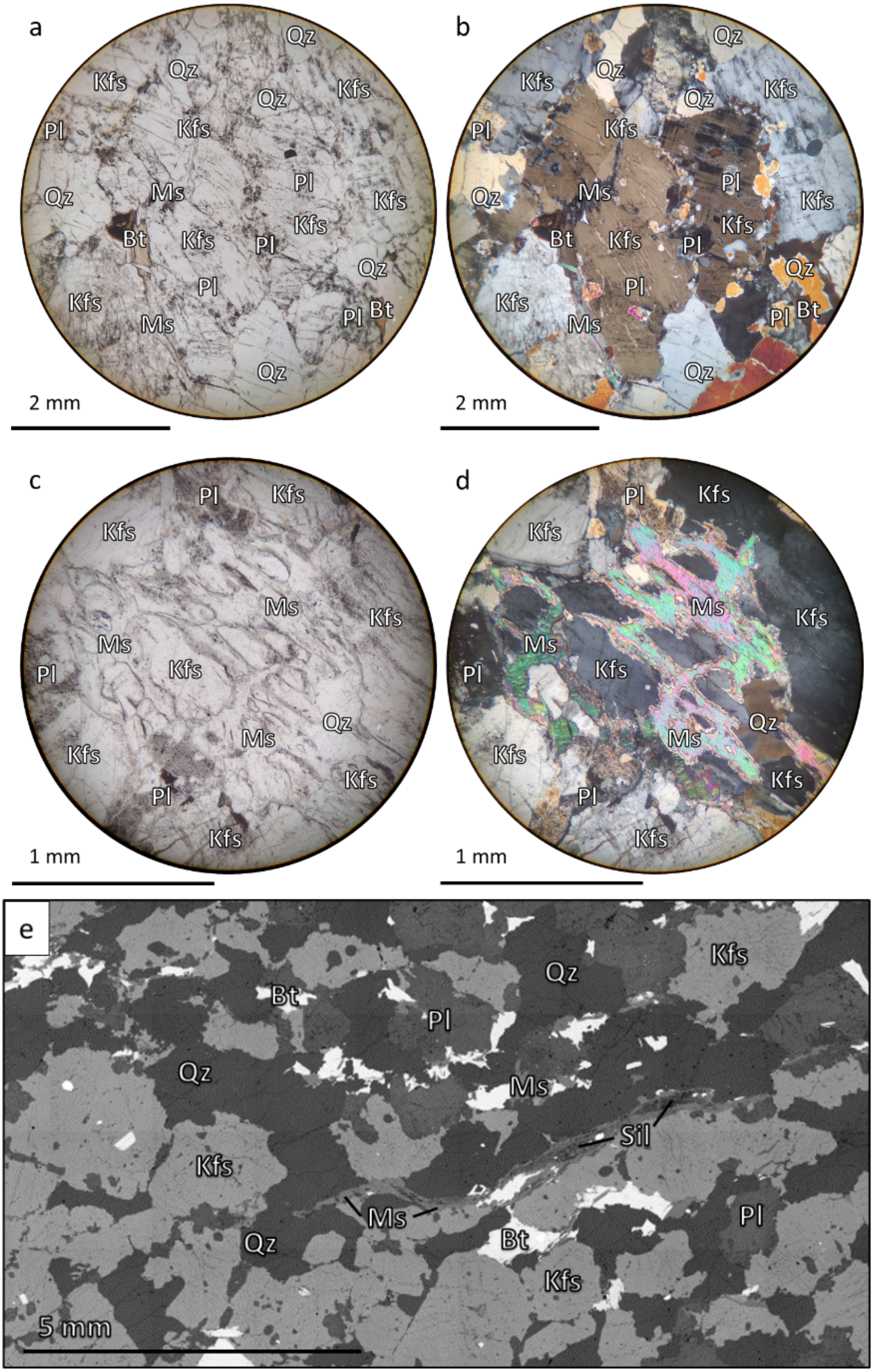

Figure S1.23 – Photomicrographs and BSE image of various mineral textures in sample 07b. a) Coarse-grain perthitic K-feldspar with inclusions of Pl + Qz + Ms (PPL). b) Coarse-grain perthitic K-feldspar with inclusions of Pl + Qz + Ms (XPL). c) Replacement of K-feldspar with muscovite (PPL). d) Replacement of K-feldspar with muscovite (XPL). e) BSE image of sillimanite schliere with sericite alteration.

Sample 07c is a medium-grained *in-situ* diatexite sampled from the crosscut migmatite, distal to centimetre-scale dykelets, consisting of plagioclase + quartz + biotite + sillimanite + K-feldspar + muscovite, with minor garnet. Apatite, zircon, monazite, and rutile are present as accessory phases. Biotite and sillimanite form melanocratic selvedges that line irregular medium- to coarse-grained leucosomes (Figure S1.24). There is only minor chloritization of biotite, predominantly of biotite adjacent to leucosomes. Quartz grains show undulose extinction throughout the sample.
Only a few instances of K-feldspar are present in thin sections as subhedral 0.9-2.8 mm

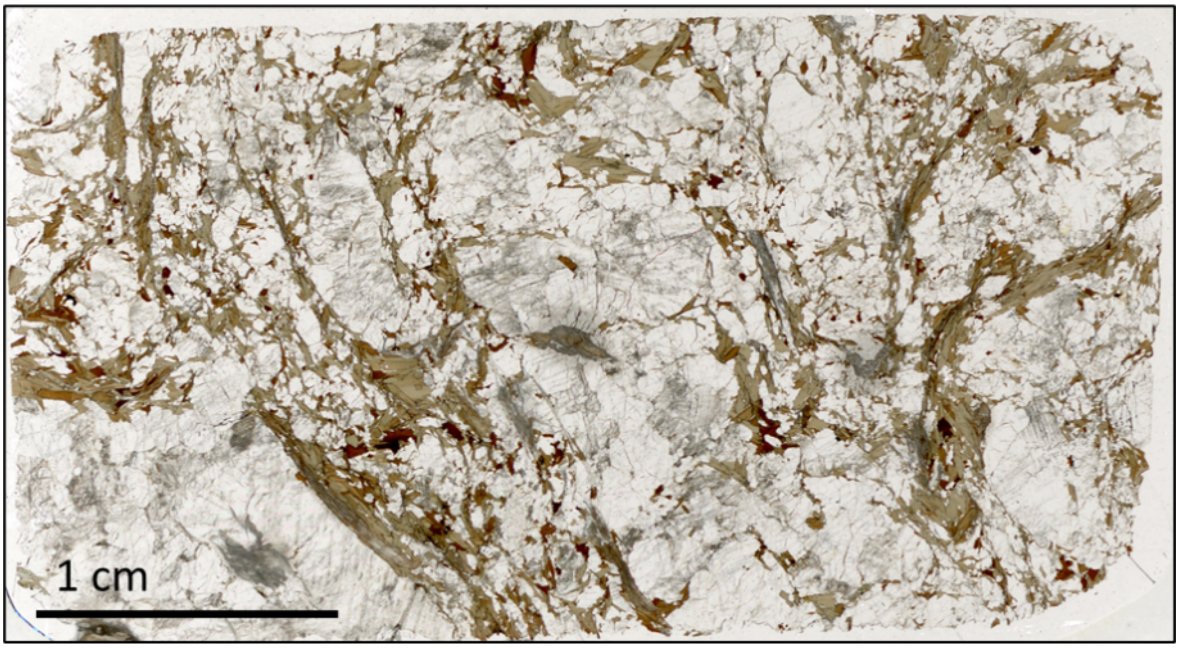

Figure S1.24 – Thin section images of migmatite sample 07c, displaying irregular leucosomes lined with sillimanite and biotite selvedges.

grains in the leucosomes. All grains are inclusion poor, with perthitic albite exsolution lamellae and partial sericitization along these features (Figure S1.25a-b). Plagioclase typically forms 1.1-3.5 mm subhedral grains, although there are also euhedral grains up to 9.2 mm across. Grains have inclusions of quartz, biotite, and sillimanite. Albite twins are common, and smaller grains are strongly sericitized. Myrmekitic plagioclase is present along grain boundaries with K-feldspar.

Biotite is largely absent from the leucosomes, apart from rare small xenocrystic grains, and concentrated in biotite-sillimanite selvedges and adjacent melanosomes. Biotite laths are typically 0.9-1.3 mm long, with sagenitic textures present in partially and fully chloritized grains. Muscovite is present only as <200 μm laths interlocked with biotite and as strong sericite alteration of feldspar. Garnet grains are rare, <300 μm in diameter, fractured and partially resorbed, with biotite and chlorite (Figure 2.40c-d). Sillimanite forms fibrolite clots with biotite and sericite up to 8 mm long (Figure S1.25e).

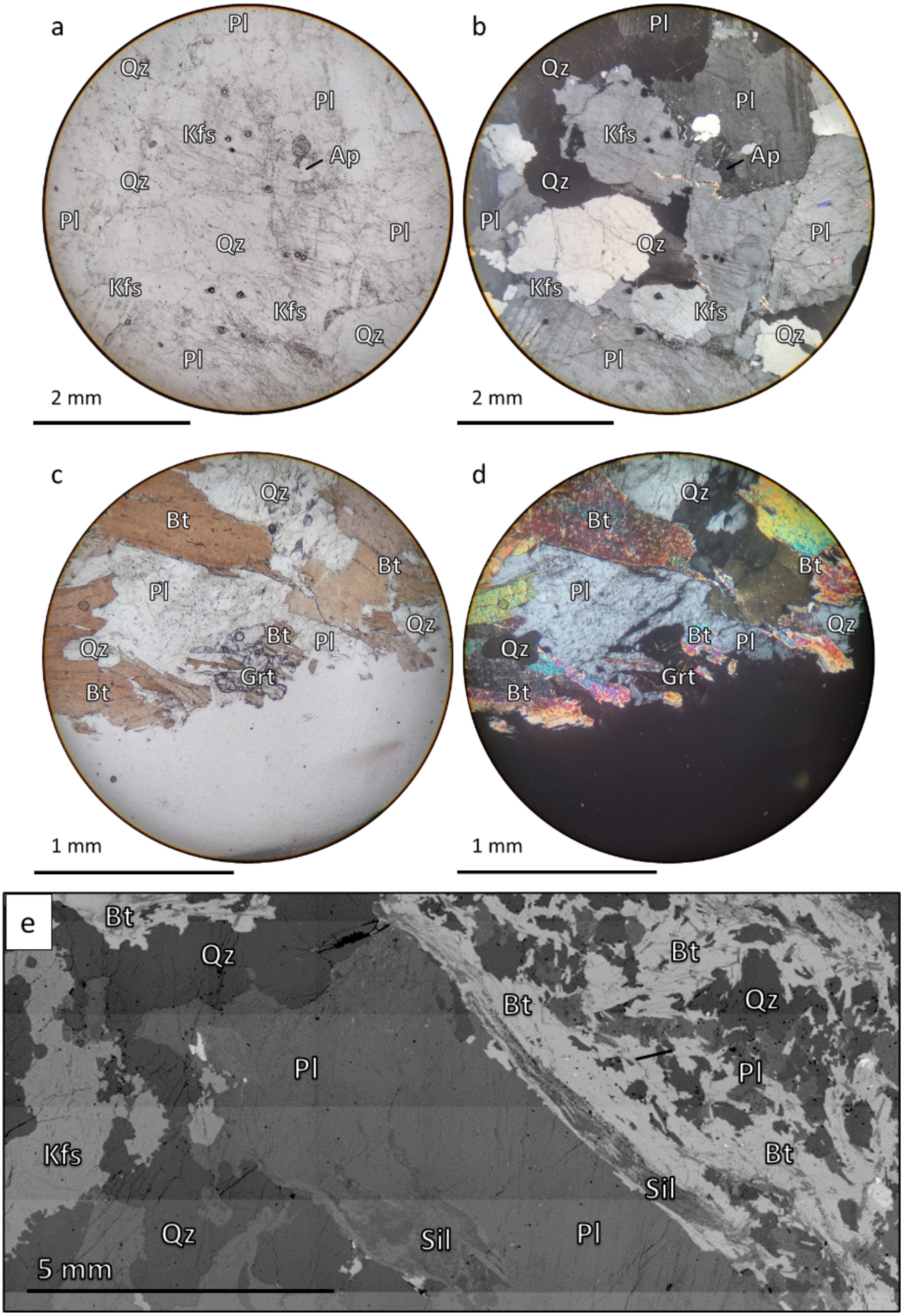

Figure S1.25 – Photomicrographs and BSE image of various mineral textures in sample 07c. a) Inclusion-poor perthitic K-feldspar in the leucosome (PPL). b) Inclusion-poor perthitic K-feldspar in the leucosome (XPL). c) Rare partially-resorbed garnet grain (PPL). d) Rare partially-resorbed garnet grain (XPL). e) Plagioclase phenocryst in a leucosome lined with a sillimanite and biotite selvedge.

Locality 08

Sampling locality 08 is west-northwest of Hanuman Chatti, where the NH7 bends northwards (30.69918°N, 79.50424°E). The sampled exposure is a 30-50 cm wide band of metapelite bounded by metapsammites (Figure S1.26). The schistose metapelite unit contains bedding- and cleavage-parallel lenticular leucosomes, up to 60 cm across and 5 cm wide.

Sample BAD08 is an *in-situ* migmatite collected from the schistose metapelitic unit, with a fine-grained assemblage of muscovite + quartz + plagioclase + biotite, with minor amounts of K-feldspar + magnetite + garnet + apatite + tourmaline + ilmenite. Zircon and monazite are present as accessory phases. There is only minor chloritization of biotite. There is a sharp transition from schist to leucosome, composed of quartz + plagioclase + muscovite + K-feldspar (Figure S1.27). Quartz grains show undulose extinction throughout the sample.

K-feldspar is present in the schist and leucosome portions of the sample in groups of two or three grains within 3 mm of each other. Grains in the schist portions are rare, 250-600 μm across, and occur in small quartzofeldspathic domains. K-feldspar-muscovite grain boundaries are complex and lined with sericite (Figure S1.28a). In the leucosome, K-feldspar grains are more common and larger, with grains up to 1000 μm in length. Sparse plagioclase myrmekite grains, approximately 200 μm in diameter, are associated with the K-feldspar in both schist and leucosome (Figure S1.28b).

Regular anastomosing laths of muscovite dominate phyllosilicate domains with biotite disseminated evenly throughout. Muscovite also occurs as uncommon elongate strips of

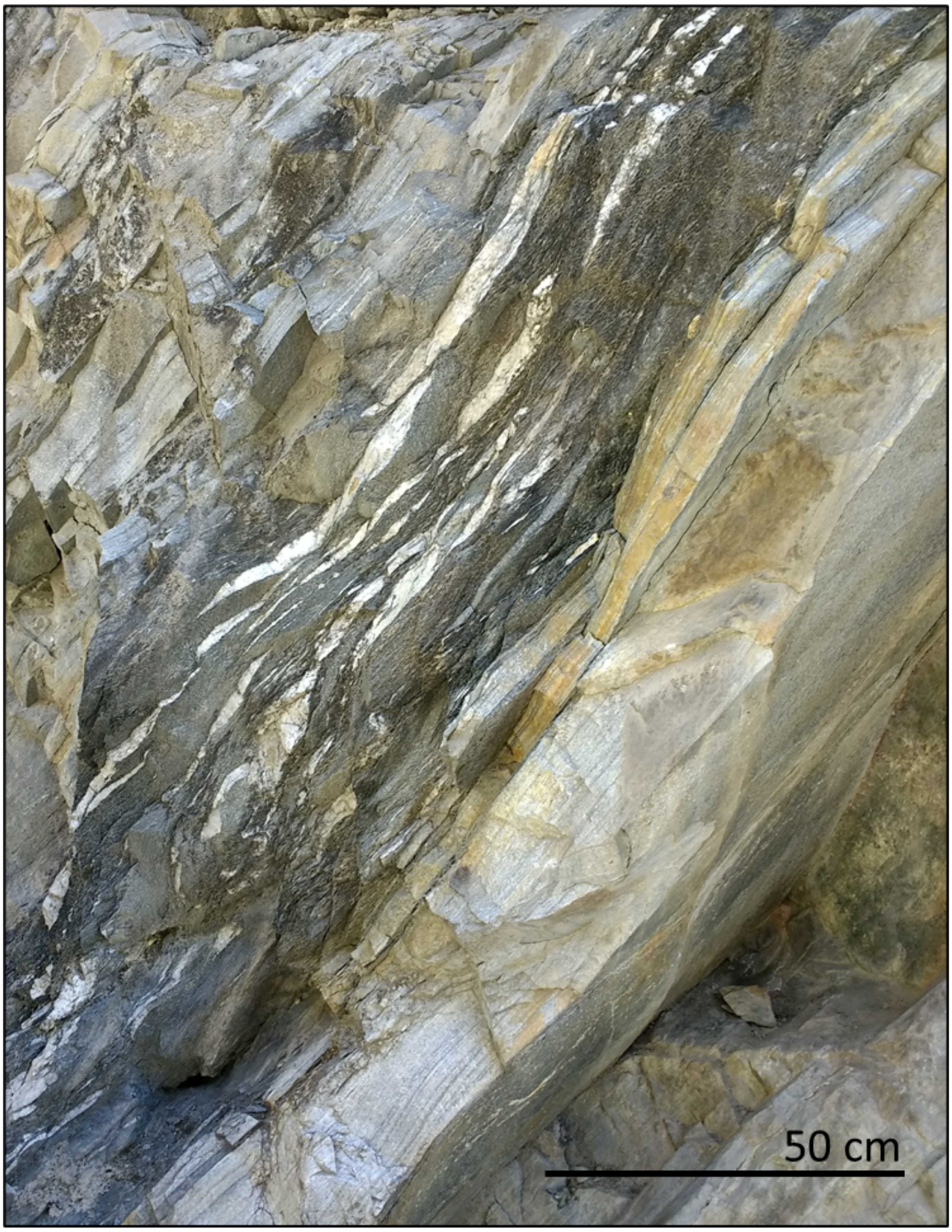

Figure S1.26 – Locality 08 (30.69918°N, 79.50424°E). Sampled metapelitic unit of variable thickness (30-50 cm), with fabric-parallel lenticular leucosomes. On either side of this unit are interbedded metaquartzite and metapsammite units typical of the Pandukeshwar-Badrinath transition.

fine sericite intergrown with biotite and quartz along leucosome boundaries. Garnet occurs in the schist as rare, isolated grains, <500 μm in diameter. They have a distinct pink hue, with irregular grain boundaries and fractures. Tourmaline is present in quartzofeldspathic domains as rare euhedral grains, up to 1800 μm in diameter. These grains are strongly pleochroic with blue-green cores and olive-green rims, which appear black in hand specimen.

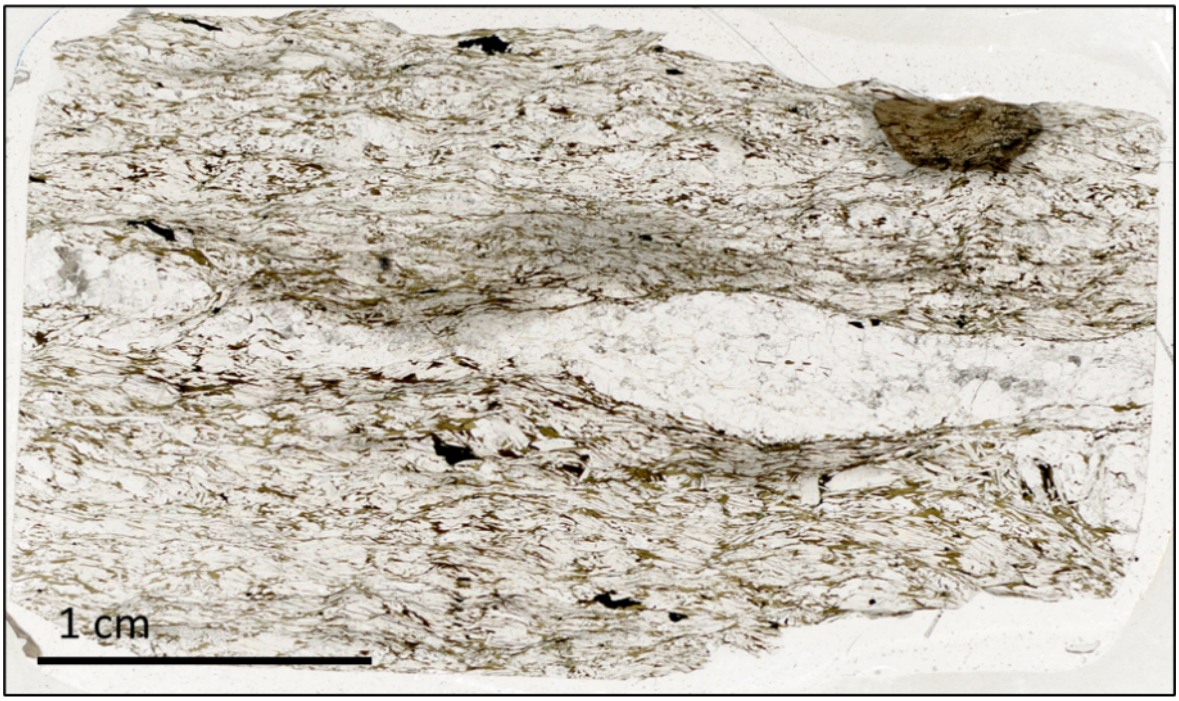

Figure S1.27 – Thin section image of migmatite sample 08, displaying the schistose texture present in the sample and vein-like leucosomes of Qz + Pl + Ms + Kfs.

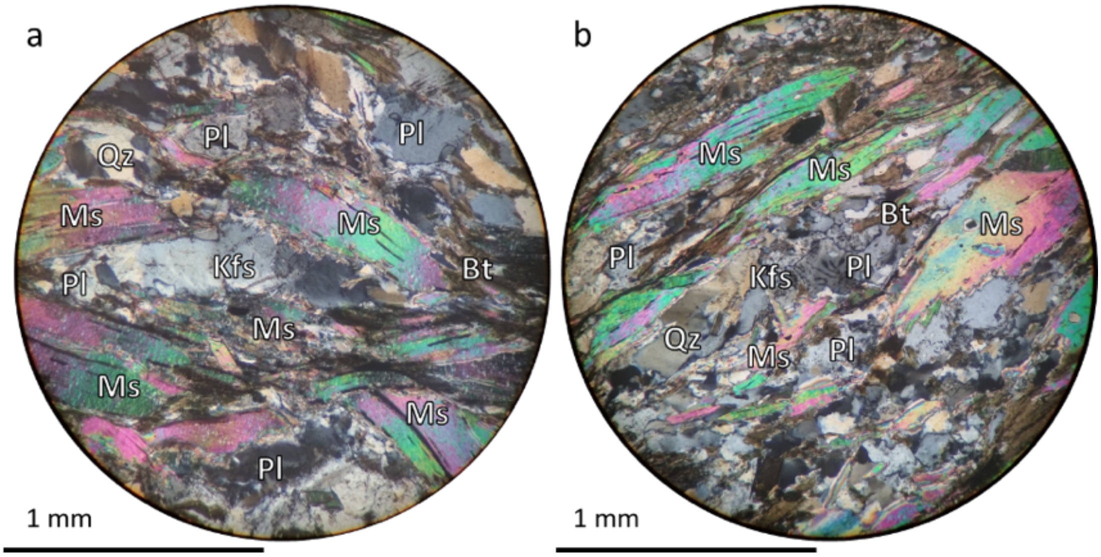

Figure S1.28 – Photomicrographs of feldspar textures in sample 08 (XPL). a) K-feldspar with complex sericitized boundary along contact with muscovite. b) Myrmekitic plagioclase adjacent to K-feldspar.

Locality 09

Sampling locality 09 is 200 m north of locality 08 along the NH7 (30.70085°N, 79.50388°E). The exposure features interbedded metapelites, metapsammites, and metaquartzites; metapelitic beds are less prevalent than at locality 08 and are only 10-20 cm thick. Fabric-parallel leucosomes are present in the metapelitic units, similar to locality 08.

Sample BAD09a is an *in-situ* meta-arkose from a metapelite-metaquartzite unit boundary. This sample is composed of fine to medium-grained quartz + plagioclase + muscovite + biotite + garnet, with minor apatite + ilmenite + tourmaline. Zircon and monazite are present as accessory phases. Thin sections are dominated by quartzofeldspathic domains, which vary from 3-10 mm wide, while phyllosilicate domains range from 0.5-1.0 mm. There is only minor chloritization of biotite. Quartz grains show undulose extinction throughout the sample.

Garnet grains in the phyllosilicate domains are subhedral and up to 1.6 mm in diameter. They are elongated parallel to the fabric and have fractures that run perpendicular to this (Figure S1.29a). Biotite, muscovite, and apatite form pressure shadows. Garnets in the quartzofeldspathic layers are tabular skeletal relics that conform to the fabric (Figure S1.29b). Tourmaline is rare and only occurs in the quartzofeldspathic domains. Grains are <0.6 mm across and strongly pleochroic with olive green colouration.

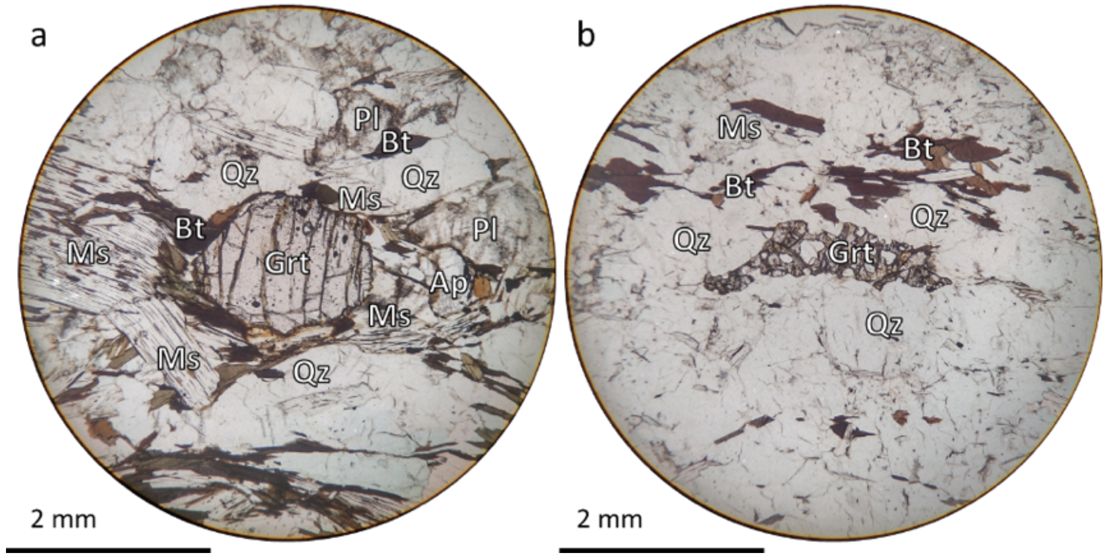

Figure S1.29 – Photomicrographs of garnet grains in sample 09a (PPL). a) Subhedral garnet in phyllosilicate domain with fractures perpendicular to fabric. b) Tabular relict garnet in quartzofeldspathic domain, in-line with fabric.

Sample BAD09b is an *in-situ* migmatite collected from a leucosome-bearing schistose metapelitic unit, with an assemblage of quartz + plagioclase + muscovite + biotite + garnet, with minor ilmenite. Zircon and monazite are present as accessory phases. As with sample BAD08, there is a sharp transition from the schist to the leucosome (Figure S1.30), composed of quartz + plagioclase + muscovite, with a few small biotite grains that have been highly chloritized. Quartz grains show undulose extinction throughout the sample.

Schistose areas are medium-grained, with 4-5 mm wide compositional layering quartzofeldspathic and phyllosilicate domains. The leucosome is coarse-grained, with euhedral primary muscovite. Plagioclase is partially sericitized. The schist-leucosome boundary is lined with medium-grained biotite and muscovite.

Garnet only occurs in the phyllosilicate schist layers and are largely encased in biotite and muscovite. Grains are elongated with the fabric, 1-2 mm long and 0.6-0.8 mm wide, similar to sample BAD09a. They also have annealed fractures perpendicular to the fabric (Figure S1.31) and are rich in quartz inclusions.

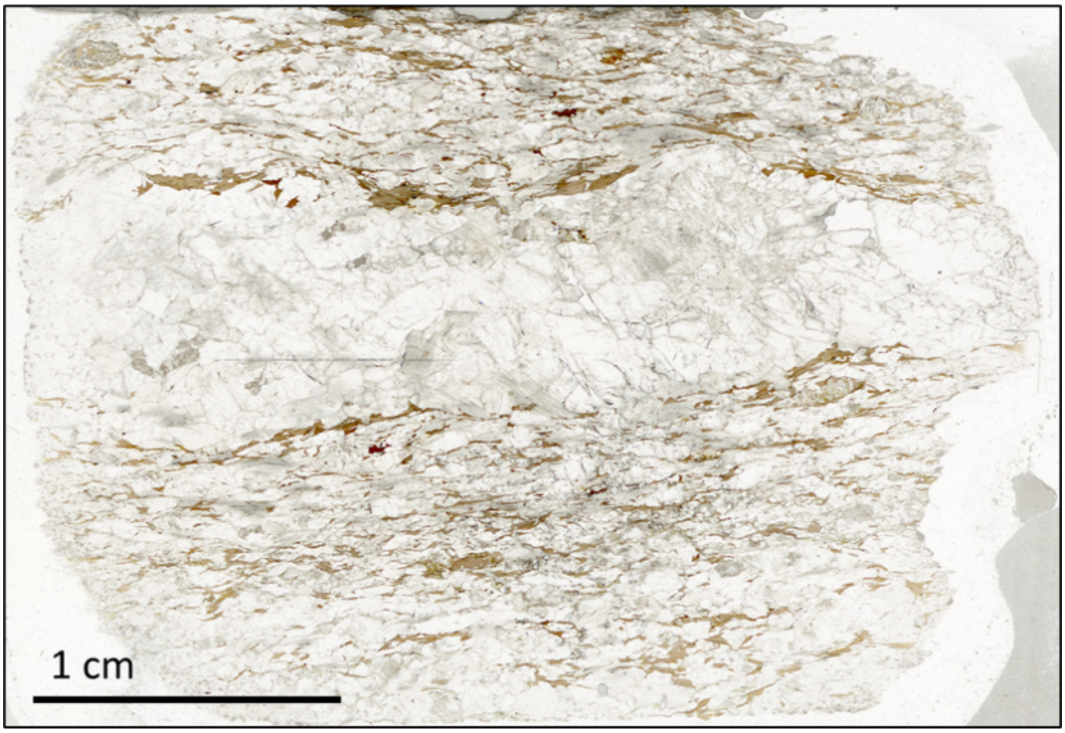

Figure S1.30– Thin section image of migmatite sample 09b, displaying the schistose texture present in the sample and vein-like leucosomes of Qz + Pl + Ms.

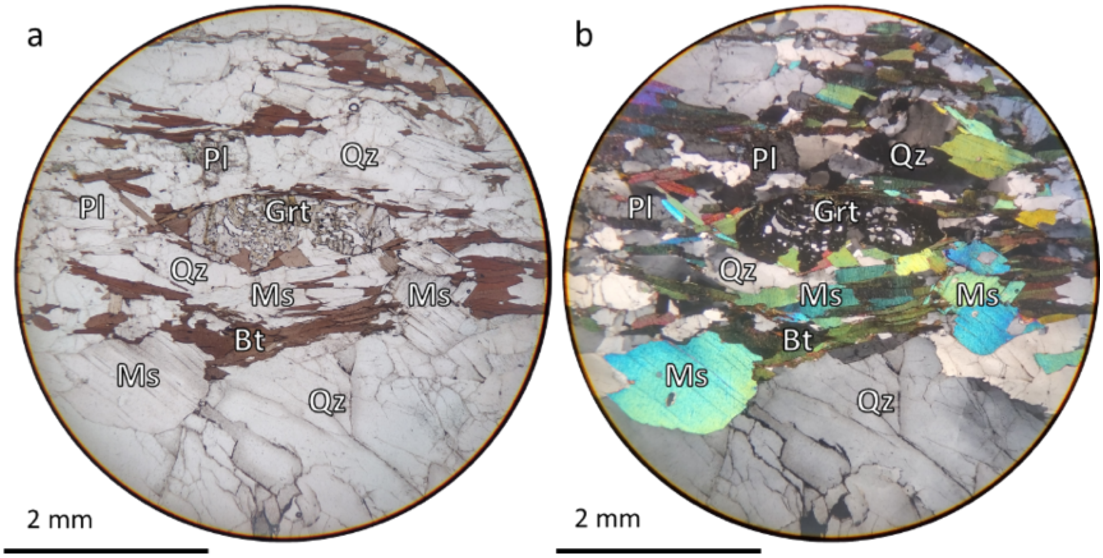

Figure S1.31 – Photomicrographs of a garnet in sample 09b. Garnet is in the phyllosilicate domain, elongated with the fabric and rich in quartz inclusions. Imaged in a) PPL and b) XPL.

Locality 10

Sampling locality 10 is 200m east-southeast of locality 08 along the NH7 (30.69894°N, 79.50621°E). The sample exposure is a biotite-rich gneissose rock with lenticular leucocratic augen.

Sample BAD10 is an *in-situ* medium-grained orthogneiss with an assemblage of biotite + quartz + plagioclase + sillimanite + muscovite + garnet + K-feldspar. Apatite, rutile, zircon, and monazite are present as accessory phases. Quartzofeldspathic domains form asymmetric augen, up to 8 mm long and 4 mm wide, which disrupt phyllosilicate layers (Figure S1.32). Quartz grains show undulose extinction throughout the sample.
K-feldspar is present in two modes, as free grains and as intergrowths in antiperthite. The free grains of K-feldspar are tabular, ranging from 200-400 μm wide and 650-800 μm long,

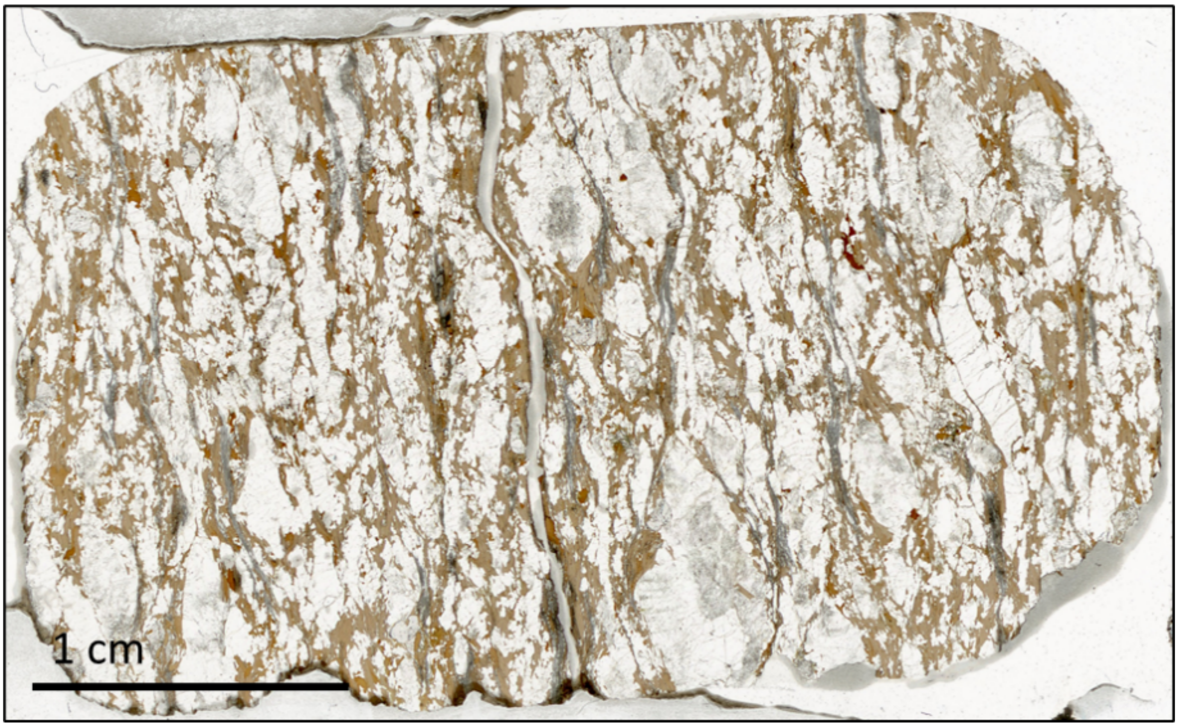

Figure S1.32– Thin section image of orthogneiss sample 10, displaying feldspar augen and anastomosing biotite. Grey-coloured central regions of augen are antiperthitic K-feldspar intergrowths.

with complex sericitized boundaries with micas and contacts with plagioclase that commonly feature myrmekites (Figure S1.33a). Antiperthitic plagioclase intergrowths of K-feldspar form a network of irregular tabs up to 350 μm. This texture is only present in coarser plagioclase grains (> 1.5 mm) (Figure S1.33b).

Sillimanite is in the form of fibrolite clots with fine-grained muscovite intergrowths, up to 5.5 mm long and 200 μm wide within biotite-rich layers. Kyanite is also present as rare tabular grains, 150-500 μm long, either enclosed in plagioclase or at plagioclase-biotite grain boundaries (Figure S1.33). Garnet either forms subhedral grains ~1 mm in diameter with quartz inclusion-rich cores, or as skeletal relics with irregular replacement by quartz-biotite-rutile aggregates.

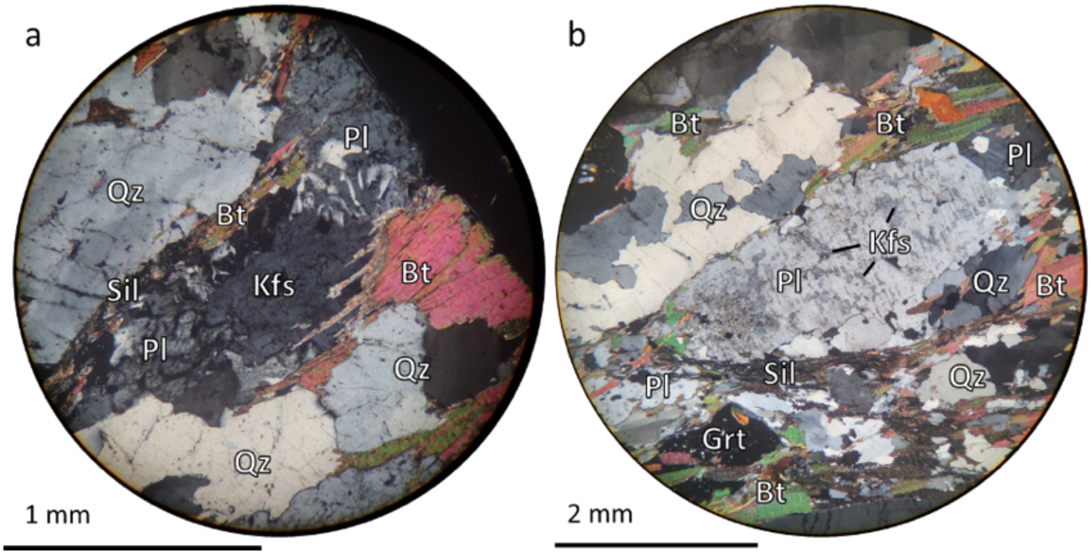

Figure S1.33 – Photomicrographs of feldspar textures in sample 10. a) Fine, tabular K-feldspar grain and myrmekitic plagioclase (XPL). b) Coarse plagioclase grain with antiperthitic K-feldspar intergrowths.

Locality 11

Sampling locality 11 is to the north of Joshimath, across the Alaknanda River (30.56328°N, 79.56776°E), in the Joshimath Formation of the GHS. Samples were taken from >3 m diameter blocks along a section of road cutting. The blocks are a biotite-rich migmatite with irregular, folded leucosome up to 0.8 cm wide and garnets 2-5 mm in diameter.

Sample 11a is an *in-situ* metatexite from locality 11, with an assemblage of quartz + plagioclase + biotite + muscovite + garnet + tourmaline. Apatite, ilmenite, and zircon are present as accessory phases. The sample largely consists of medium-grained mesosome. Quartzofeldspathic domains, however, form highly irregular coarse-grained leucosomes bound by biotite-dominated phyllosilicate bands. There is little chloritization, and quartz grains show undulose extinction throughout the sample.
In the leucosomes, plagioclase grains are typically 1.2-3.2 mm across, with albite twinning common. Some grains up to 6.8 mm with inclusions of muscovite, quartz, and biotite (Figure S1.34a-b). Plagioclase in the mesosome forms grains 0.6-1.2 mm across, commonly with albite twinning, and is less abundant than in the leucosomes. Plagioclase shows partial sericitization in both domains.

Biotite forms fine irregular laths in the mesosome, typically 0.5-0.8 mm long, while in phyllosilicate domains, it forms medium laths 1.0-1.4 mm long with rare grains >2 mm. Chlorite is associated with sericite alteration and the grain boundaries of garnets. Muscovite grains in the mesosome form fine 0.3-0.9 mm laths with irregular boundaries with quartz and plagioclase. In phyllosilicate domains, muscovite forms 1.3-2.8 mm laths with irregular grain boundaries with leucosome plagioclase. Muscovite is also present towards the edges of leucosomes as rare muscovite/sericite clots between plagioclase grains with an aspect ratio of ~10:1 (Figure S1.34c-d).

Garnet is present in phyllosilicate domains as euhedral, pale pink grains, 2.1-4.5 mm in diameter, with quartz inclusions and atoll textures. Anhedral relict grains are also present in the mesosome, up to 850 x 350 µm, again with quartz inclusions. Tourmaline is also associated with the phyllosilicate domains and forms pleochroic euhedral grains 0.8-3.2 mm in cross-section, with an olive-green colouration.

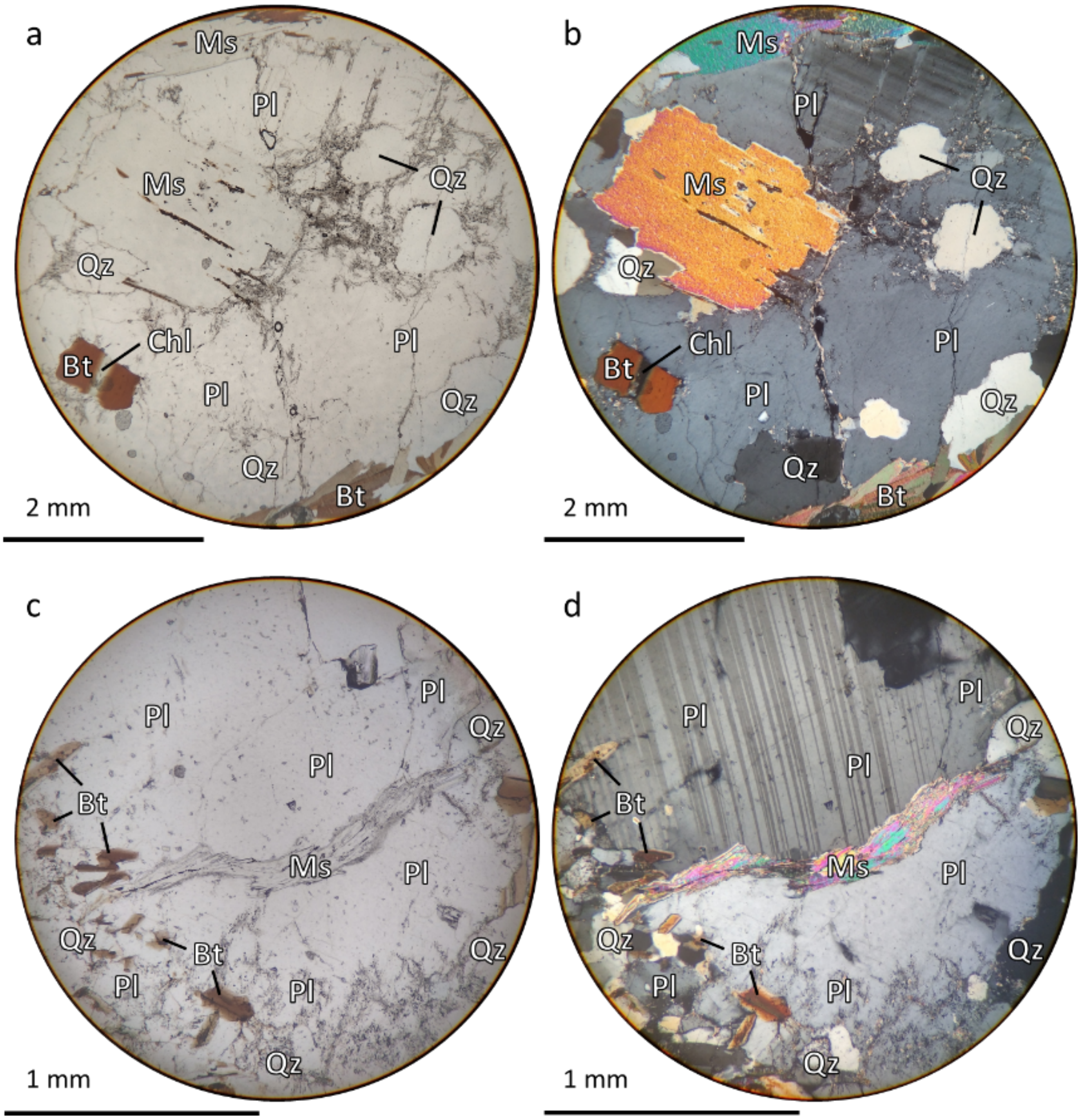

Figure S1.34 – Photomicrographs of plagioclase and muscovite textures in sample 11a. a) Coarse-grained plagioclase in the leucosome, with inclusions of Qz + Ms + Bt (PPL). b) Coarse-grained plagioclase in the leucosome, with inclusions of Qz + Ms + Bt (XPL). c) Muscovite-sericite clot between plagioclase grains in the leucosome (PPL). d) Muscovite-sericite clot between plagioclase grains in the leucosome (XPL).

Float Locality A

Sampling locality A is 0.3 km south of locality 02, 1.8 km west of Badrinath (30.74142°N, 79.47026°E). Float samples were collected at this locality from a broad rocky bar in the Rishi Ganga stream. The catchment of these samples is relatively well-constrained due to the encircled nature of the valley.

Sample f02 is a medium-grained diatexite with coarse-grained leucosomes. It consists of quartz + muscovite + plagioclase + K-feldspar + tourmaline + apatite + biotite + garnet, with magnetite, zircon, and monazite present as accessory phases. The mesosome is rich in muscovite with K-feldspar-quartz-muscovite clusters and minor biotite, while the leucosome mostly lacks phyllosilicates, with a composition of quartz + plagioclase + tourmaline + K-feldspar + sericite (Figure S1.35). Quartz grains show undulose extinction throughout the sample.

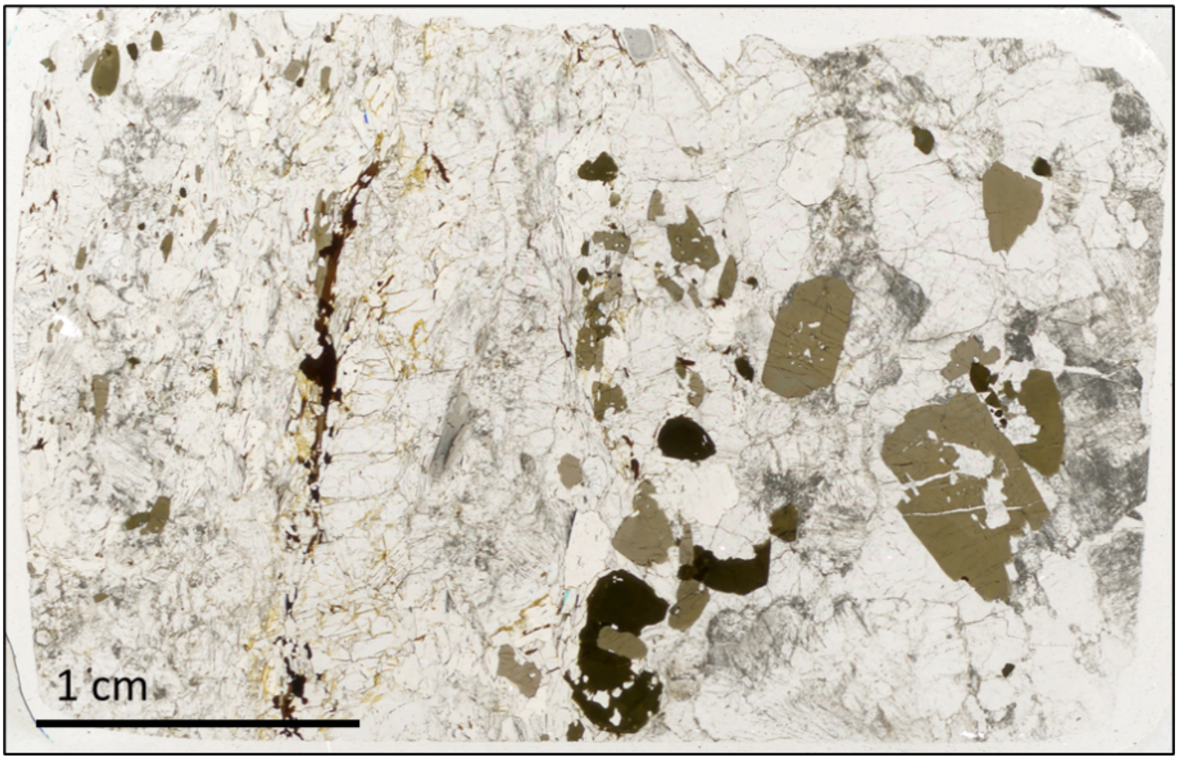

S1.35 – Thin section image of migmatite sample f02, displaying medium-grained mesosome (left) and coarse-grained leucosome with pleochroic tourmaline (right).

In the mesosome, K-feldspar grains, typically 0.8-1.4 mm, form loose clusters with quartz and muscovite, with rare plagioclase grains with K-feldspar overgrowth (Figure S1.36a). Plagioclase grains are 0.5-1.6 mm, with partial sericitization and scattered myrmekitic grains along K-feldspar contacts. In the leucosome, K-feldspar forms medium perthitic grains, 3.3-4.5 mm, with inclusions of quartz and plagioclase, and also as groups of finer grains of disaggregated K-feldspar clusters from the mesosome. Plagioclase forms medium-coarse grains, 3.2-8.3 mm across, the largest showing antiperthitic blebs of K-feldspar.

Muscovite is present as fibrous sericite clots along the mesosome-leucosome boundary, up to 3.4 mm long. In the mesosome proper, muscovite is present either as 500-750 μm

grains in the K-feldspar-quartz-muscovite clusters or as medium-grained laths, typically 1.6 mm long, in phyllosilicate bands, which are host to apatite and tourmaline. Biotite is only present in the mesosome as 0.4-1.5 mm laths, which have syntaxial overgrowth of muscovite (Figure S1.36b). Tourmaline forms euhedral prismatic grains up to 6.4 mm in the leucosome and up to 1.6 mm in the mesosome, associated with the phyllosilicate bands. Tourmaline is strongly pleochroic, with blue-green cores and olive-green rims. Garnet grains are rare, less than 600 μm in diameter, with irregular boundaries and associated with fine chlorite.

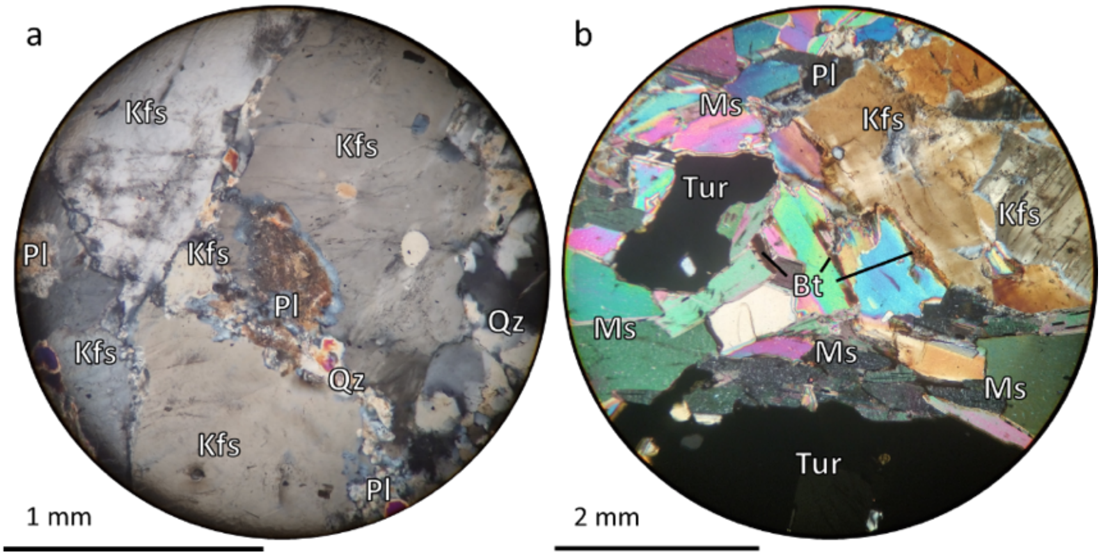

Figure S1.36 – Photomicrographs of feldspar and mica textures in sample f02. a) Plagioclase with K-feldspar overgrowth/replacement in the mesosome (XPL). b) Irregular biotite laths optically continuous with muscovite in phyllosilicate-rich domains (XPL).

Sample f03 is a medium-grained orthogneiss, consisting of K-feldspar + quartz + biotite + plagioclase + sillimanite + muscovite, with minor garnet + apatite. Zircon and monazite are present as accessory phases. Quartzofeldspathic domains form irregular augen, up to 20 mm long and 16 mm wide, but typically 12 x 5 mm, which disrupt phyllosilicate layers. Quartz grains show undulose extinction throughout the sample.

K-feldspar grains are typically rounded, 0.8-1.5 mm across, with either crosshatch twinning or very fine perthitic albite. Plagioclase grains are between 0.8-1.2 mm, with albite twinning. Myrmekitic plagioclase is common along K-feldspar contacts (Figure S1.37). Both feldspars are partially sericitized.

Phyllosilicate domains primarily consist of interlocking biotite laths, 0.8-1.4 mm long, which are host to garnets, <200 μm in diameter, and apatite, <350 μm in diameter. Muscovite grains are 0.5—1.2 mm long, with irregular boundaries along contacts with K-feldspar. Pockmarked grains occur in quartzofeldspathic domains, intergrown with K-feldspar (Figure 2.26). Sillimanite forms fibrolite clots with fine-grained muscovite intergrowths up to 5.0 mm long and 0.6 mm wide within phyllosilicate domains.

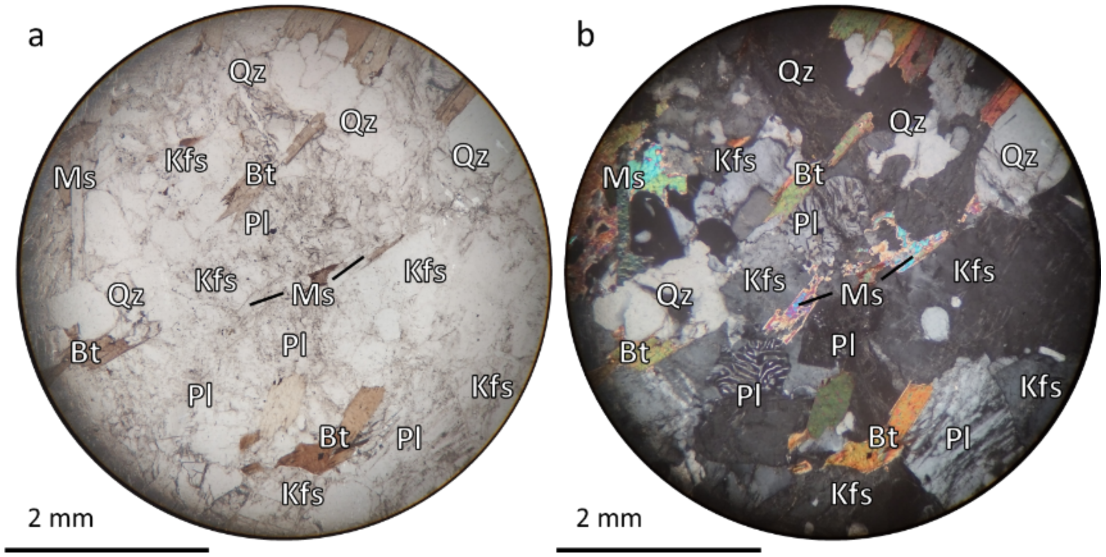

Figure S1.37 – Photomicrographs of feldspar and mica textures in sample f03. Myrmekitic plagioclase in contact with K-feldspar grain and pockmarked muscovite with irregular grain boundaries. Imaged in a) PPL, and b) XPL.

Sample f04 is a medium-grained leucogranite with an assemblage of plagioclase + quartz + K-feldspar + muscovite + biotite + tourmaline, with minor sillimanite. Apatite, zircon, and monazite are present as accessory phases. Biotite forms disaggregated schlieren strands with muscovite and sillimanite. Only a few biotite grains are affected by chloritization, constrained to the edges of biotite grains. Quartz grains show undulose extinction throughout the sample.

K-feldspar forms grains, typically 1.8-5.0 mm across, with perthitic albite exsolution lamellae (Figure S1.38e). Crosshatch twinning is common. Grains may have poikiloblastic zones with quartz and muscovite inclusions. Partial sericitization is prevalent around exsolution textures. Plagioclase forms 1.5-5.6 mm grains, with rare grains up to 12 mm, which are strongly sericitized. Boundaries with K-feldspar are irregular, with myrmekitic plagioclase, sericite, and quartz (Figure S1.38a-b).

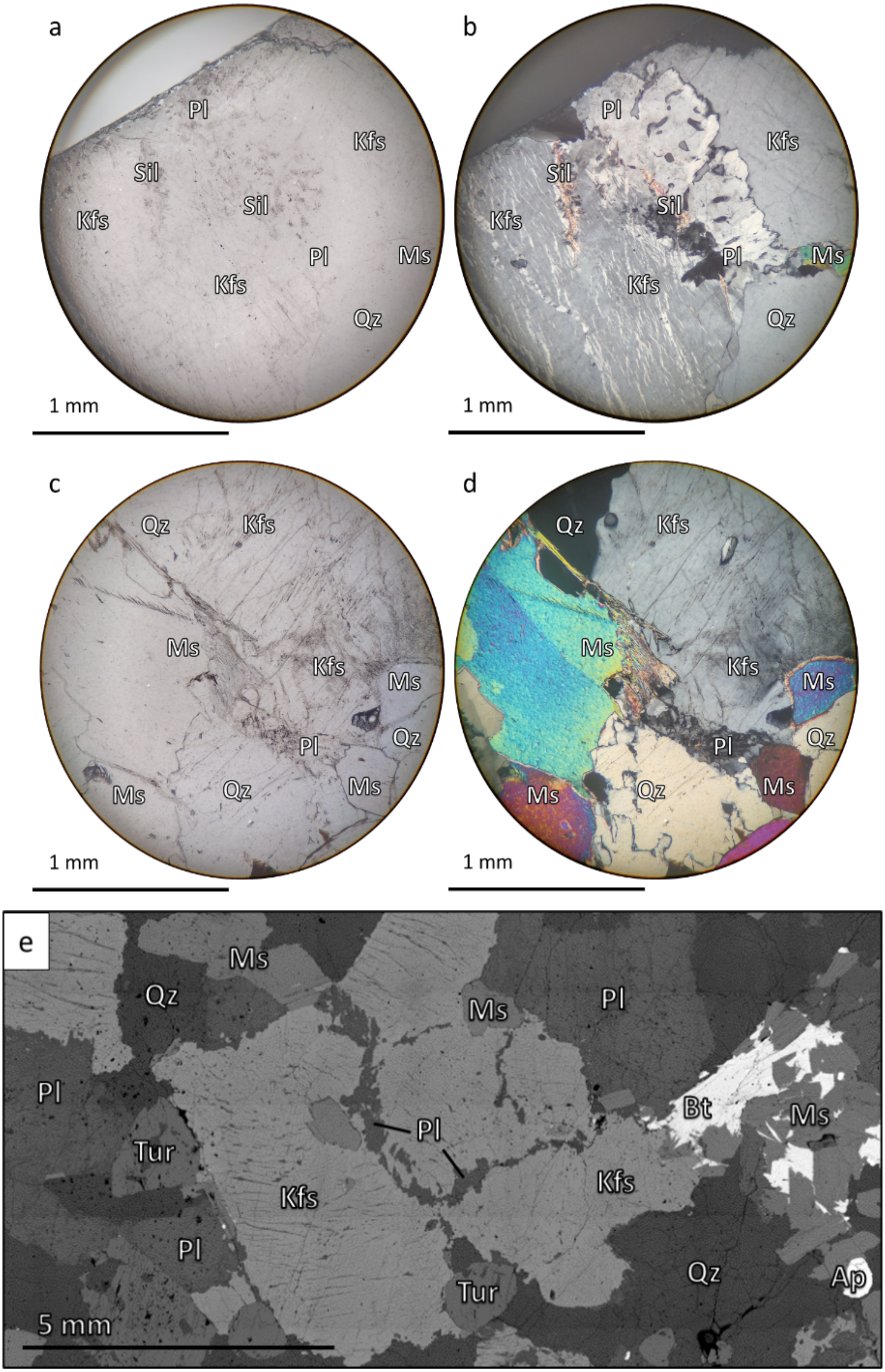

Figure S1.38 – Photomicrographs and BSE image of various mineral textures in sample f04. a) Myrmekitic plagioclase between perthitic K-feldspar (PPL). b) Myrmekitic plagioclase between perthitic K-feldspar (XPL). c) Muscovite with sericite and irregular grain boundaries with K-feldspar (PPL). d) Muscovite with sericite and irregular grain boundaries with K-feldspar (XPL). e) BSE image of K-feldspar porphyroblasts (light grey) and irregular plagioclase inclusions.

Muscovite is present either as fine laths, typically 0.6 mm across, interlocking with biotite in the strands of schlieren, or as subhedral medium grains, 0.9-2.9 mm across. Muscovite grain boundaries with K-feldspar are irregular, with interstitial sericite common (Figure S1.38c-d). Biotite only occurs as fine-medium grains, 0.4-2.5 mm across, in the schlieren, with irregular schlieren-external boundaries. Sillimanite is also only present in the schlieren as remnants of fibrolite clots within fine-grained muscovite. Tourmaline forms euhedral prismatic grains, 1.0-3.2 mm across in cross-section, that are strongly pleochroic with blue-green cores (not always present) and olive-green rims.

Float Locality B

Sampling locality B is 0.3 km southeast of locality 04, 3.6 km west of Badrinath (30.74233°N, 79.45197°E). Here, float samples were collected from an alluvial fan at the base of a steep gully on the valley's north cliff face. As such, the catchment of these samples is well-constrained.

Sample f13 is a medium-grained schollen diatexite with a coarse-grained leucosome. The schollen mesosome consists of quartz + plagioclase + muscovite + biotite + K-feldspar, while the leucosome consists of quartz + plagioclase + K-felspar + tourmaline + muscovite (Figure S1.39). Apatite, zircon, and monazite are present as accessory phases in both domains. Biotite is rarely chloritized but constrained to the edges of grains where present. Quartz grains show undulose extinction throughout the sample.
In the leucosome, K-feldspar is present either as irregular strongly sericitized grains, 0.6-1.0 mm across, with crosshatch twinning and perthitic albite exsolution or as coarser 3.8-

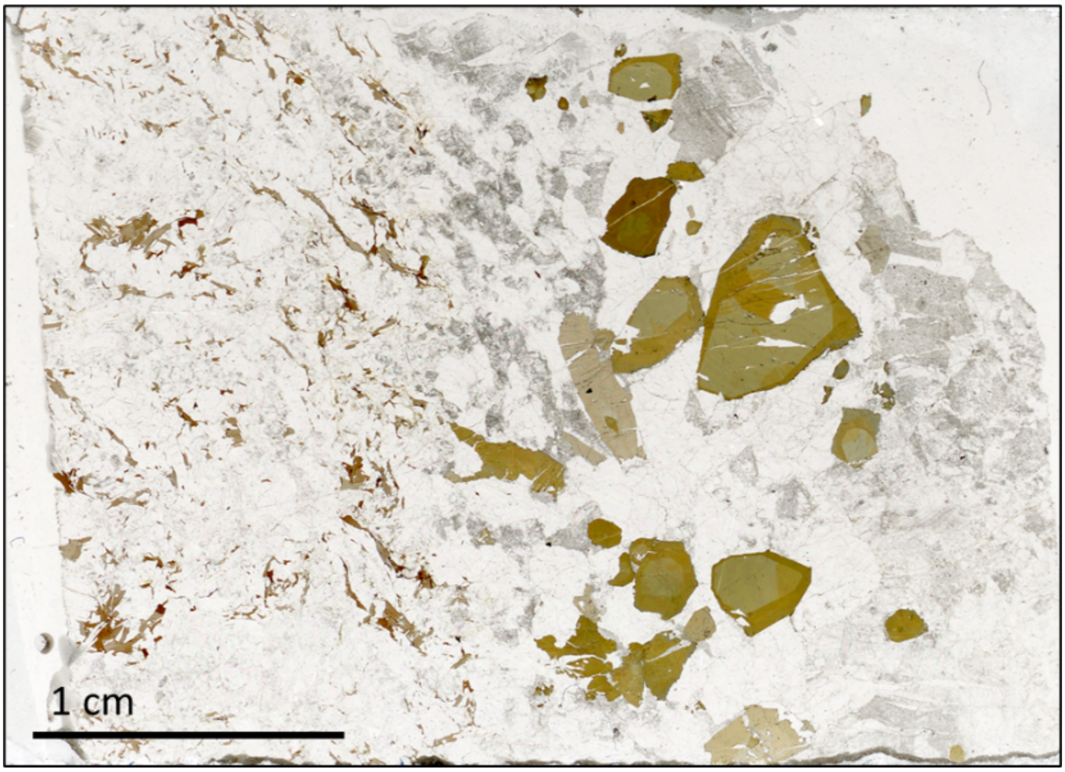

Figure S1.39 – Thin section image of migmatite sample f13, displaying medium-grained mesosome with biotite (left) and coarse-grained leucosome with pleochroic tourmaline (right).

6.0 mm perthitic porphyroblasts with poikiloblastic quartz, muscovite with quartz films, and plagioclase inclusions (Figure S1.40a-b). Coarse perthite grains show partial sericitization, with myrmekitic plagioclase and sericitized muscovite along grain boundaries. Plagioclase forms 0.8-1.4 mm grains close to the mesosome-leucosome transition and sub-euhedral 2.0-6.6 mm grains further within the leucosome. Both display albite twinning and are strongly sericitized. In the mesosome, K-feldspar grains, 0.6-1.1 mm across, form irregular clusters with plagioclase and quartz up to 3.5 mm wide. Grains are partially sericitized, and myrmekitic plagioclase is common along grain boundaries. Plagioclase grains are typically 1 mm across, with albite twinning and partial sericitization.

Muscovite forms 0.8-4.0 mm laths in the leucosome, with irregular grain boundaries along plagioclase and K-feldspar, as well as sericite clots up to 3.5 mm long and 1 mm wide (Figure S1.40c-d). In the mesosome, muscovite laths are typically smaller, 0.6-1.9 mm across, also with irregular and sericitized grain boundaries with feldspars, and form sericite strands up to 4.2 mm long and <200 μm wide. Biotite is only present in the mesosome, with 0.3-0.8 mm laths with increasingly irregular grain boundaries towards the mesosome-leucosome transition. Tourmaline is present only in the leucosome, with widely variable prismatic grain sizes from 0.4 to 5.8 mm in cross-section, strong pleochroism, blue-green coloured cores and brown-green rims. Larger grains show further zoned colouration with an additional blue-green to brown-green rim overgrowth.

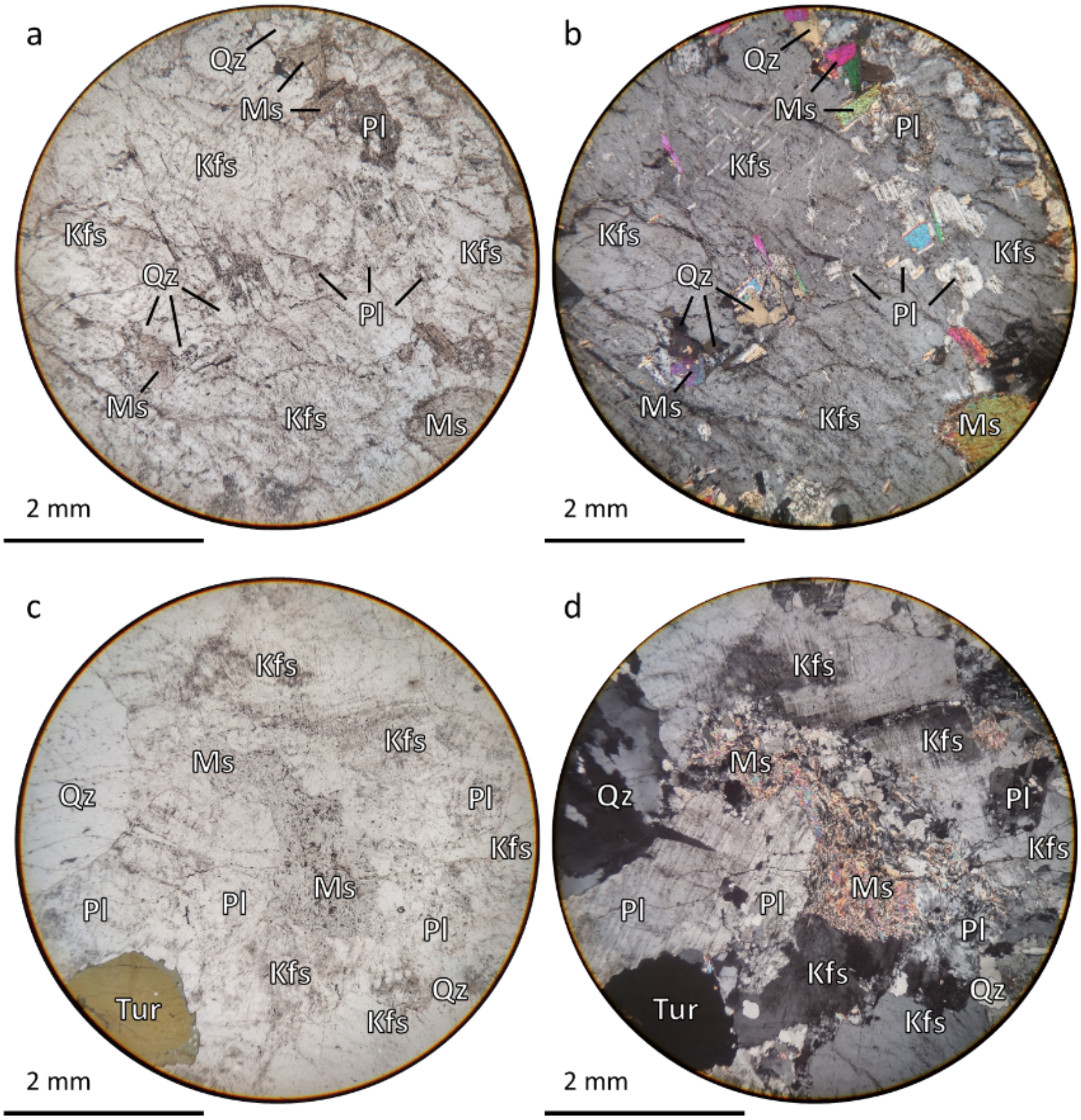

Figure S1.40– Photomicrographs of feldspar and sericite textures in sample f13. a) Poikiloblastic K-feldspar, with inclusions of quartz, plagioclase, and muscovite with quartz films (PPL). b) Poikiloblastic K-feldspar, with inclusions of quartz, plagioclase, and muscovite with quartz films (XPL). c) Sericite clot between feldspar grains in the leucosome (PPL). d) Sericite clot between feldspar grains in the leucosome (XPL).

Online Resources 3

Zircon and monazite spot analysis locations

Part 1 Zircon

CL images of zircon grains from each sample analysed with U-Th-Pb ages relevant to the Himalayan orogen. Red dashed circles mark the trace element concentration laser ablation pit (33 µm) on top of the previous U-Th-Pb analytical sites. Where present, blue dashed circles mark U-Th-Pb analytical sites in cases where trace elements were measured at a separate site or not at all.

Sample 01d

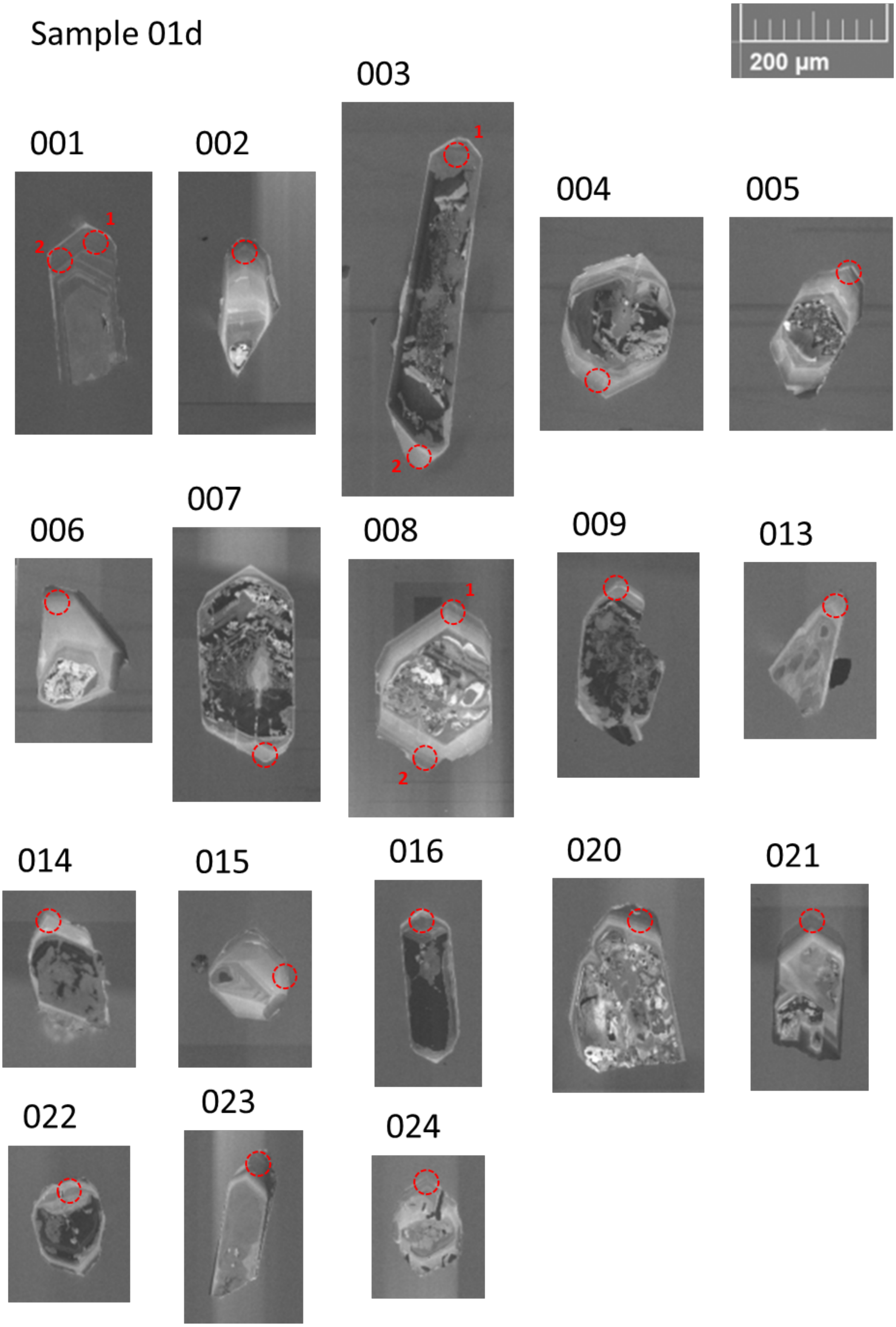
200 µm
001
1
2
002
003
1
2
004
005
006
007
008
1
2
009
013
014
015
016
020
021
022
023
024

Sample 02a

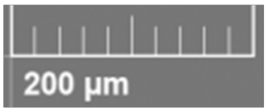

001

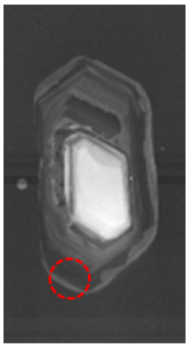

003

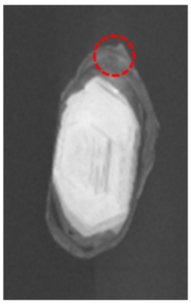

004

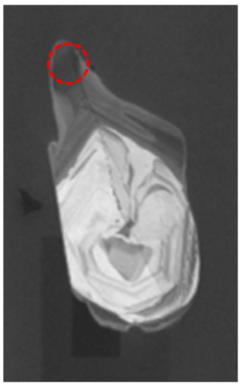

006

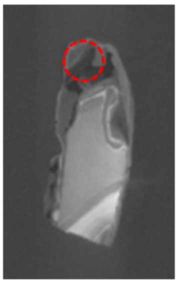

007

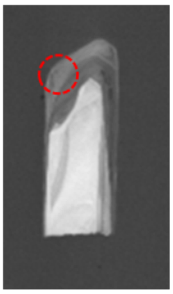

018

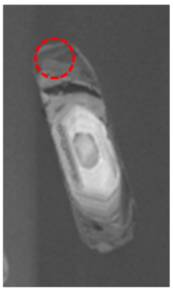

020

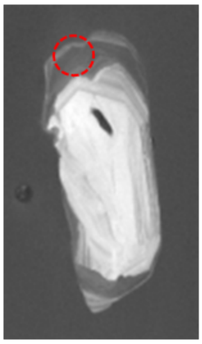

028

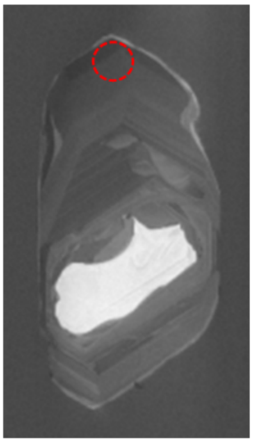

030

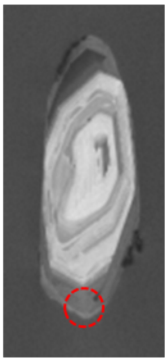

035

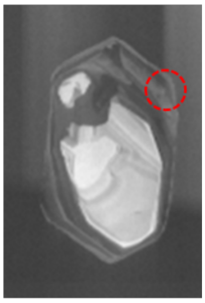

038

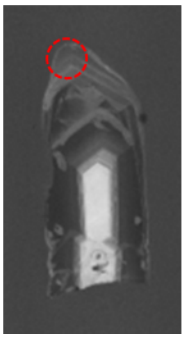

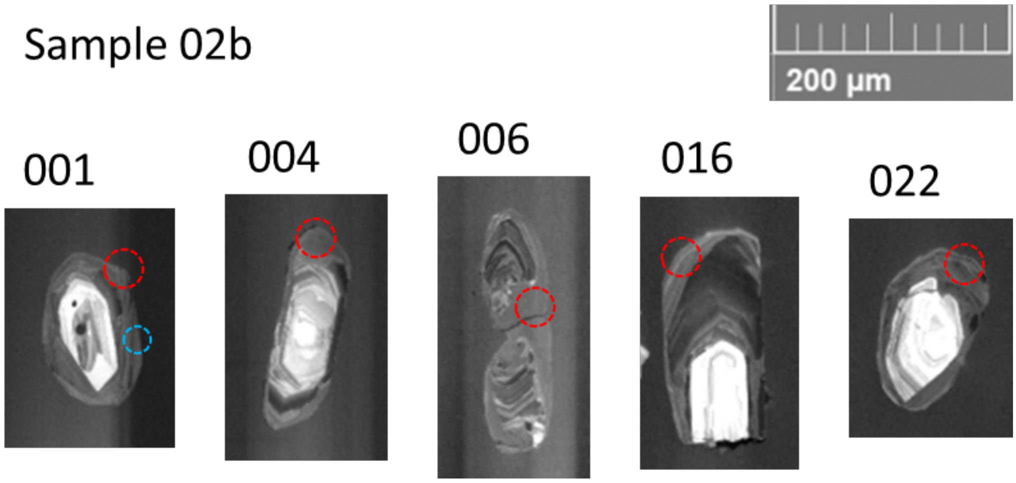
Sample 02b
200 µm
001
004
006
016
022

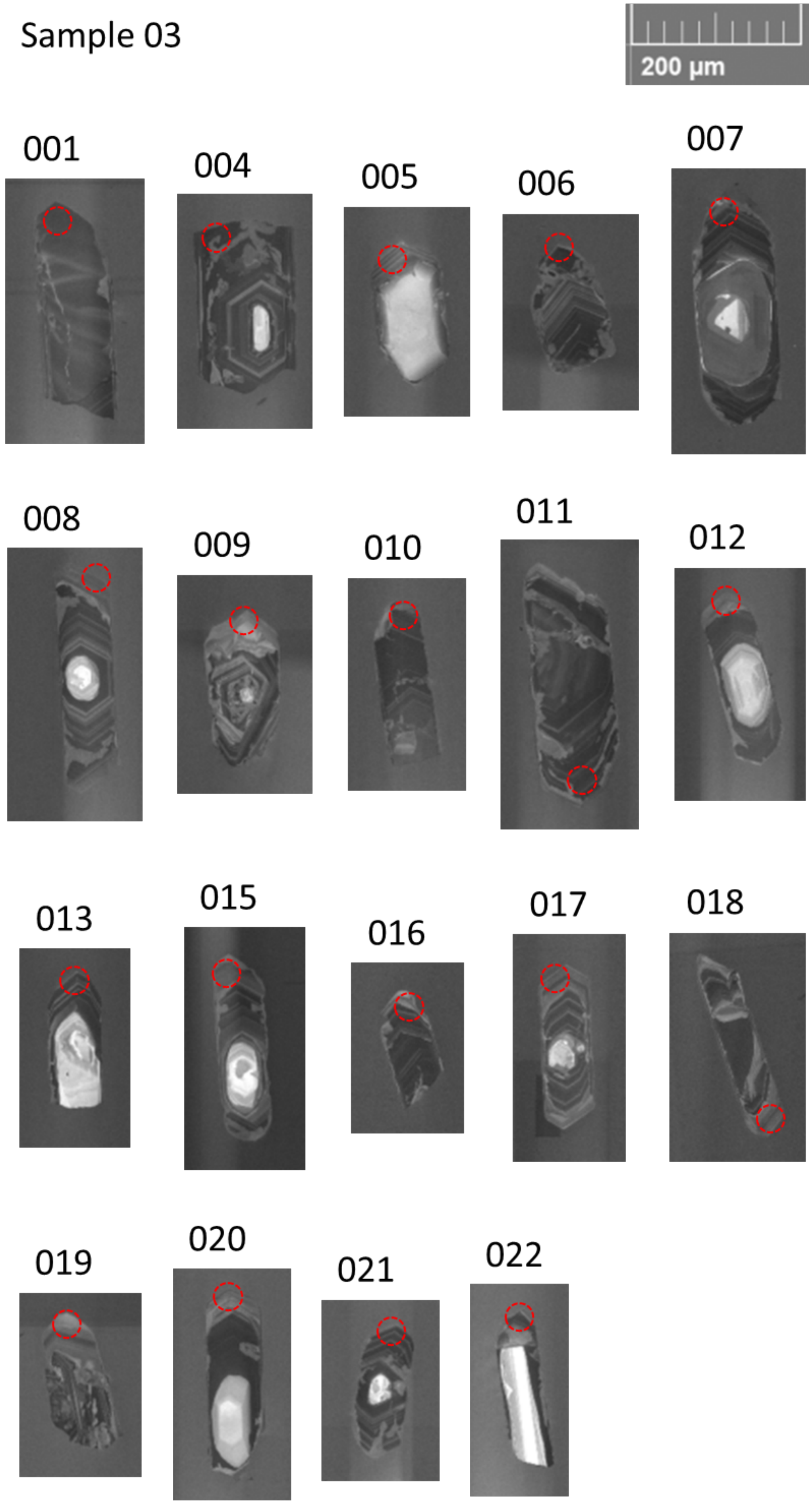
Sample 03
200 µm
001
004
005
006
007
008
009
010
011
012
013
015
016
017
018
019
020
021
022

Sample 04a

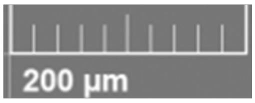

001

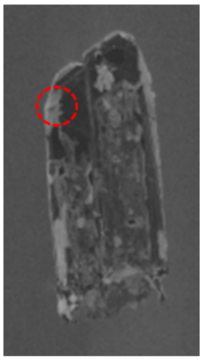

005

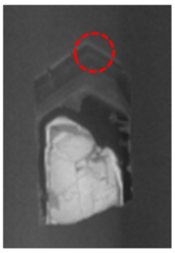

010

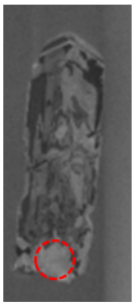

013

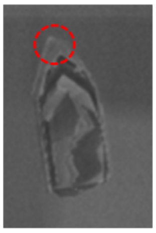

014

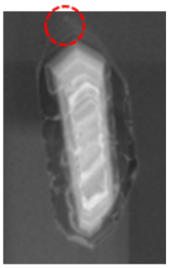

025

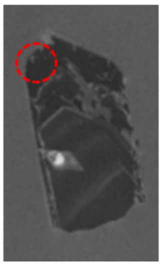

Sample 04b

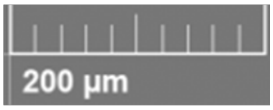

013

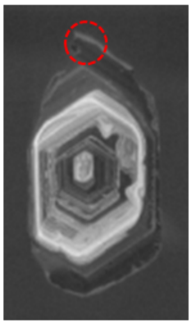

015

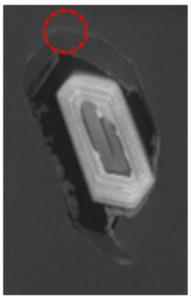

027

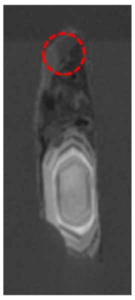

029

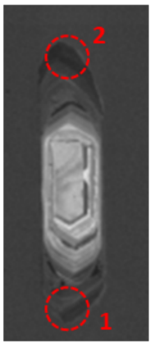

Sample 06

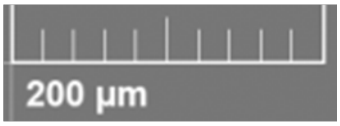

005

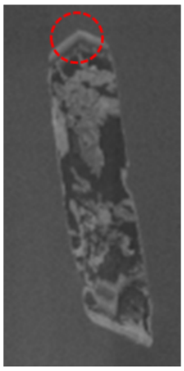

006

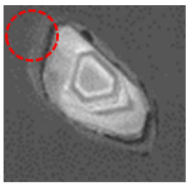

007

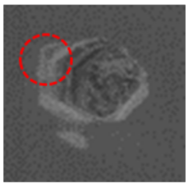

Sample 07a

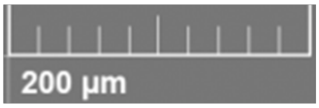

002

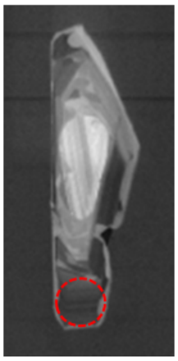

019

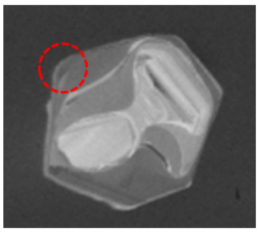

Sample 07b

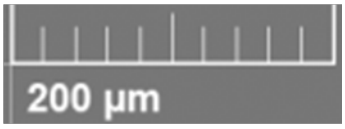

012

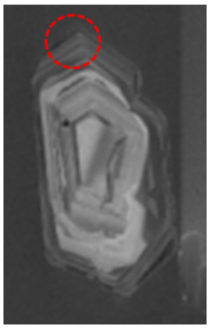

018

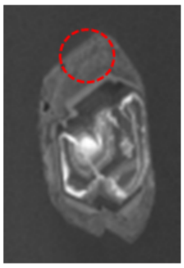

026

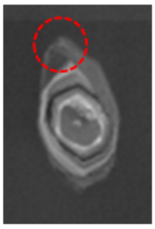

028

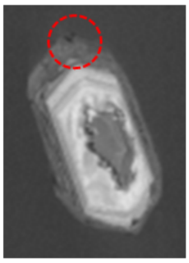

029

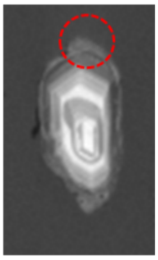

Sample 07c

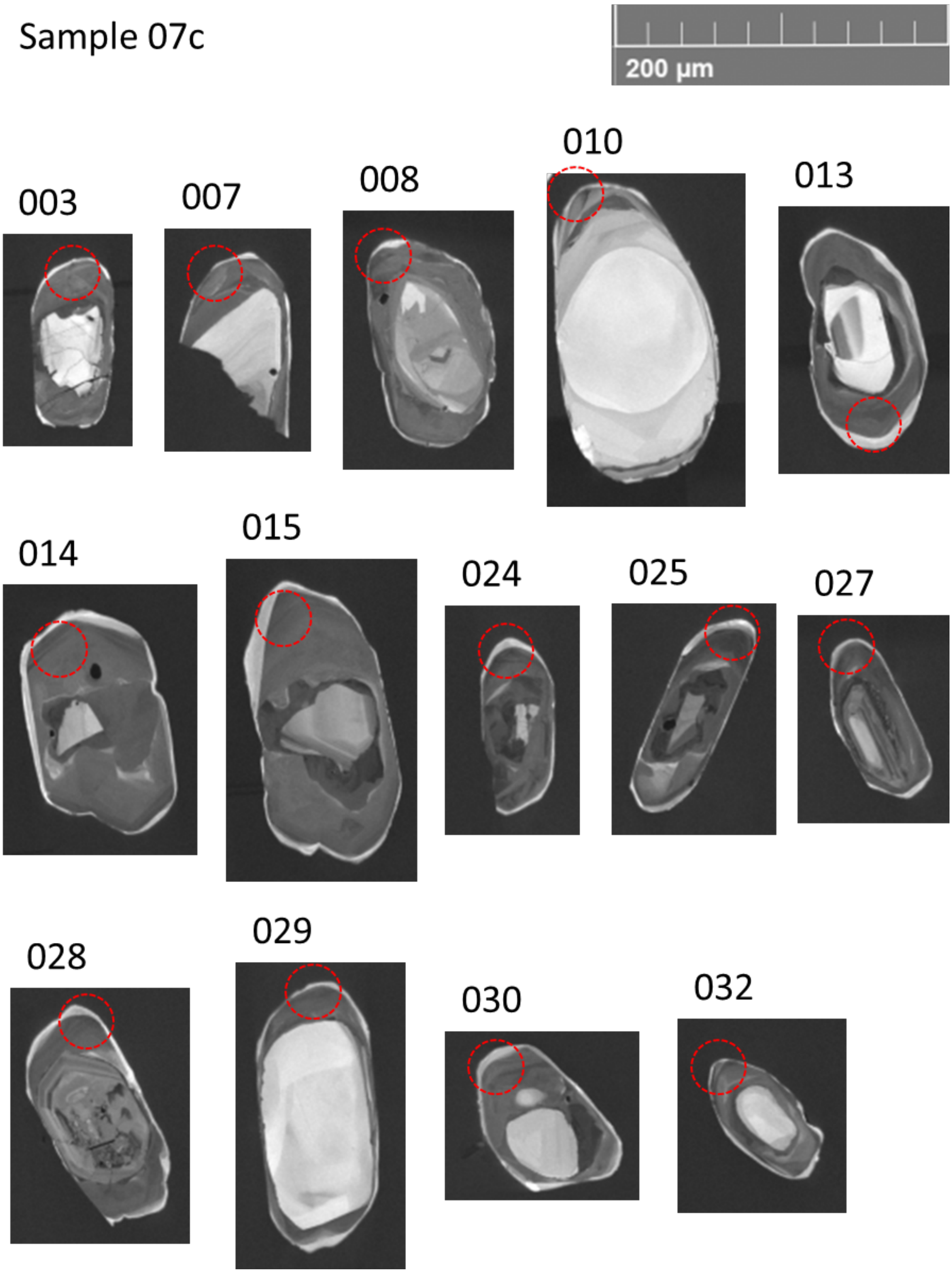

Sample 09b

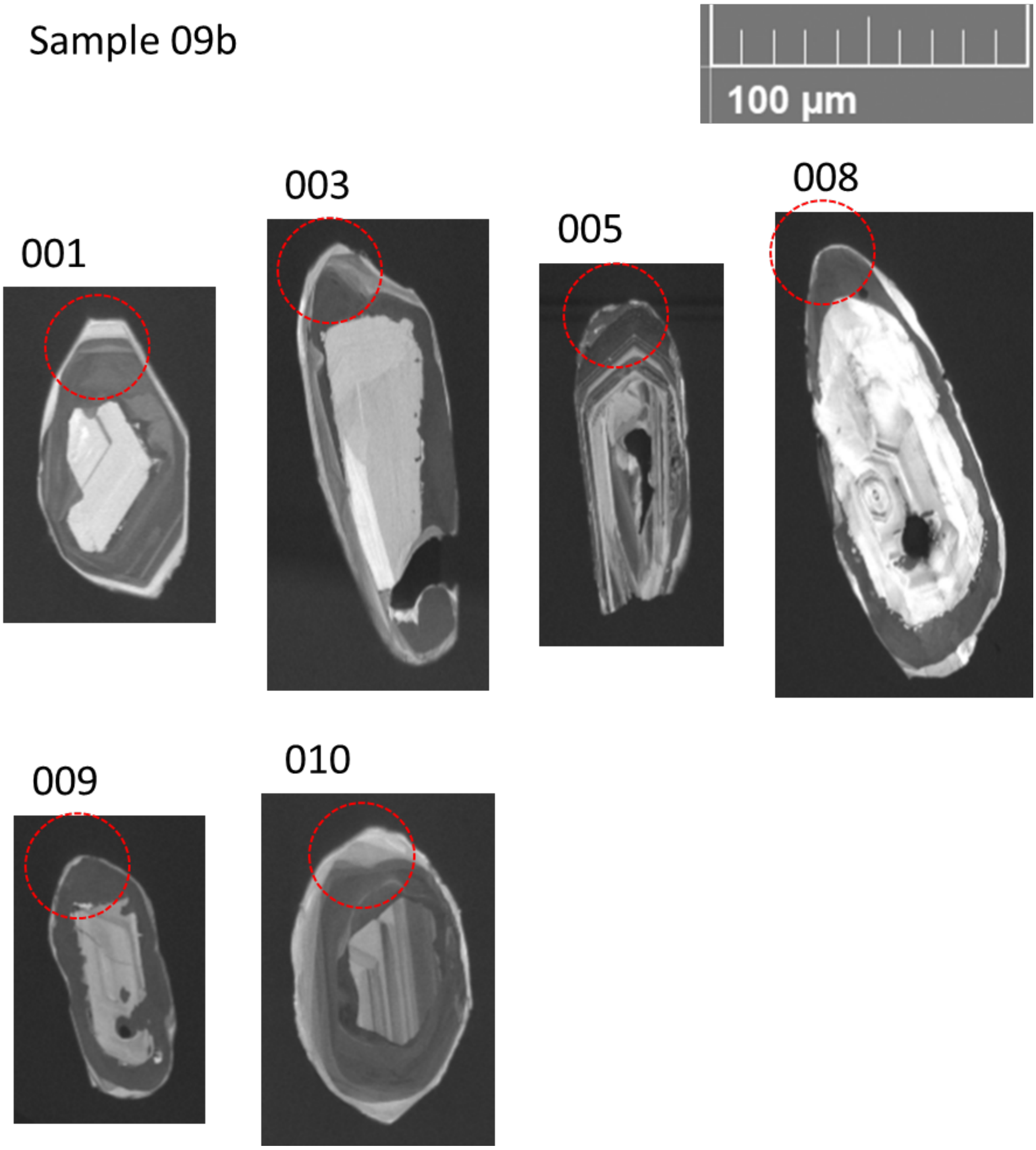

Sample 11a

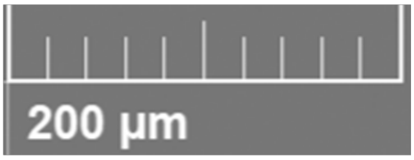

005

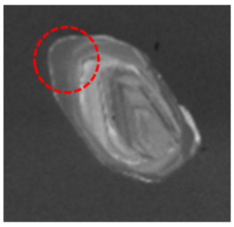

006

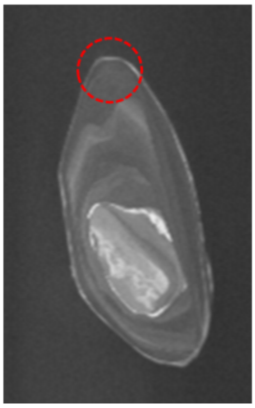

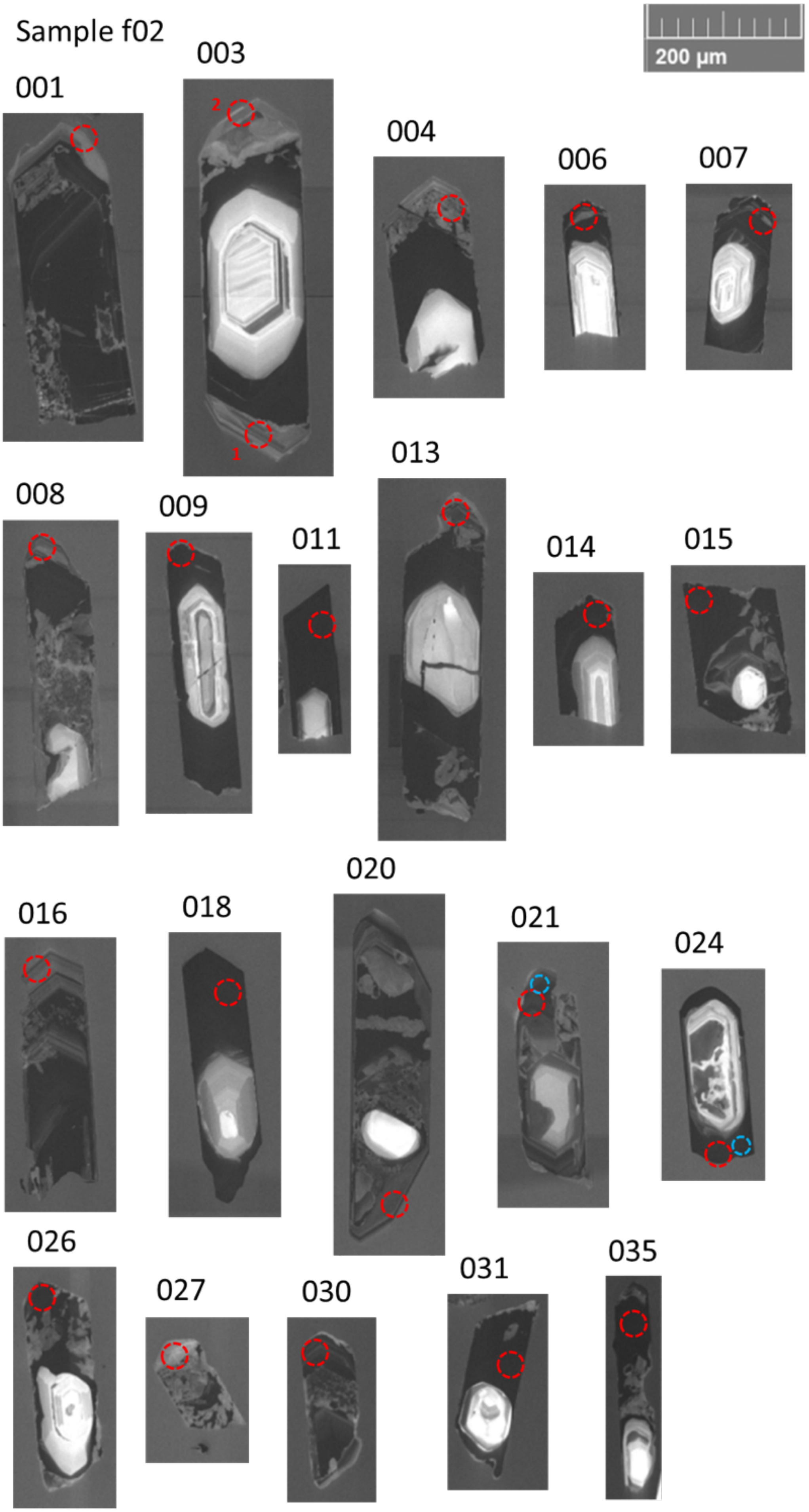

Sample f02
200 µm
001
003
2
1
004
006
007
008
009
011
013
014
015
016
018
020
021
024
026
027
030
031
035

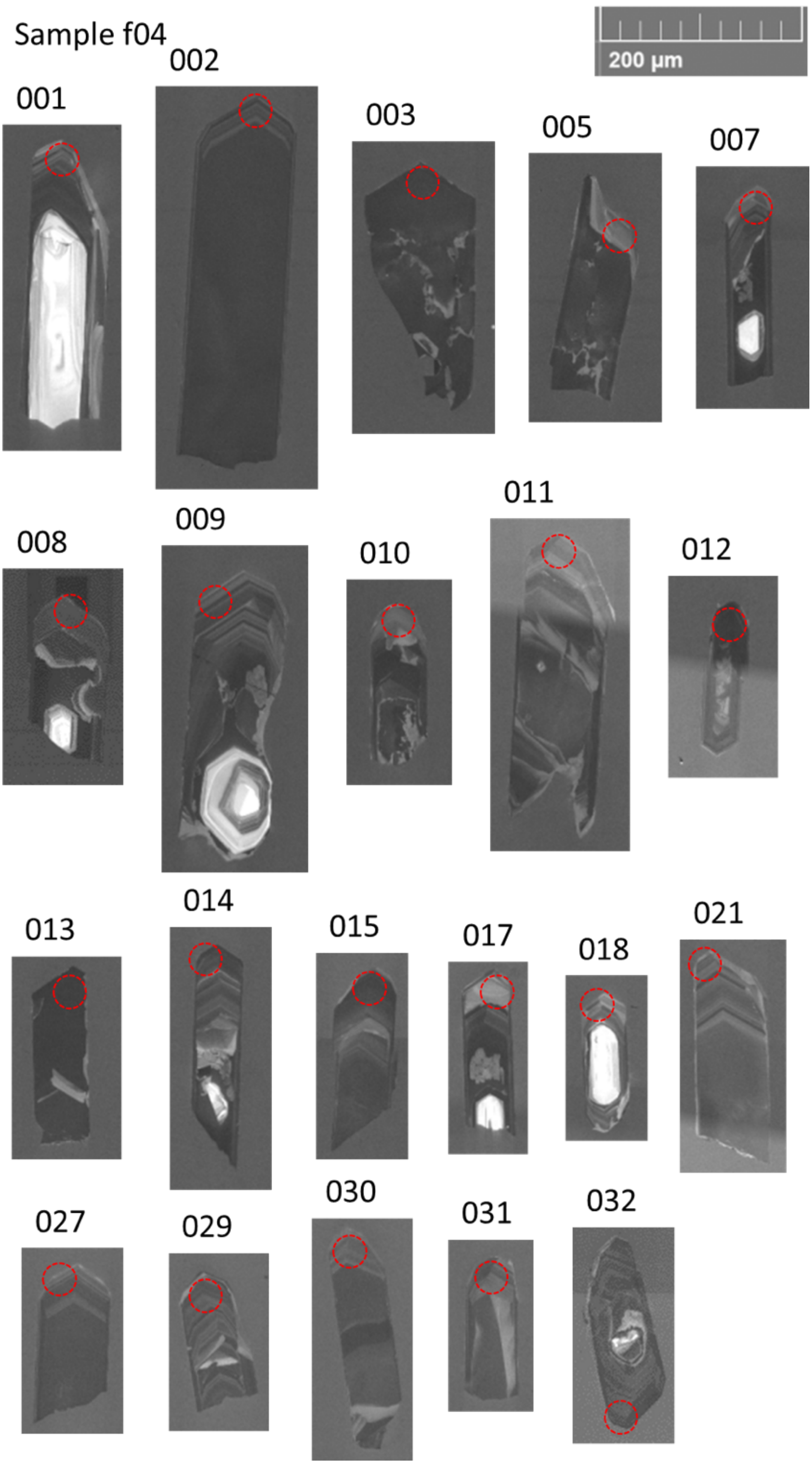
Sample f04
200 µm
001
002
003
005
007
008
009
010
011
012
013
014
015
017
018
021
027
029
030
031
032

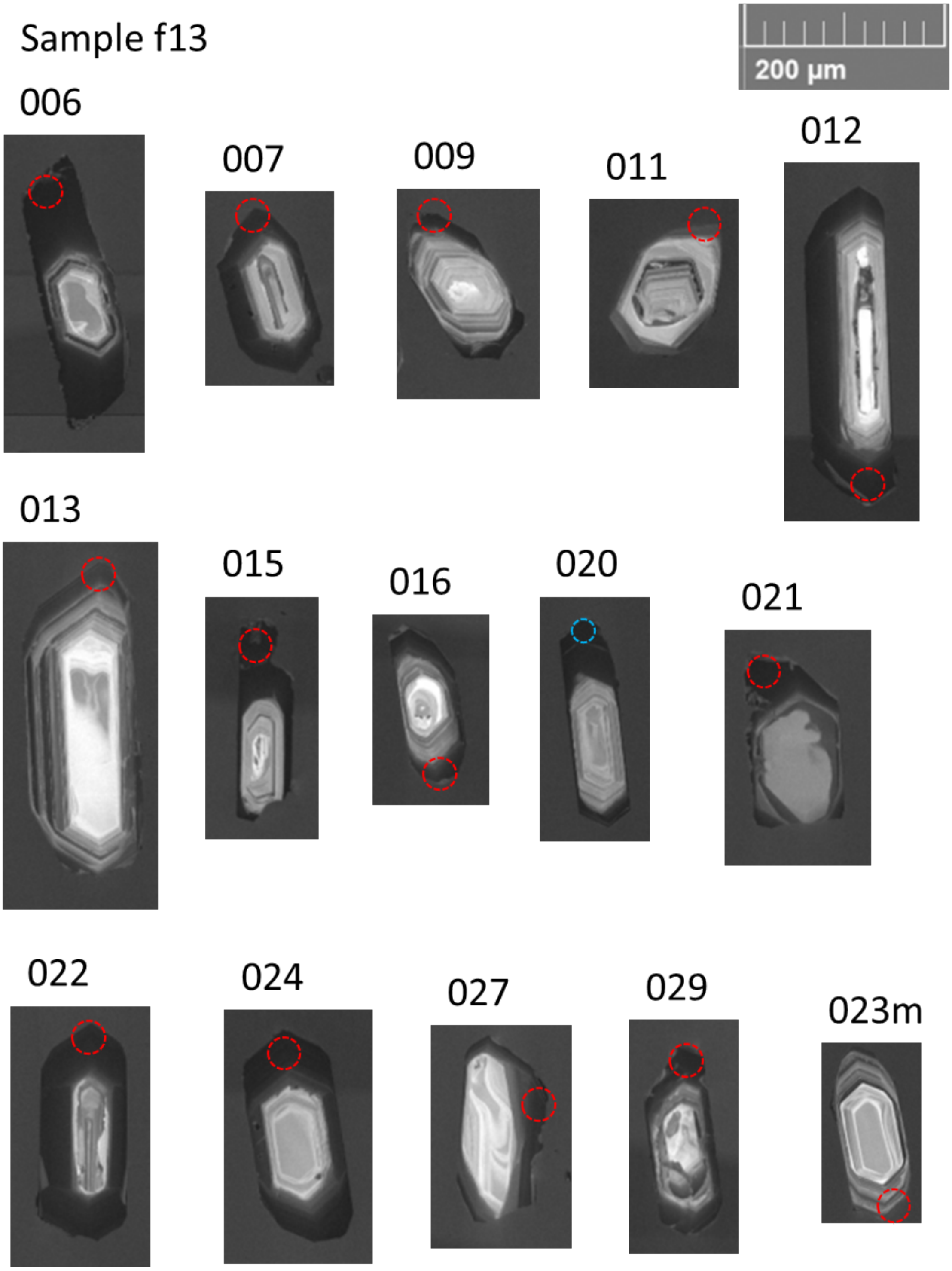
Sample f13
200 µm
006
007
009
011
012
013
015
016
020
021
022
024
027
029
023m

Part 2: Monazite

Ce, Th, and Y EDS maps for monazite grains from each sample analysed with U-Th-Pb ages relevant to the Himalayan orogen. Red dashed circles mark the U-Th-Pb analysis laser ablation pit (~11 µm). The colour scale is in counts per second (cps) at the following scale:

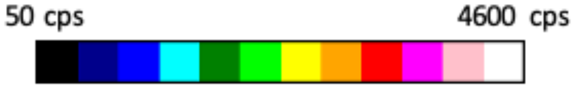

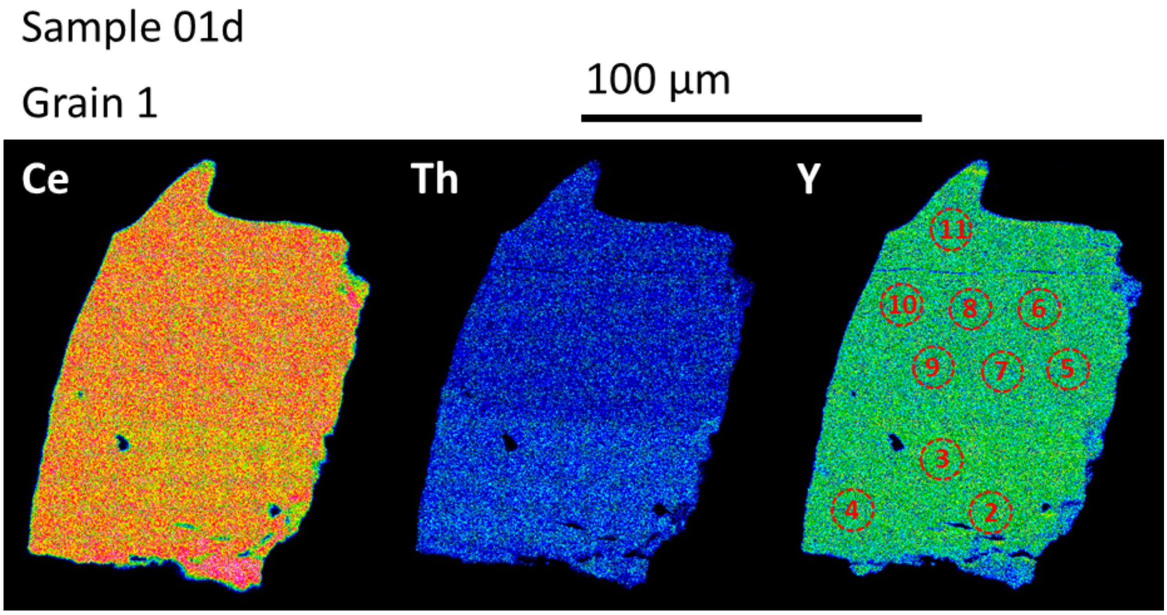

Sample 02a

Grain 1

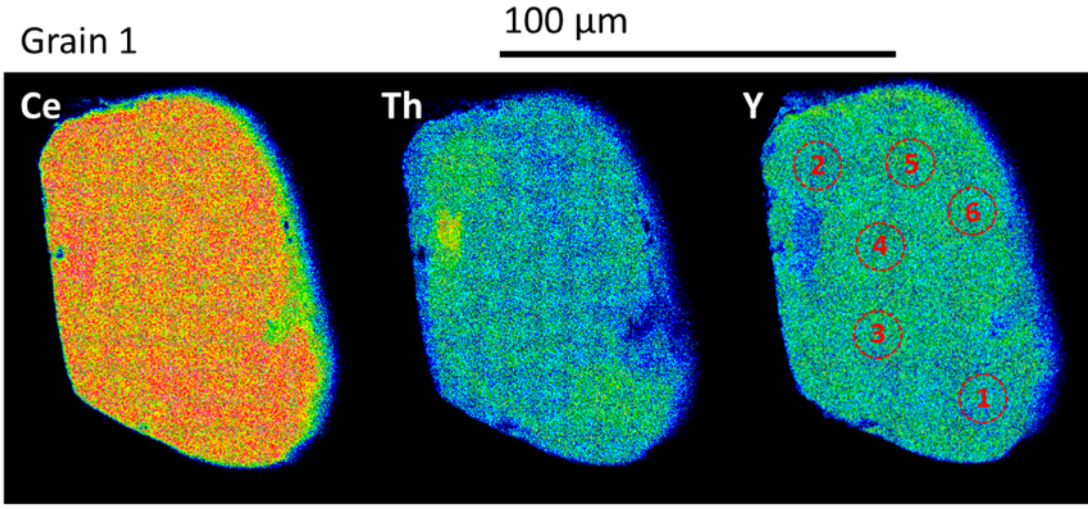

Grain 2

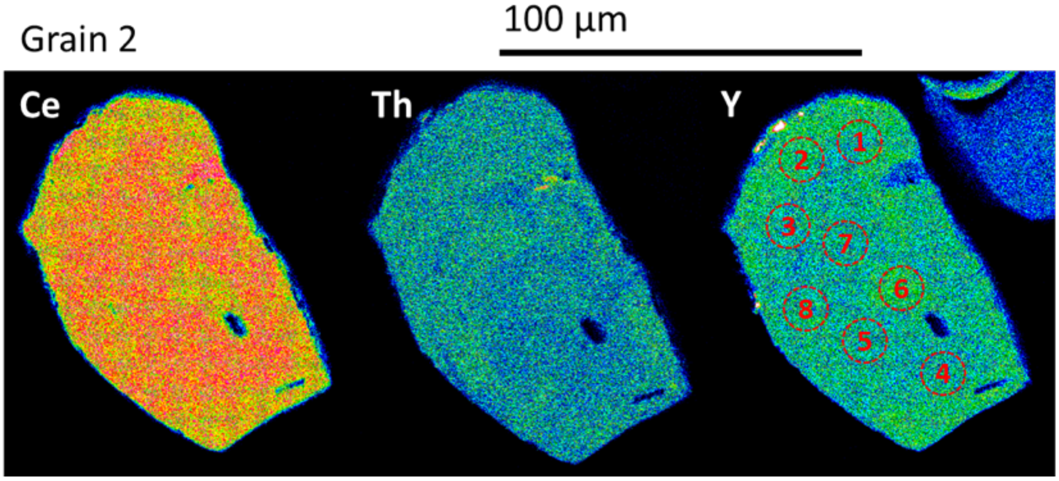

Sample 02b

Grain 1

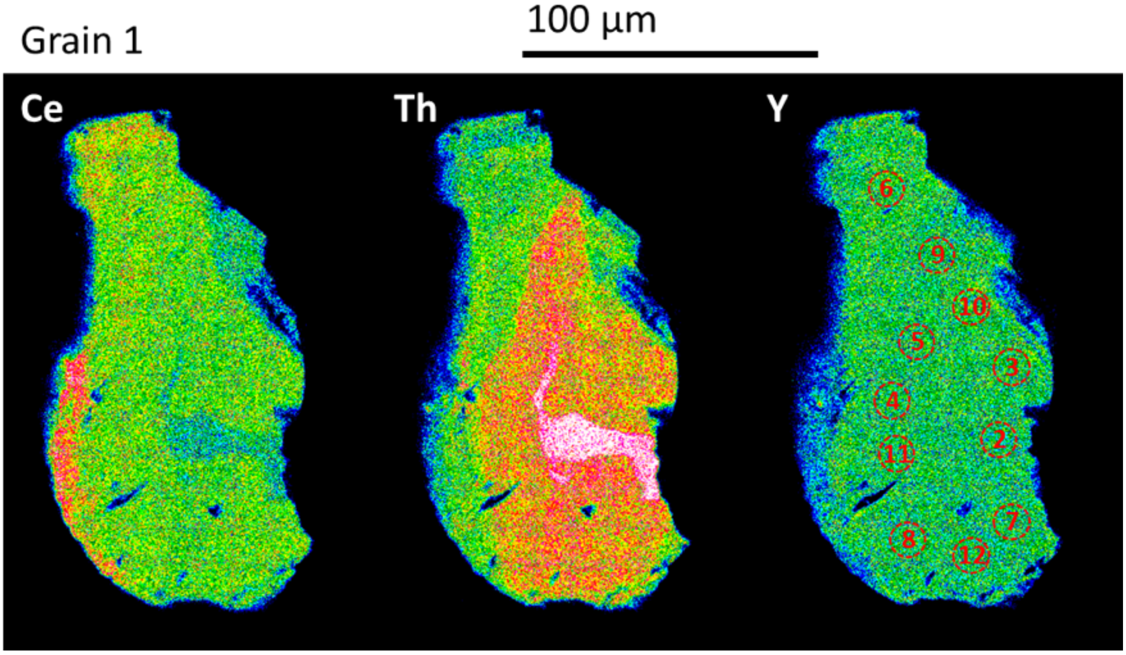

Sample 04a

Grain 1

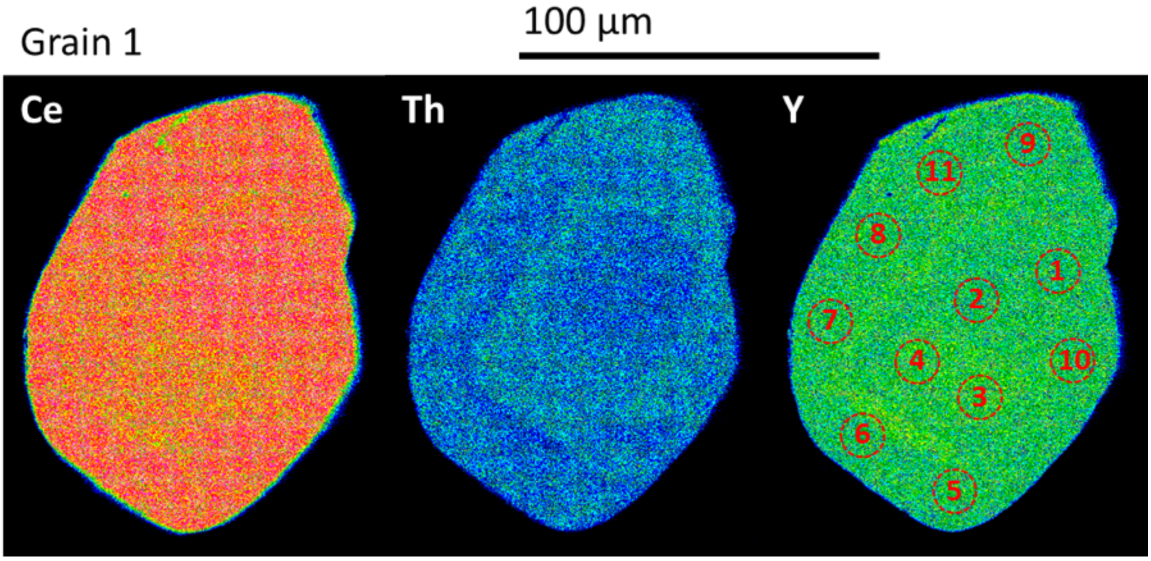

Sample 04b

Grain 1

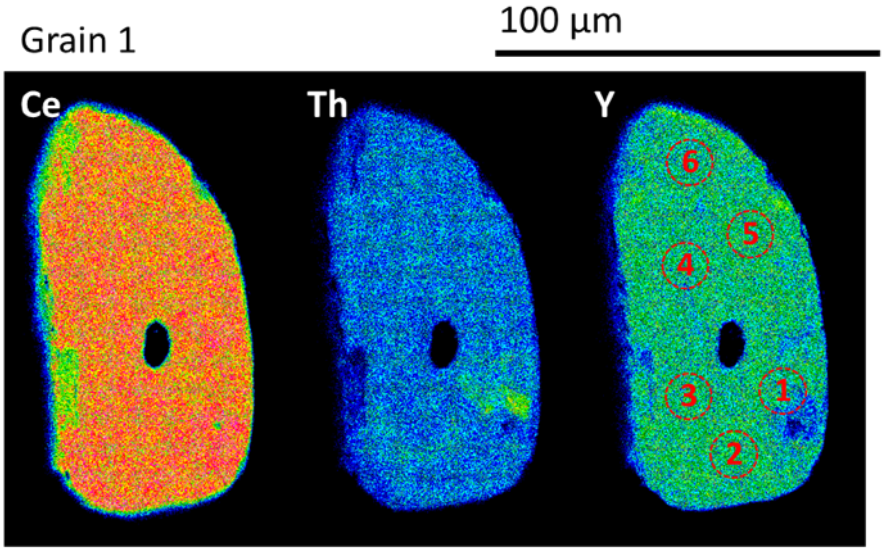

Grain 2

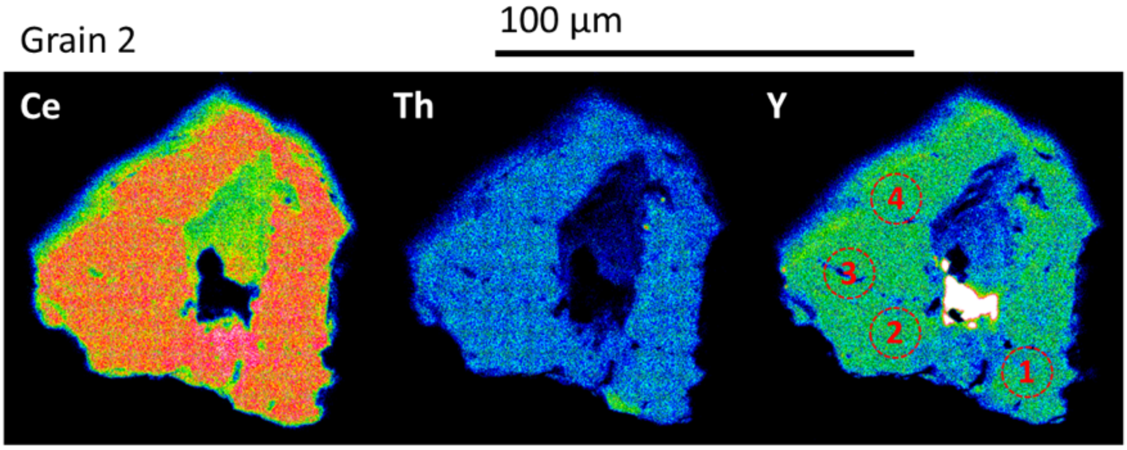

Grain 3

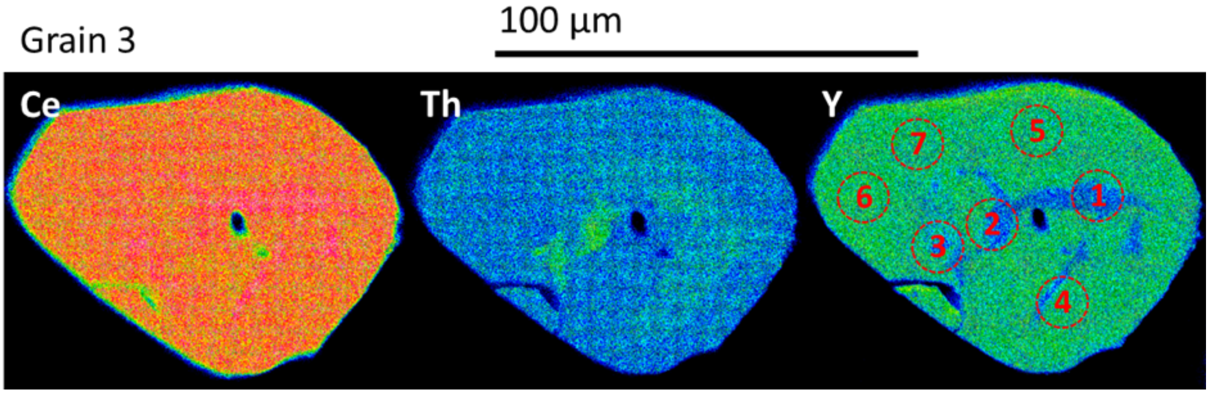

Sample 05b

Grain 1

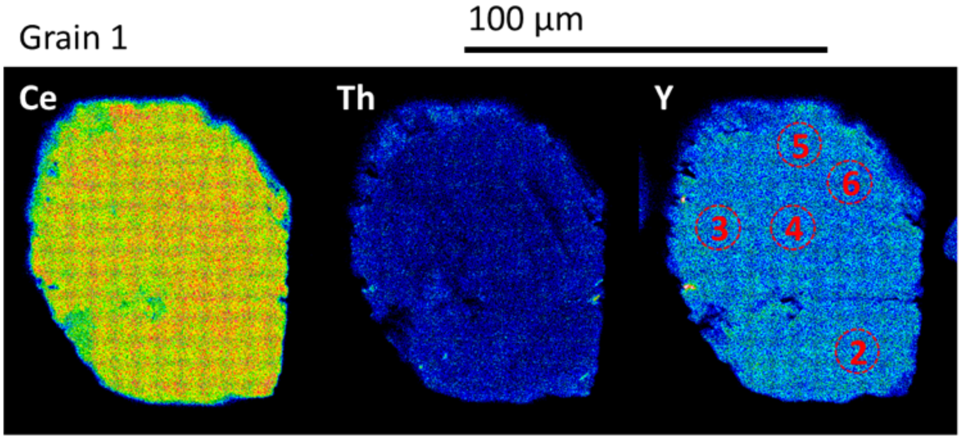

Grain 2

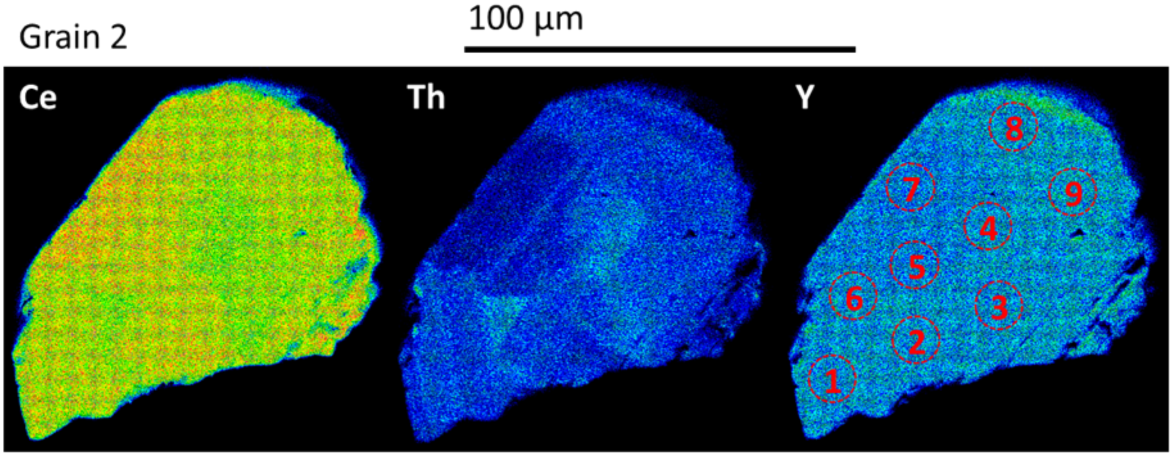

Grain 3

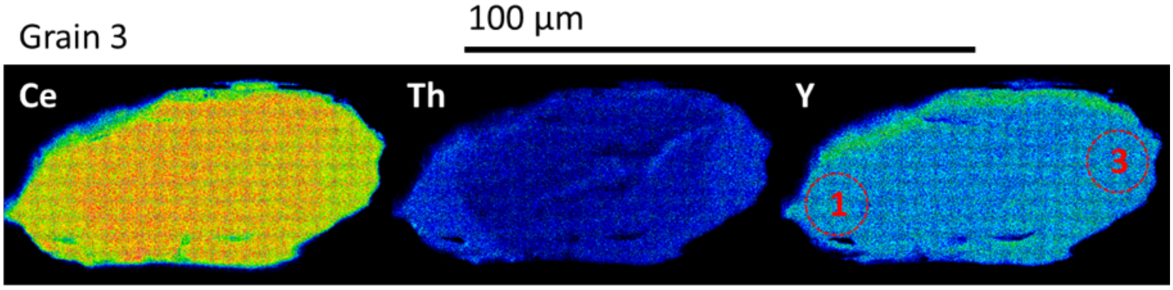

Grain 3

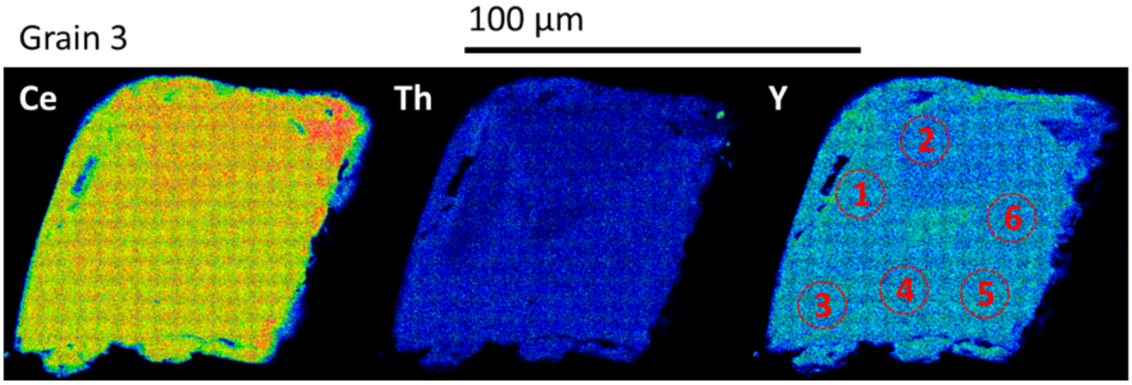

Sample 05b cont.

Grain 5

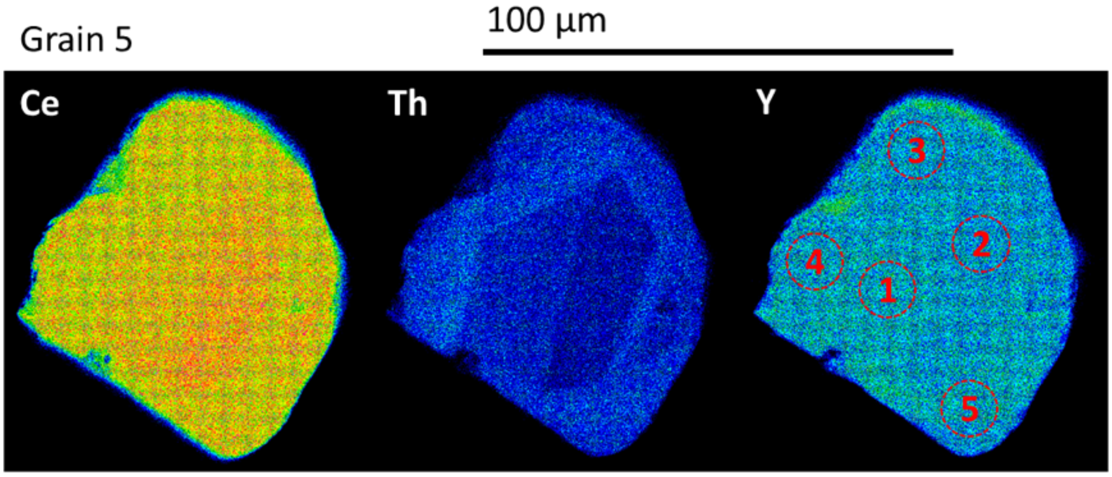

Grain 6

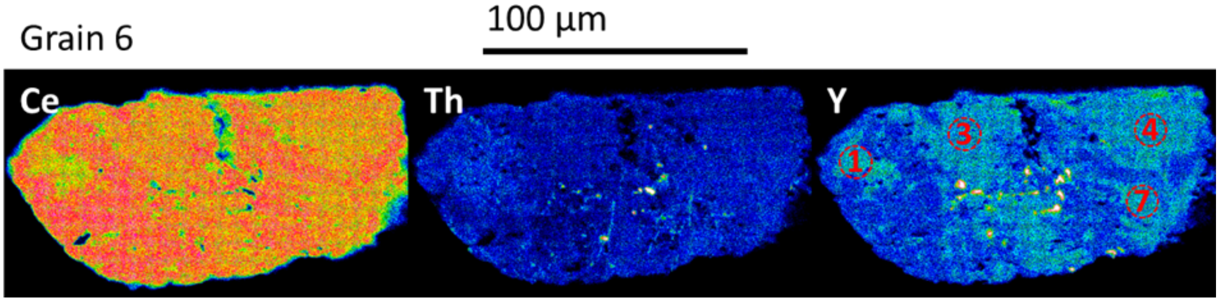

Sample 06

Grain 1

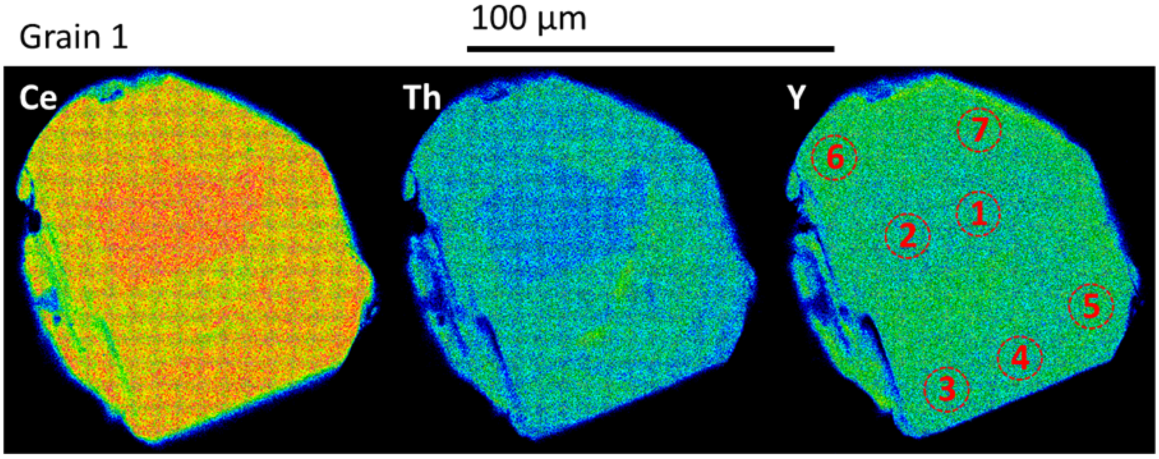

Grain 2

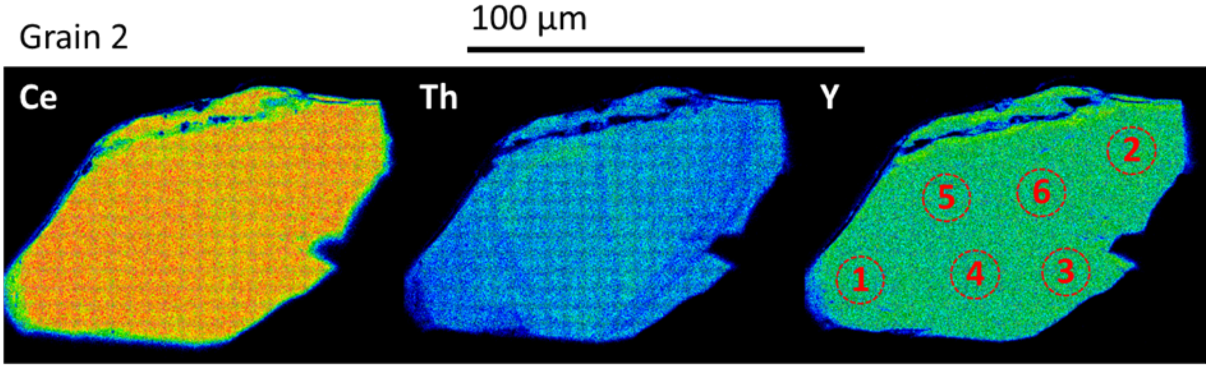

Grain 3

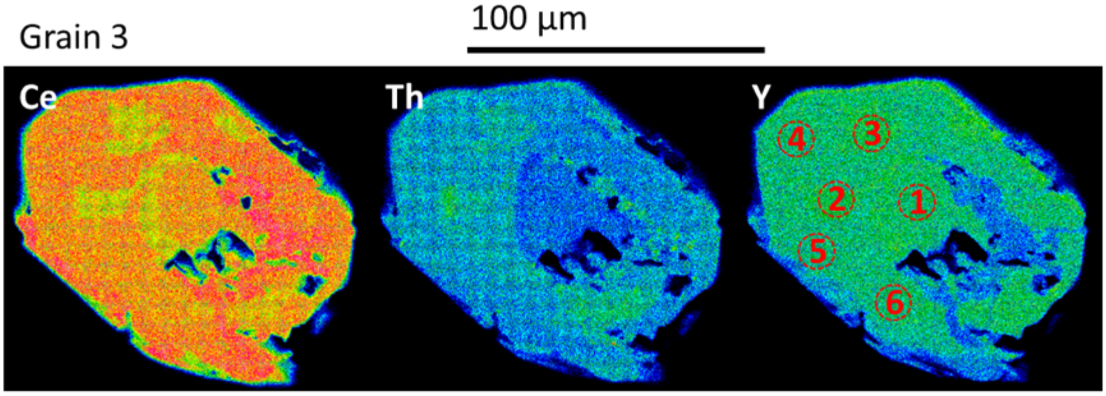

Grain 4

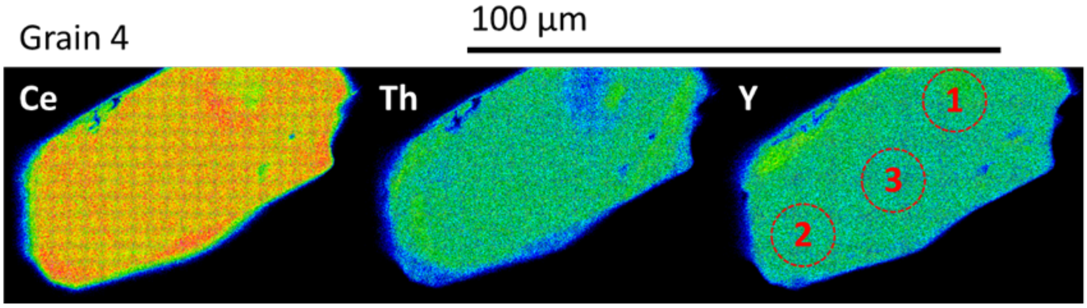

Sample 06 cont.

Grain 5

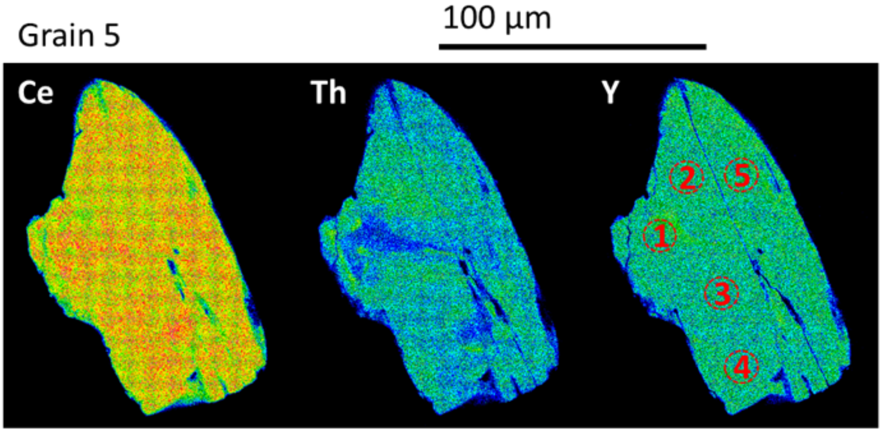

Grain 6

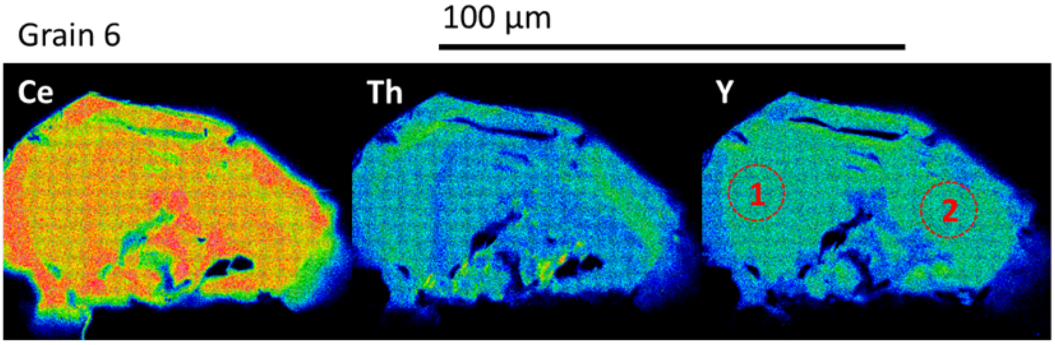

Grain 7

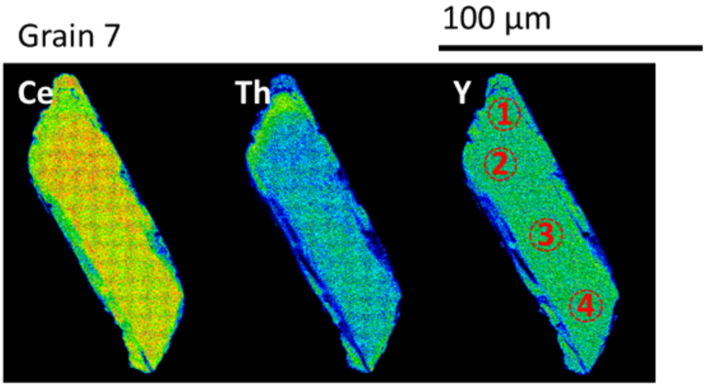

Grain 8

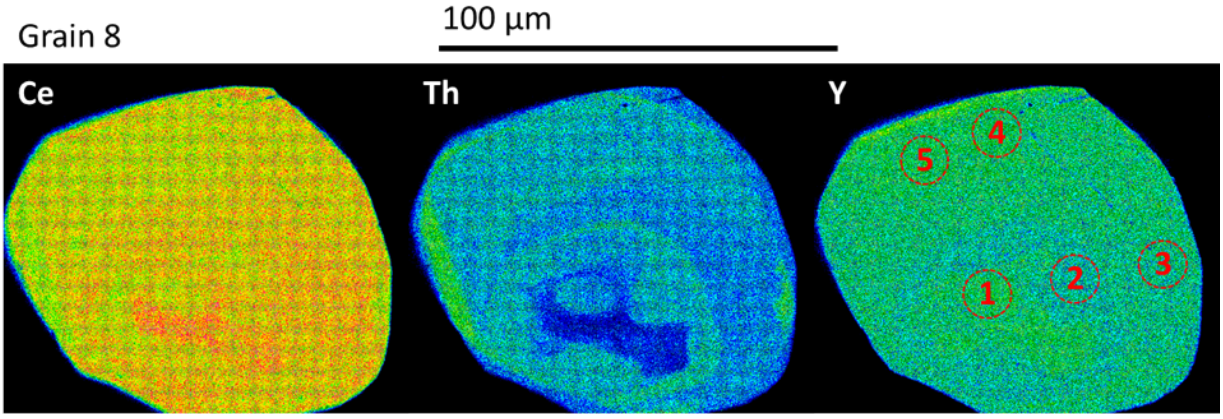

Sample 06 cont.

Grain 9

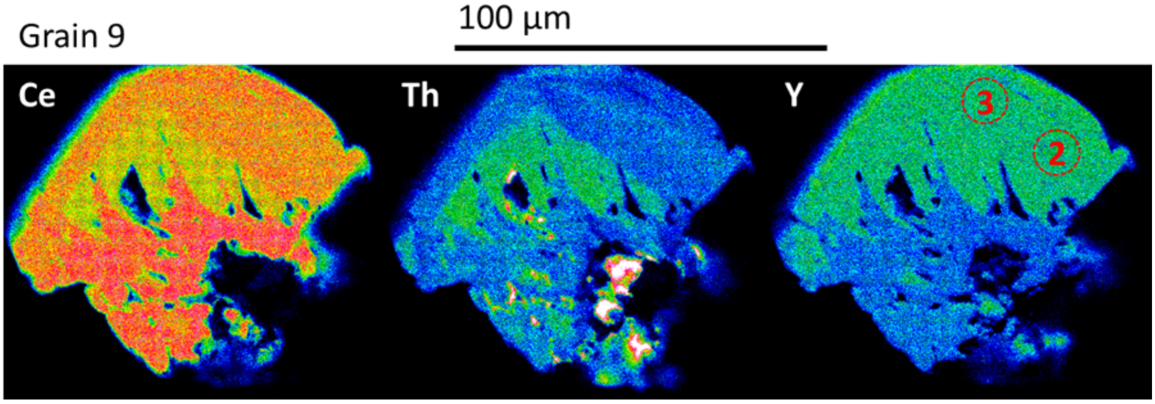

Sample 07c

Grain 1

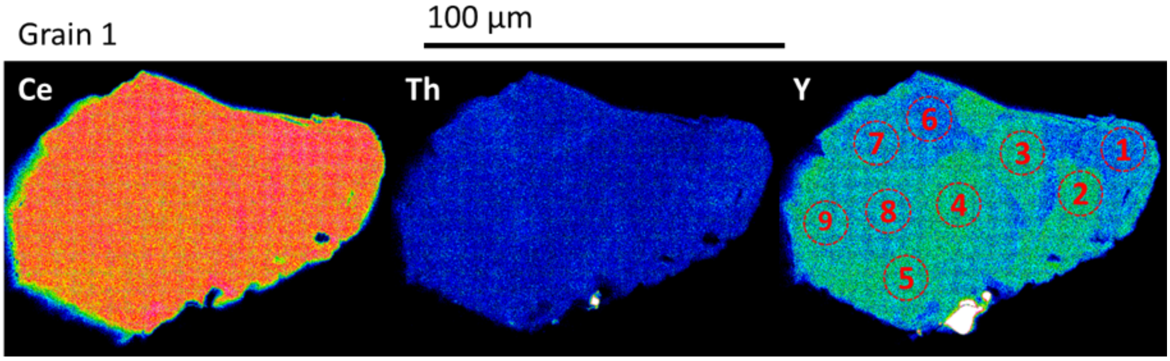

Sample 09b

Grain 1

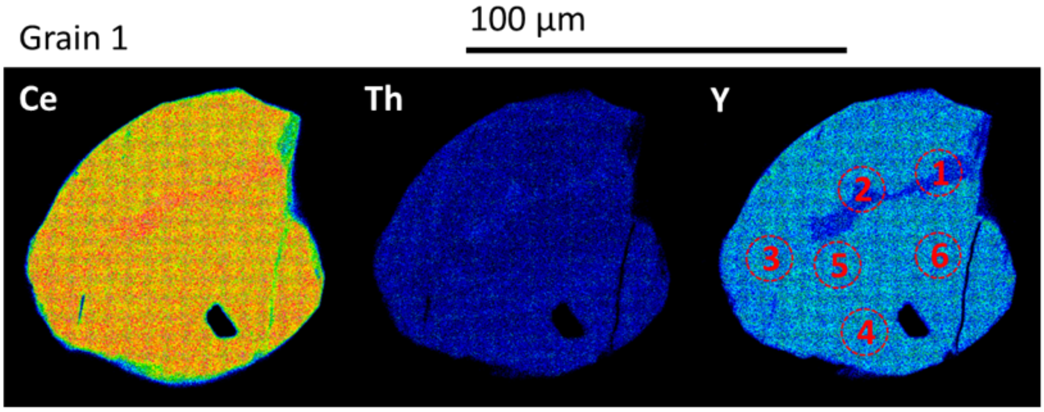

Grain 2

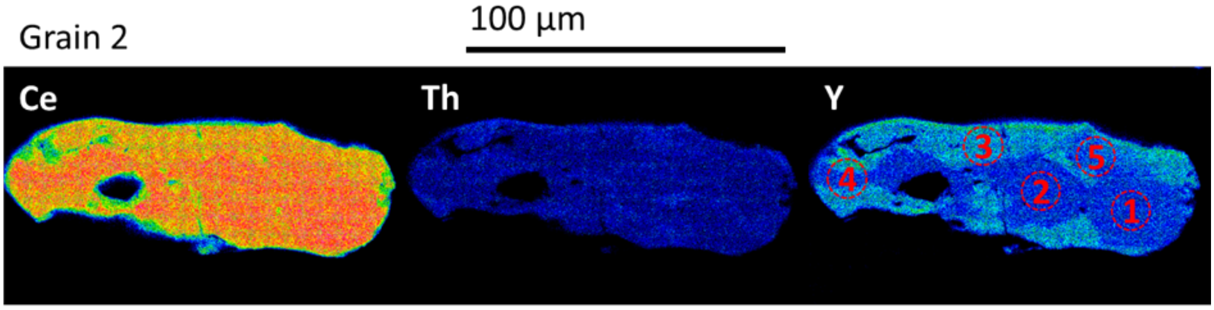

Grain 3

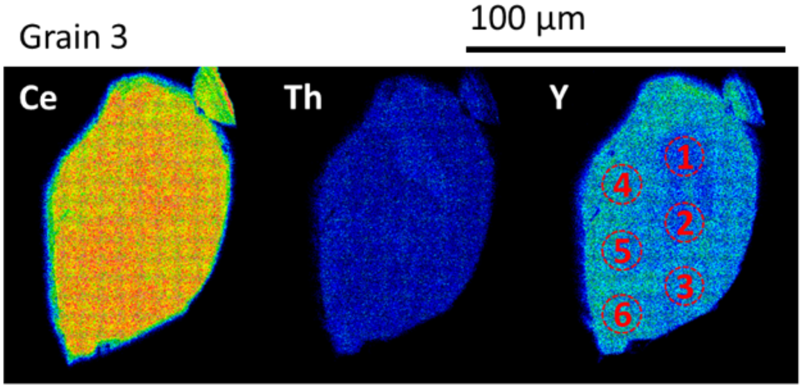

Grain 4

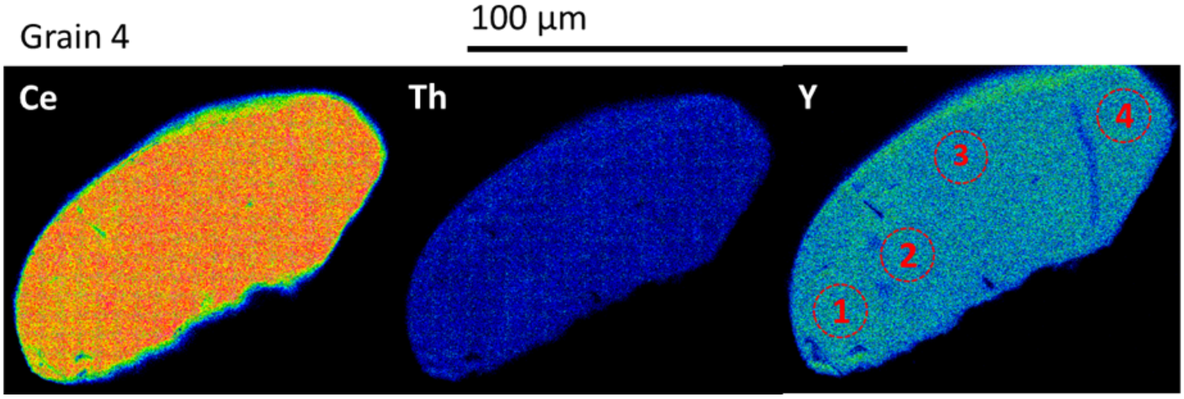

Sample 09b cont.

Grain 5

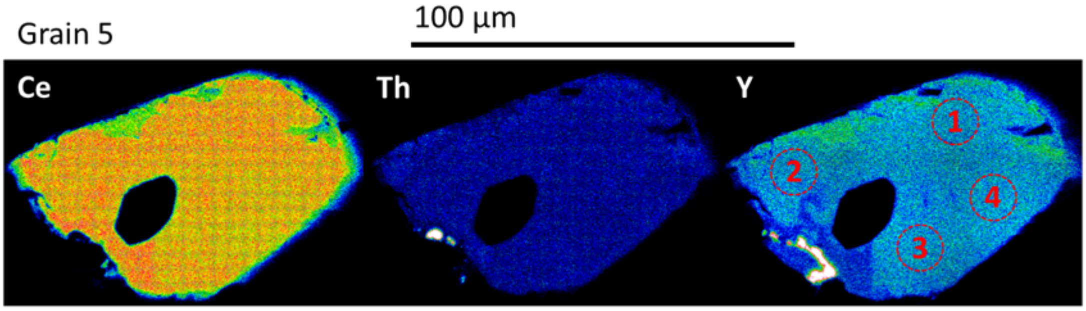

Grains 6 (left) & 7 (right)

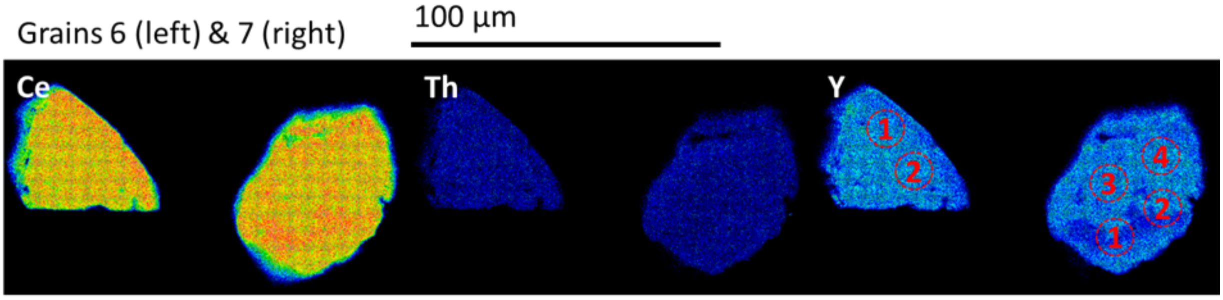

Grain 8

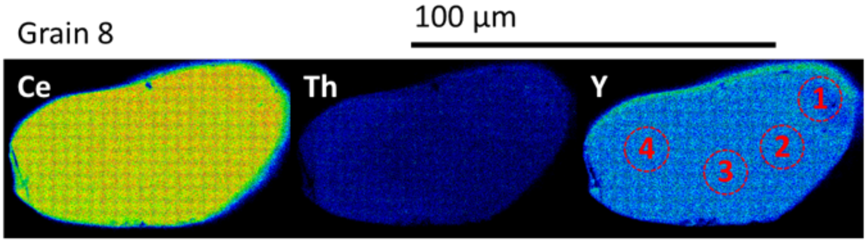

Grain 9

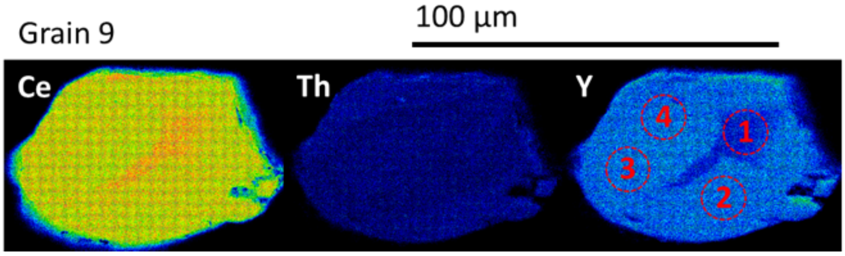

Grain 10

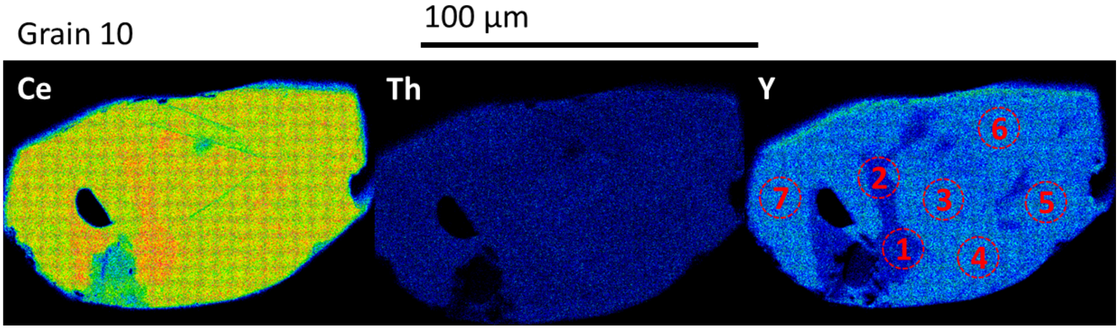

Sample f04

Grain 1

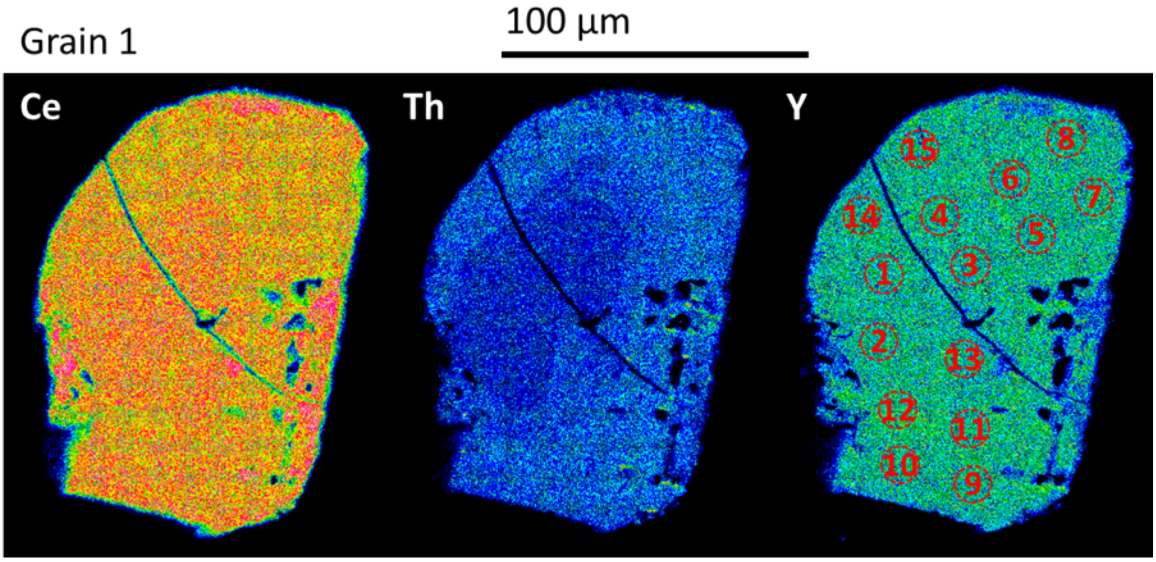

Sample f02

Grain 1

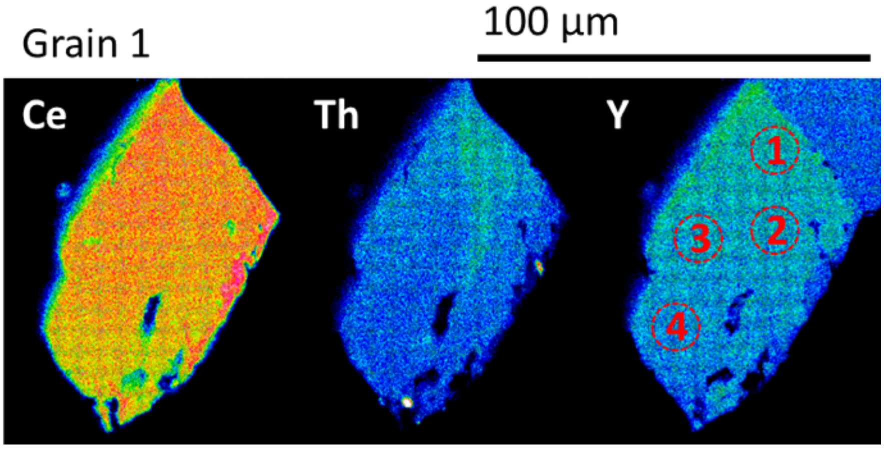

Sample f13

Grain 1

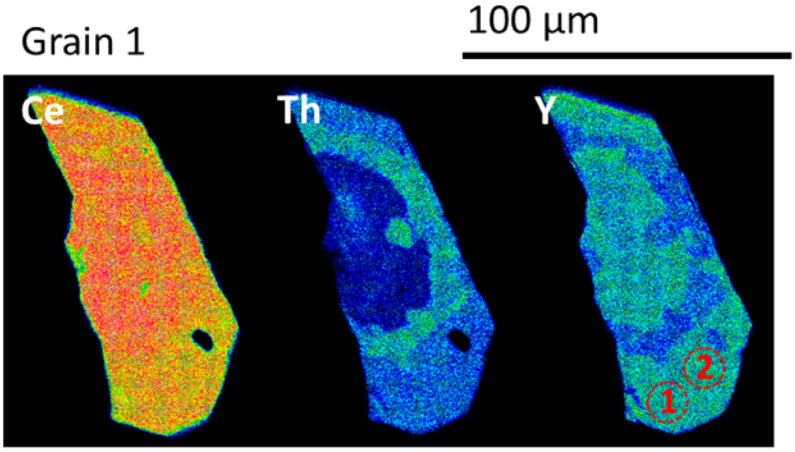

Grain 2

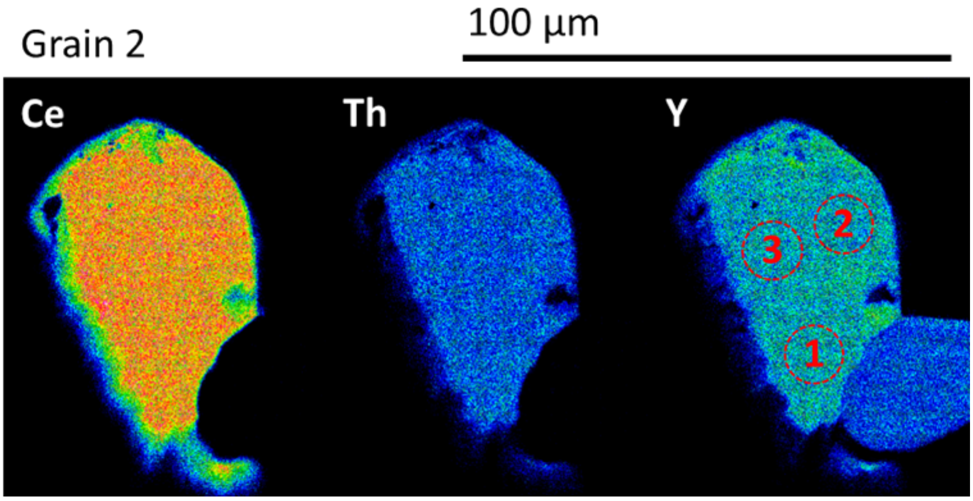

Supplement: Supplementary file 1 — Supplementary Material 1-3 Detailed method description & instrument metadata, Detailed sample description, Zircon and monazite spot analysis locations [file 410_2025_2247_MOESM1_ESM.pdf]
